# Development of a Micellar-Promoted Heck Reaction for the Synthesis of DNA-Encoded Libraries

**DOI:** 10.1021/acs.bioconjchem.3c00051

**Published:** 2023-03-08

**Authors:** Harriet A. Stanway-Gordon, Jake A. Odger, Michael J. Waring

**Affiliations:** Cancer Research Horizons Therapeutic Innovation, Chemistry, School of Natural and Environmental Sciences, Bedson Building, Newcastle University, Newcastle upon Tyne, NE1 7RU, United Kingdom

## Abstract

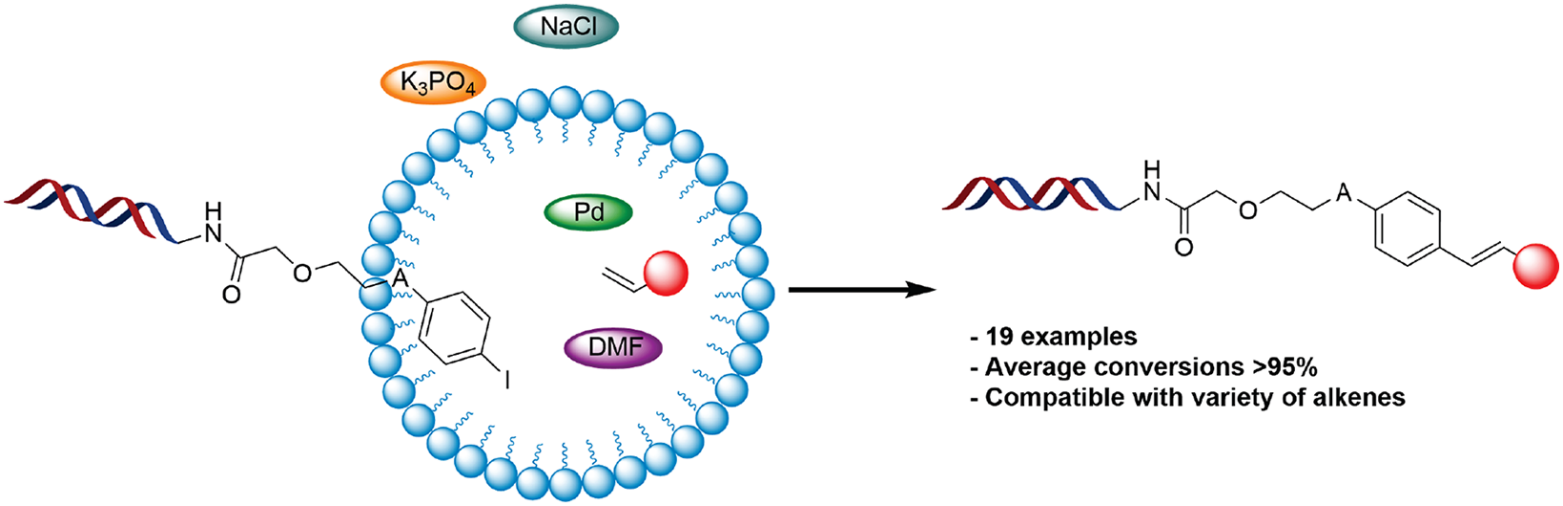

The capability of DNA encoded libraries (DELs) as a method of small molecule hit identification is becoming widely established in drug discovery. While their selection method offers advantages over more traditional means, DELs are limited by the chemistry that can be utilized to construct them. Significant advances in DNA compatible chemistry have been made over the past five years; however such procedures are still often burdened by substrate specificity and/or incomplete conversions, reducing the fidelity of the resulting libraries. One such reaction is the Heck coupling, for which current DNA-compatible protocols are somewhat unreliable. Utilizing micellar technology, we have developed a highly efficient DNA-compatible Heck reaction that proceeds on average to 95% conversion to product across a broad variety of structurally significant building blocks and multiple DNA conjugates. This work continues the application of micellar catalysis to the development of widely applicable, effective DNA-compatible reactions for use in DELs.

## Introduction

**Figure 1:**
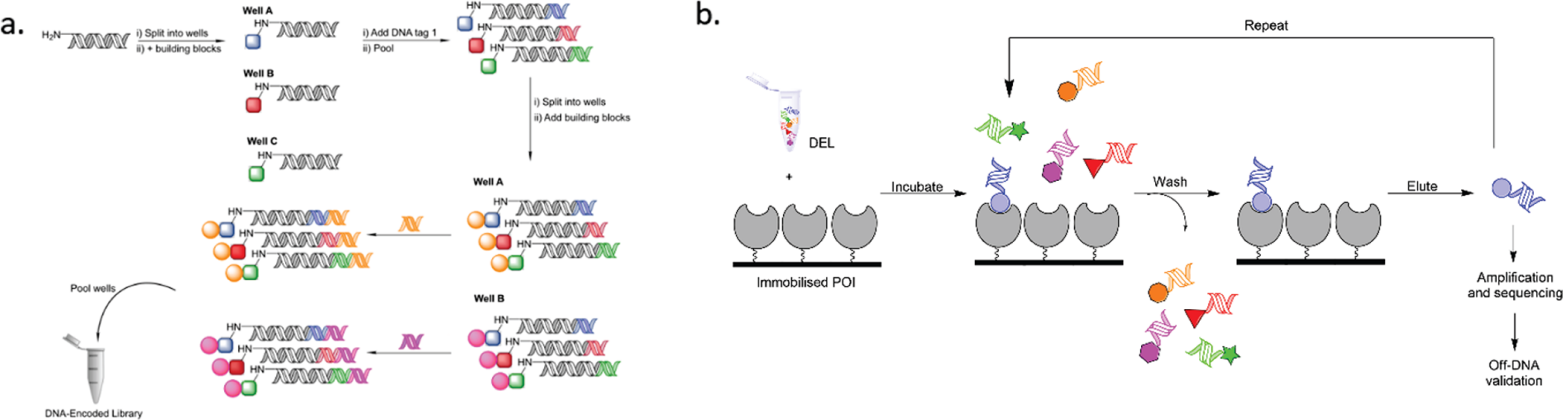
(a) General scheme for split-and-pool DEL synthesis; (b) Schematic for affinity screening of DELs with a solid-supported protein.

Since their conception in 1992, DNA encoded libraries (DELs) have emerged as a promising approach to hit identification within drug discovery.^1−4^ In DELs, large libraries of compounds (in excess of 10^6^ members) are synthesized attached to an encoding oligonucleotide sequence that can be used as a method of identification. Numerous approaches to the construction of DELs have been developed. The most commonly employed is sequence recorded split-and-pool combinatorial synthesis (Figure 1a).^5,6^ This method involves the sequential addition of chemical building blocks to an oligonucleotide strand, which are each encoded through the use of enzymatic ligation of a unique DNA-sequence. Through multiple rounds of chemistry and ligation protocols, each member of the library can be constructed with a sequence specific to its structure. The resulting libraries can then be pooled and screened, typically via affinity selection (Figure 1b), and binding ligands can then be deconvoluted through the use of PCR amplification and sequencing of the DNA-tag. Results are subsequently validated through the resynthesis and selection of identified hits in the absence of DNA. To date, this methodology has been successfully employed to identify multiple ligands for a variety of proteins, with several of these candidates progressing into clinical trials.^5^

Although advantageous over conventional methods of hit identification with respect to the rapidity with which both the synthesis and screening of DELs can be achieved, the process suffers from limitations with regards to the chemistry that can be employed in their construction. DNA-compatible chemistry is required to operate under aqueous conditions, at high-dilutions and, most crucially, maintain the integrity of the oligonucleotide tag. Additionally, for maximum library fidelity, the reactions should be high yielding, with high conversions and minimal side products, and compatible with a broad scope of building blocks to allow for maximum diversity and scale of the libraries that can be constructed. While advancements have been made in the past five years regarding accessible chemistry for application to DELs, the adherence to these criteria is often suboptimal in many literature examples, leading to increasing difficulties identifying hits isolated from libraries constructed utilizing such procedures.^7^

The use of cross-coupling reactions within medicinal chemistry programs is widely established, with multiple Pd-catalyzed couplings appearing in the top 20 reactions utilized within the field in 2014.^8^ A variety of these have been extensively studied on DNA including Suzuki–Miyaura,^9−15^ Buchwald–Hartwig^16−18^ and Sonogashira^9,14,15^ reactions, but the Heck reaction has received considerably less attention.^19^

**Figure 2:**
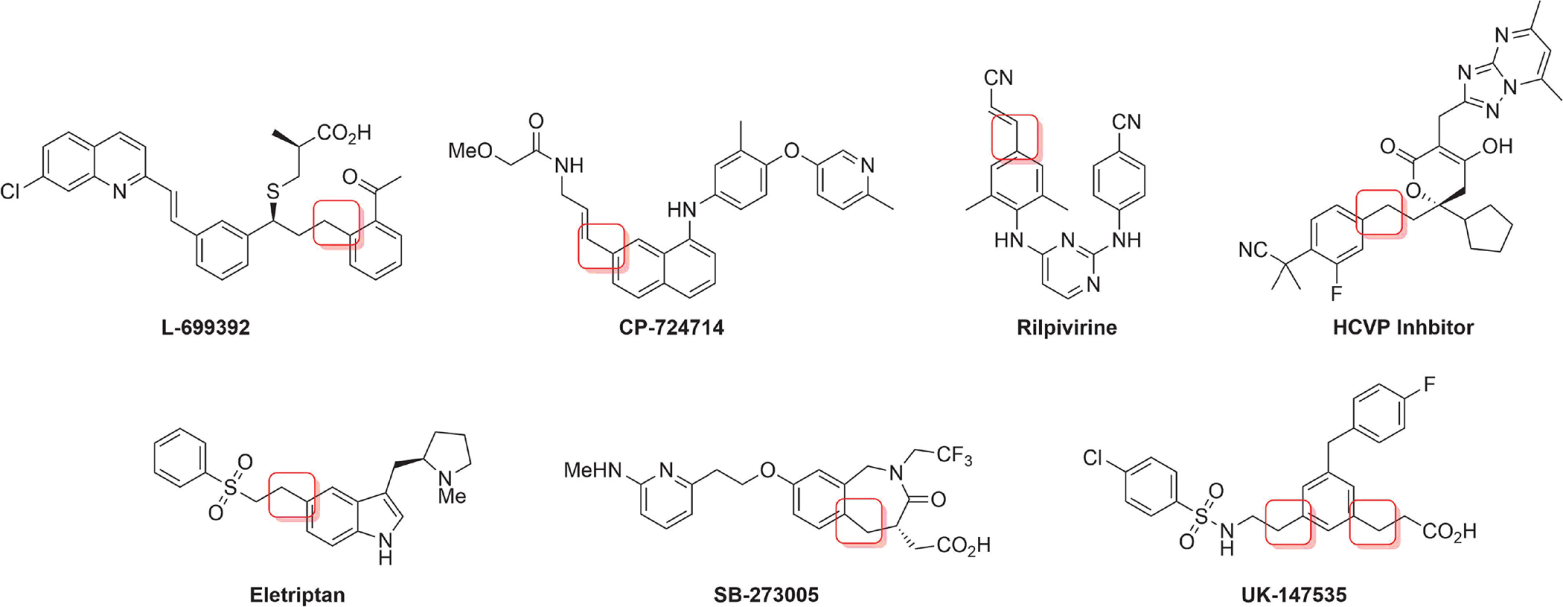
Approved drugs and clinical candidates that employ the Heck reaction in their synthesis; Heck C–C construction highlighted.^20^

In conventional (off-DNA) synthesis, the Heck reaction has been employed in routes to a variety of clinical candidates and pharmaceutical agents (Figure 2).^20^ Its application to library synthesis shows significant promise as it allows the construction of new C–C bonds in a modular manner, typically requires mild conditions and generally gives high yields.^21^

Current Heck methodology with DELs is somewhat limited; the first reports of a Heck reaction employed on-DNA were made by Liu et al. within the application of DNA-templated synthesis (DTS) libraries in 2002.^22^ With modest (26–54%) conversion achieved across four substrates, the group subsequently reported an alternative procedure in 2006 that allowed the formation of the Heck product in 80–85% conversion across three distinct DNA-architectures when conducted in 95% THF.^23^

**Figure 3:**
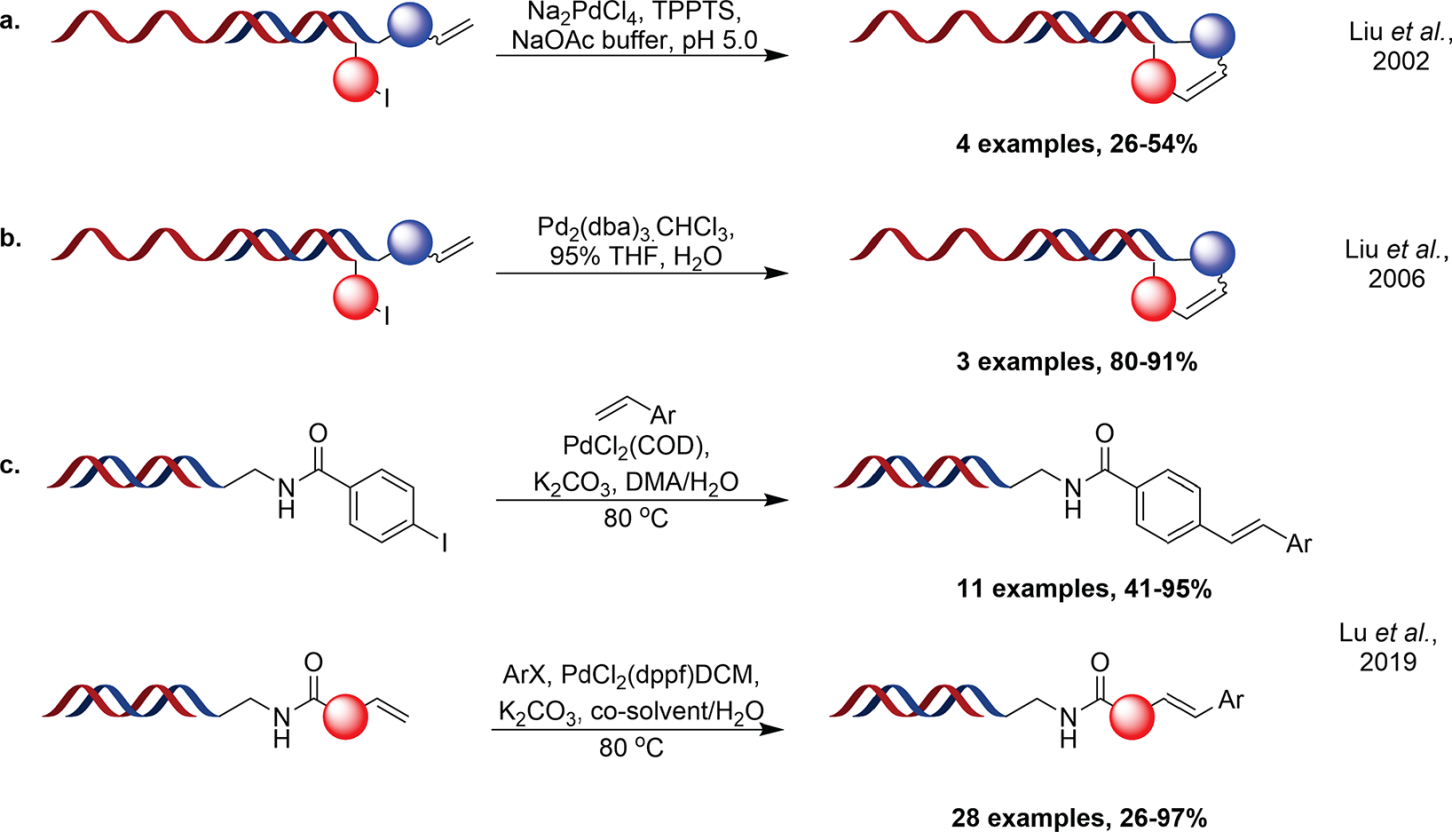
Currently reported DNA-compatible Heck procedures.^22−24^

For sequence recorded combinatorial libraries, current techniques are equally sparse with a sole publication reporting a variety of headpiece (HP) specific conditions that show varying conversions to desired product.^24^ While the methodology shows some strengths, in that it allows for the transformation to be achieved across a broader variety of substrates, the conversions achieved are less than ideal and offer significant room for improvement. For the ”forward” reaction (Ar-X on DNA) an average of 77% conversion was observed across a structurally limited set of substituted styryl derivatives. Similarly for the ”reverse” Heck (alkene on-DNA), average conversions across 14 substrates and two distinct oligonucleotides was 75% with a range of 26–95% (Figure 3).

We have previously shown that the application of micellar catalysis within DNA-compatible chemistry can be of great benefit, with highly efficient transformations achieved for several Pd-catalyzed reactions^25−27^ alongside various other modifications.^28,29^ Expanding upon this approach, we report the development of a micellar promoted Heck reaction for implementation within DELs that yields on average 95% conversion to product across a broad variety of structurally significant building blocks and multiple DNA conjugates.

## Results and Discussion

An aryl iodide substrate was synthesized and conjugated to a noncovalently linked 14-mer double stranded (ds-) oligonucleotide employing established methodology (**HP-1**, Supporting Information). Initial attempts to directly apply the literature protocol^30,31^ for the off-DNA micellar Heck reaction to the coupling between **HP-1** and various alkenes was unsuccessful. Poor conversions were obtained with substantial loss of DNA, which presented as a low ion count in conjunction with significant imbalance between the ratios of complementary and substrate-bearing strands (hereafter referred to as ”strand mismatch”), suggesting that an alternative strategy was required (data not shown). Control reactions to determine the cause of this phenomenon, consisting of incubation of **HP-1** in the presence of either Pd_2_(dba)_3_ or Pd(dtbpf)Cl_2_ suggested that the use of the Pd(II) precatalyst was responsible (Figure S3). Accordingly initial optimization focused on the use of a Pd(0) precatalyst, as the use of this in addition to a postreaction scavenger treatment (sodium diethyldithiocarbamate) appeared to protect DNA integrity. Employing **HP-1** and N-ethylacrylamide, screening of a variety of phosphine ligands was conducted to deduce the optimal species for the coupling; selection of ligands was influenced by reports relating to those that formed complexes that permitted the Heck reaction to occur in >70% yield between aryl bromides and vinyl/styryl derivatives at room temperature (Table 1).^32^

**Table 1:**
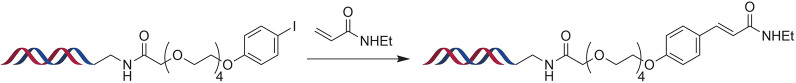
Results of Ligand Screen Applied to Heck Reaction between **HP-1** and N-Ethylacrylamide^a^

Entry	Catalyst	Ligand	Product (%)	SM (%)
1	Pd_2_(dba)_3_	CataCXium A	11	89
2	Pd_2_(dba)_3_	P(o-tol)_3_	7	93
3	Pd_2_(dba)_3_	QPhos	16	84
4	Pd_2_(dba)_3_	XPhos	37	63
5	Pd_2_(dba)_3_	JohnPhos	29	71
6	Pd[P(o-tol)_3_]_2_Cl_2_	N/A	0	100
7	Pd[P(^*t*^Bu)_3_]_2_	N/A	27	73

a General reaction conditions: 2 nmol **HP-1**, 7.33 mM “Pd”, 14.66 mM “L”, 5.3 mM K_3_PO_4_, 500 mM *N*-ethylacrylamide, 2% TPGS-750-M (30 μL)/15% THF (4.5 μL), 60 ^*o*^C, 1 h.

While product formation was almost universally observed across the set of ligands explored, the results varied from poor to moderate with CataCXium A,^33^ P(o-tol)_3_, QPhos,^34^ and Pd[(P(o-tol)_3_]_2_Cl_2_ all failing to achieve 20% conversion. Pd[P(^*t*^Bu)_3_]_2_ and JohnPhos^35^ showed slight improvement of ca. 30% conversion; however XPhos^35^ yielded the most promising result with 37% of the desired coupling product obtained.

A number of additional factors were then individually investigated including concentrations of alkene, Pd/L, base, and surfactant (Table S2). While individually several of these factors appeared to show favorable reaction progression, when combined no overall improvement was observed. Such a phenomenon suggested the presence of nonlinear interactions. It was determined however that increasing the base concentration to 53 mM in conjunction with a decrease in N-ethylacrylamide concentration to 125 mM showed a favorable outcome, with 49% product formation now achievable under these conditions.

Subsequently the palladium source was investigated (Table 2); while previously identified as problematic, it was hoped that in conjunction with the postreaction scavenger treatment, the use of a Pd(II) precatalyst may now be feasible. A selection of Pd(II) sources were screened in conjunction with XPhos, alongside the Buchwald precatalyst XPhosPdG3.^36^ Gratifyingly all Pd sources yielded improved conversions when compared to Pd_2_(dba)_3_; PdCl_2_ and Pd(OAc)_2_ both allowed for product formation to be achieved in >86% conversion, with some residual starting material and dehalogenation present. In contrast, [(cin)PdCl]_2_ and XPhosPdG3 yielded full consumption of starting material, with the latter of these examples affording 100% product. Justification for this dramatic improvement upon moving away from Pd_2_(dba)_3_ is postulated to be related to the established equilibrium between Pd(dba)_2_L_2_ and the active catalyst PdL_2_ that is known to exist in Pd_*x*_(dba)_*y*_/L systems, in which the bulk of the material exists as the former, resulting in reduced reaction rates.^37^

**Table 2:**
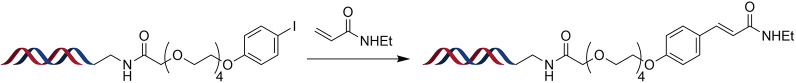
Results of Pd Screen Applied to Heck Reaction between **HP-1** and N-Ethylacrylamide^a^

Entry	Catalyst	Ligand	Product (%)	SM (%)	Dehal (%)
1	PdCl_2_	XPhos	86	10	4
2	Pd(OAc)_2_	XPhos	91	7	2
3	[(cin)PdCl]_2_	XPhos	95	0	5
4	XPhosPdG3	N/A	100	0	0

a General reaction conditions: 2 nmol **HP-1**, 7.33 mM “Pd”, 14.66 mM “L”, 5.3 mM K_3_PO_4_, 500 mM N-ethylacrylamide, 2% TPGS-750-M (30 μL)/15% THF (4.5 μL), 60 ^*o*^C, 1 h unless stated otherwise. Entry 4 relating to XPhosPdG3 contained no additional ligand resulting in overall reaction concentrations of 7.33 mM “Pd” and 7.33 mM XPhos.

**Figure 4:**
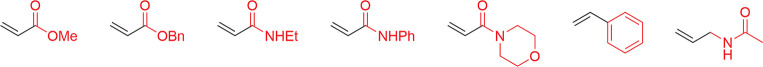
Original set of alkenes employed for determining general applicability of developed Heck conditions.

Reaction conditions were subsequently applied to the coupling between **HP-1** and a range of alkenes (Figure 4), While significant improvement in starting material consumption was observed across all substrates, the reaction conditions were far from universally applicable, with product formation varying from 15 to 100% across the set (Table S4). The predominant side product in all instances was dehalogenation of **HP-1**. It was hypothesized that this phenomenon was due to the reduced alkene concentration that had previously been used. While not shown to be detrimental in conjunction with Pd_2_(dba)_3_, most likely due to the aforementioned limited amount of active catalytic species, the employment of XPhosPdG3 was no longer subject to these same caveats and may benefit from this modification.

Attempts to further explore the effect of alkene concentration on reaction progression were undertaken with the poorest performing alkene, N-allylacetamide (Table S5). Encouragingly, improvement in product formation was observed at higher concentrations; however significant DNA strand mismatch was observed, causing concerns regarding the effects of the reaction on DNA-integrity. Since the combination of [(cinnamyl)PdCl]_2_/XPhos had yielded similarly promising conversions for the coupling with N-ethylacrylamide (95% product), reactions were repeated utilizing this catalytic system; however the issue persisted. Further repetition employing styrene as the coupling partner demonstrated that the observation was not substrate specific and suggested an issue regarding the DNA-compatibility of the conditions that required addressing prior to further optimization of the reaction.

**Figure 5:**
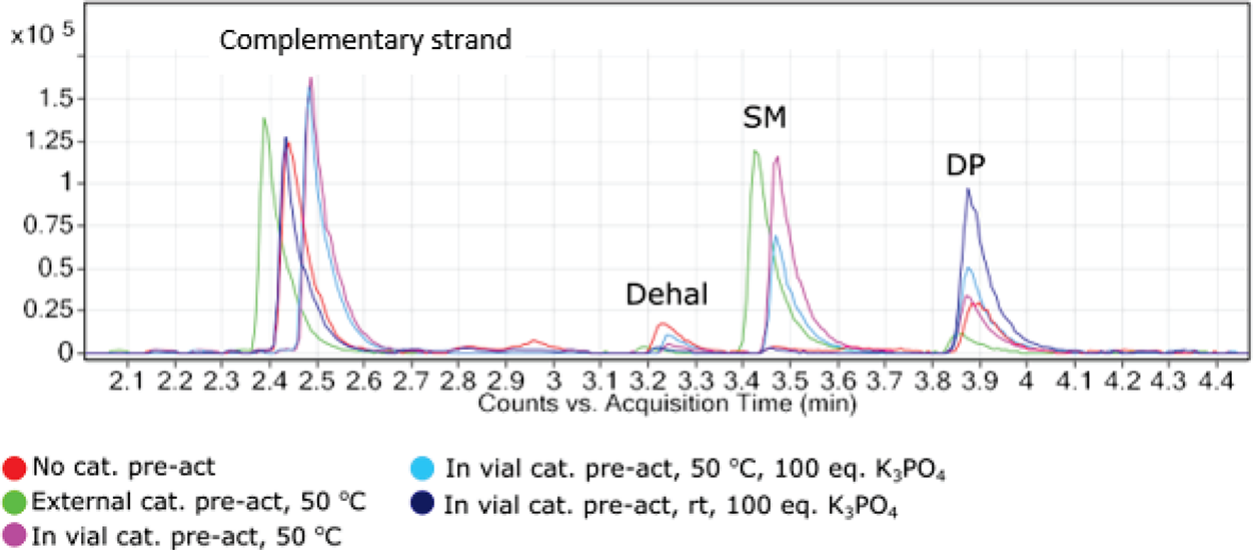
Overlay of differences in chromatograms obtained utilizing alternative modes of catalyst preactivation with [(cin)PdCl]_2_/XPhos. Both reaction progression and DNA integrity were improved through the incorporation and optimization of the procedure. DP = desired product.

It was theorized that there may be the potential to maximize DNA integrity through procedural modification and preactivation of the catalyst by mixing the Pd source, ligand, and base together prior to addition of the remaining components. Assembly of the reaction in this manner would minimize the initial amount of excess Pd(II) that could come into contact with the DNA during the reaction. Exploration into this modification was performed on the coupling of **HP-1** and styrene using both XPhosPdG3 and [(cin)PdCl]_2_/XPhos systems in conjunction with K_3_PO_4_ concentrations of 53 mM and 530 mM. Initial efforts toward this endeavor focused on external preactivation of the catalyst at 50 °C prior to addition to the reaction mixture (Table S6); while DNA integrity was significantly improved upon implementation of this procedure there was a deleterious effect on reaction progression with <25% conversion achieved across all experiments. Since this observation was believed to be due to issues regarding transfer of the catalyst, preactivation was then attempted within the reaction vial at the equivalent temperature prior to addition of the remaining components (Table S7). Again, DNA integrity was maintained and pleasingly reaction progression was improved; however product formation still failed to match preliminary results with only 42% achieved in the best instance ([(cin)PdCl]_2_/XPhos, 530 mM K_3_PO_4_). Further investigation with this catalytic system (Table S8) revealed that gratifyingly through performing preactivation at room temperature DNA-integrity could be achieved and reaction progression could be maintained with 98% product obtained in this last instance. Figure 5 demonstrates the observable differences in both DNA integrity and reaction progression across this series of experiments.

Extension of the conditions across the set of alkenes (Table S9) revealed that substrate specificity remained an issue and accordingly additional factors were explored. The use of cosolvents within micellar reactions is commonplace; originally employed in our DEL methodology to aid in the solubilization of more lipophilic reagents and to alter the micelle morphology, it was subsequently determined that the addition of a cosolvent alters the micellar diameter via organization of the solvent molecules within the micellar framework.^38^ Previous work conducted by ourselves has additionally demonstrated that the use of a co-ordinating cosolvent can be of great benefit with regard to improving the progression of Pd-based cross couplings via stabilization of the intermediate aryl palladium species.^27^ Several such solvents were screened for the coupling between **HP-1** and *N*-phenylacrylamide which had only yielded 2% product formation under the current conditions employing THF, namely, DMF, DMPU, and NMP (Table S10). All showed improvement in reaction progression with full consumption of starting material now achieved in all instances; reactions employing DMPU and NMP however displayed the emergence of several unidentified side products, and therefore DMF was determined to be most favorable. Expansion of these conditions across the seven alkenes was highly promising, with product formation now achieved in excess of 79% in all instances.

Again, employing the poorest performing alkenes (*N*-phenylacrylamide and benzyl acrylate), further investigations were performed to determine if greater improvement could be achieved (Tables S12 and S13). Exploration into temperature effects suggested that through reducing the temperature to 50 °C in conjunction with extending the reaction time to 2 h, increased levels of product formation could be achieved. Prior to exploring the influence of this modification on the extended substrate scope, several additional alterations to the system were investigated with the two substrates to determine their impact of the system. The ratio of Pd:L was deemed prudent to explore, since this could influence both the form of the catalytic species in solution, alongside the kinetics of the reaction; results of this investigation however showed no improvements over the original stoichiometry. Additionally, reports of off-DNA micellar promoted Heck reactions had reported an improvement in product formation when performed in the presence of NaCl.^39^ In a similar fashion to the use of cosolvents, NaCl influences the micellar framework, resulting in a ”salting out” effect in which the PEG region of the micelles is dehydrated, increasing the overall micellar diameter. The final factor under consideration was again the concentration of the alkene; due to dehalogenation being observed as the majority side-product, this was deemed prudent to explore in order to allay concerns that the concentrations previously examined were either side of an apex of optimal concentration. Results of these two latter modifications suggested that both the addition of NaCl and increased alkene concentration yielded positive outcomes for the two substrates under investigation.

**Figure 6:**
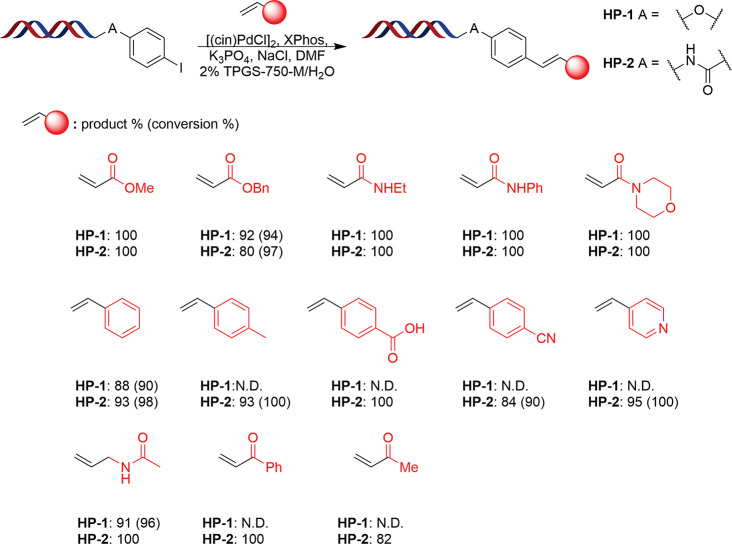
Results of application of optimized conditions to reaction between **HP-1/2** and a range of alkenes. Conditions: 2 nmol DNA, 3.67 mM [(cin)PdCl]_2_, 14.66 mM XPhos, 530 mM K_3_PO_4_, 375 mM alkene, 1.6 M NaCl, 2% TPGS-750-M (30 μL)/15% DMF (4.5 μL), 50 °C, 2 h; [(cin)PdCl]_2_, XPhos and K_3_PO_4_ in DMF/H_2_O were stood at room temperature in the reaction vial for 10 min prior to addition of remaining reaction components.

Through the combination of these favorable factors, optimal conditions for the DNA-compatible Heck reaction were deduced and applied across a variety of substrates, all of which showed high levels of conversion to product in excess of 88% (average = 96%). Application of the conditions to a more conventional DEL-type headpiece **HP-2**, alongside extension to incorporate a variety of additional alkenes demonstrated the robustness of these conditions, with all products formed in excess of 80% and the average conversion maintained (Figure 6). The method was shown to be highly effective in the coupling of acrylamides and acrylate esters, with almost universal conversion to product across both of these classes. Expansion to a series of substituted styrenes exemplified that high levels of product formation could also be achieved with building blocks of this form (84–100%), including analogous heteroaromatic derivatives (4-vinylpyridine, 95%). Protected allylamines were also shown to be amenable to coupling using the methodology, with N-allylacetamide yielding in excess of 90% product for both electron-rich and electron-poor DNA conjugates. In the instances of coupling acrylophenone and methyl vinyl ketone, alongside formation of the product it was noted that some minor conjugate addition of the respective building block to the complementary strand was observed. Such a phenomenon is unlikely to impact the downstream applications of the libraries; however it is advised that validation of structurally similar building blocks should be performed prior to incorporation to assess the extent of this side-reaction.

**Figure 7:**
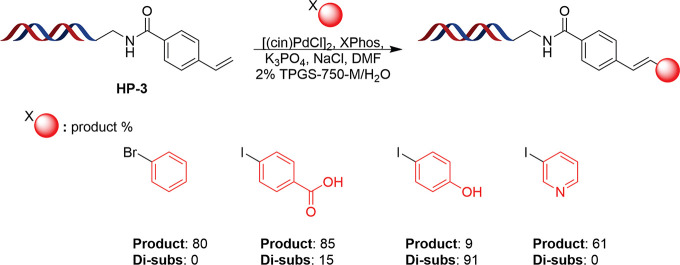
Results of application of optimized conditions to reaction between **HP-3** and a range of aryl halides. Conditions: 2 nmol DNA, 3.67 mM [(cin)PdCl]_2_, 14.66 mM XPhos, 530 mM K_3_PO_4_, 375 mM ArX, 1.6 M NaCl, 2% TPGS-750-M (30 μL)/15% DMF (4.5 μL), 50 °C, 2 h; [(cin)PdCl]_2_, XPhos and K_3_PO_4_ in DMF/H_2_O were stood at room temperature in the reaction vial for 10 min prior to addition of remaining reaction components.

Due to the successes achieved in the instance of couplings with DNA-aryl halide conjugates, it was deemed appropriate to explore if the conditions showed equal promise for the reverse reaction, i.e., DNA-alkenyl conjugates. Initially a DNA-construct containing a terminal acrylamide moiety was constructed for investigation of this transformation; however upon subjection to the Heck conditions, the sole product obtained was found to be the conjugate addition of dimethylamine most likely formed from DMF hydrolysis during the reaction (data not shown). An alternative DNA-styryl conjugate, **HP-3** was synthesized and limited exploration employing this headpiece demonstrated that the reactions were still reasonably efficient in this instance, with product formation in excess of 60% across a small set of 4 substrates (Figure 7). In contrast to the ”forward” reaction, disubstitution was an issue when the coupling was performed in this manner and several examples showed the presence of this outcome as either the minor or major product. While not ideal, the reaction does show promise in application for this procedure in spite of not being optimized for such a transformation and may offer benefit for select building blocks which can be identified during initial validation procedures.

The availability of Heck reactions for DEL-synthesis has previously been limited by the available methodology, with published work demonstrating substrate specificity and incomplete conversions. The application of micellar catalysis to the transformation and subsequent optimization has led to the identification of a highly efficient procedure that allows for an average of >95% product formation across a broad range of examples. In addition to allowing for improved levels of conversion across more structurally diverse building blocks, the procedure also requires significantly milder conditions than previously established work, operating at reduced temperatures and shorter times. Furthermore, again in contrast to the current precedent, a sole procedure can be employed for both DNA-ArX and DNA-styryl substrates in spite of optimization not being performed for the latter of these examples. This work further demonstrates the applicability of micellar-mediated transformations for DEL-synthesis. The procedure is in the process of being implemented into construction of a library and results will be reported in due course.

## Experimental for Optimized Heck Procedure

To a 50 μL glass insert for a Para-dox 96-well microphotoredox plate was added K_3_PO_4_ (8 μL, 113.2 mg in 200 μL H_2_O), [(cinnamyl)PdCl]_2_ (2.25 μL, 5.1 mg in 200 μL DMF), and XPhos (2.25 μL, 9.5 mg in 100 μL DMF). Samples were vortexed for 30 s then allowed to stand at room temperature for a further 10 min before addition of 5% TPGS-750-M (12 μL), aq. NaCl (8 μL, 6.14 M), DNA (2 μL, 1 mM in H_2_O), and alkene (11 μmol). Samples were then vortexed for a further 30 s and heated at 50 °C for 2 h in a Para-dox 96-well microphotoredox/optimization plate. Sodium diethyldithiocarbamate (6 μL, 1 M in H_2_O) was then added to the reactions, which were heated at 60 °C for a further 30 min. The reaction mixtures were diluted with H_2_O (up to 200 μL) and washed with DCM (3 × 400 μL). The organics were removed; aqueous solutions were then filtered through a hydrophilic PTFE filter and analyzed via mass spectrometry.

## Supporting Information Available

Detailed experimental procedures, further experimental results for optimization, and full analytical data (PDF)
Structures and spectra (ZIP)

## Abbreviations Used

DEL: DNA-encoded library
DNA: DNA
FED: factorial experimental design
HP: headpiece
