# Supplementary material for: Development of a Micellar-Promoted Heck Reaction for the Synthesis of DNA-Encoded Libraries
Source: Bioconjug Chem. 2023 Mar 8;34(4):756–63. doi: 10.1021/acs.bioconjchem.3c00051 (PMC10119937; doi:10.1021/acs.bioconjchem.3c00051)

Supporting Information: Development of a micellar-promoted Heck reaction for the synthesis of DNA-encoded libraries

Harriet A. Stanway-Gordon, Jake A. Odger and Michael J. Waring*

Cancer Research Horizons Therapeutic Innovation, Chemistry, School of Natural and Environmental Sciences, Bedson Building, Newcastle University, Newcastle upon Tyne, NE1 7RU, UK.

Contents

1 Solvents and Reagents **3**

2 Analytical Techniques **3**

3 Chromatography and Equipment **3**

4 Supplementary Figures and Tables **4**

4.1 Figure S1: Scheme for Linker Synthesis 4

4.2 Figure S2: Scheme for Construction of **HP-1** 4

4.3 Figure S3: Incubation of HP-1 with Pd(0)/Pd(II) Sources 5

4.4 Table S1: Ligand Screen 5

4.5 Table S2: Initial Scoping Reactions 6

4.6 Tables S3-4: Screening of Pd Source and Substrate Scope 6

4.7 Table S5: Influence of Alkene Concentration 8

4.8 Tables S6-8: Catalyst Pre-activation 9

4.9 Figure S4: Influence of Catalyst Pre-activation on Chromatograms 10

4.10 Table S9: Substrate Scope After Implementation of Catalyst Pre-activation 11

4.11 Tables S10-11: Co-solvent Screen and Substrate Scope 11

4.12 Tables S12-13: Influence of Temperature and Additives 13

4.13 Figure S5: Scheme for Construction of **HP-2** 14

4.14 Figure S6: Scheme for Construction of **HP-3** 14

5 Procedures **15**

5.1 Off-DNA 15

5.1.1 2-(2-(2-(2-hydroxyethoxy)ethoxy)ethoxy)ethyl 4-methylbenzenesulfonate 15

5.1.2 2-(2-(2-(2-(4-iodophenoxy)ethoxy)ethoxy)ethoxy)ethan-1-ol 15

5.1.3 Ethyl 14-(4-iodophenoxy)-3,6,9,12-tetraoxatetradecanoate 16
5.1.4 14-(4-iodophenoxy)-3,6,9,12-tetraoxatetradecanoic acid 16
5.2 On-DNA 17
5.2.1 General Procedure for Ethanol Precipitation of DNA Conjugates 17
5.2.2 General Procedure for MMT-Deprotection 17
5.2.3 General Procedure for Synthesis of Headpieces 17
5.2.4 General Procedure for on-DNA Amide Coupling 18
5.2.5 General Procedure for Optimised on-DNA Heck Reaction 19

6 Chromatograms and Spectra **29**
6.1 NMR Spectra 29
6.1.1 2-(2-(2-(2-hydroxyethoxy)ethoxy)ethoxy)ethyl 4-methylbenzenesulfonate 30
6.1.2 2-(2-(2-(2-(4-iodophenoxy)ethoxy)ethoxy)ethoxy)ethan-1-ol 31
6.1.3 Ethyl 14-(4-iodophenoxy)-3,6,9,12-tetraoxatetradecanoate 32
6.1.4 14-(4-iodophenoxy)-3,6,9,12-tetraoxatetradecanoic acid 33
6.2 Oligonucleotide Chomatograms and Mass Spectra 34

1 Solvents and Reagents

Chemicals were purchased from Fluorochem, Sigma-Aldrich and TCI and used without further purification. Fmoc-NH-PEG_4-CO_2H linker was purchased from Key Organics. TPGS-750-M was purchased from Sigma-Aldrich; concentrations of surfactant in water are quoted as percentages (by weight) as used by the supplier. All water used with DNA substrates was nuclease-free water purchased from ThermoFisher. DNA was purchased from Sigma-Aldrich as either solid supported crude material or supplied as single strands after desalting.

2 Analytical Techniques

FTIR spectra were measured using an Agilent Cary 630 FTIR as a neat sample. UV spectra were recorded on a Hitachi U-2800A spectrophotometer and were performed in ethanol. LC-MS analyses were conducted using a Waters Acquity UPLC system with PDA and ELSD. When a 2 min gradient was used, the sample was eluted on an Acquity UPLC BEH C18 column (1.7μm, 2.1 x 50 mm), with a flow rate of 0.6 mL/min using 5-95% 0.1% HCOOH in MeCN. HRMS analysis was conducted using an Agilent 6550 iFunnel QTOF LC-MS in either positive or negative mode with an Agilent 1260 Infinity UPLC system. The sample was eluted on an Acquity UPLC BEH C18 column (1.7μm, 2.1 x 50 mm), with a flow rate of 0.7 mL/min at a gradient of 1.2 min 5-95% 0.1% HCOOH in MeCN with 0.1% aq. HCOOH. Exact masses were calculated using ChemDraw Professional 15.0.

^{1}H NMR spectra were obtained using a Bruker Avance III 500 spectrometer using a frequency of 500 MHz. ^{13}C NMR spectra were acquired using the Bruker Avance III 500 spectrometer operating at a frequency of 126 MHz. The abbreviations for spin multiplicity are as follows: s = singlet; d = doublet; t = triplet; q = quartet; quin = quintet; sept = septet and m = multiplet. Combinations of these abbreviations are employed to describe more complex splitting patterns (e.g. dd = doublet of doublets) and where broadening of the peak is observed, spin multiplicity is accompanied by the prefix br = broad. Spectra were obtained as $CDCl_3$ solutions.

DNA mass spectrometry was conducted on an Agilent 6550 iFunnel QTOF in negative mode, using a standard 3200 m/z maximum and a 2 GHz extended dyanmic range; drying gas temperature was set to 260 ^{o}C at 12 L/min, sheath gas temperature was set to 400 ^{o}C at 12 L/min, nebulizer at 45 psig, VCap voltage of 4000 V and nozzle volatge of 2000 V.

Oligonucleotide LC was conducted using an Agilent 1260 Infinity 2 on an Agilent Advancedbio oligonucleotides column, 2.1 x 150 mm, with a gradient of 0.4 mL/min from 10% MeOH to 40% MeOH over 8 mins against a 200 mM HFIP:8 mM TEA aqueous buffer solution; a 3 min flush at 95% MeOH preceded each run. Alternatively an Agilent Advancebio oligonucleotide column, 2.1 x 100 mm was employed, with a gradient of 0.8 mL/min from 10% MeOH to 50% MeOH over 4 mins against a 50 mM HFIP:15 mM DIPEA aqueous buffer solution. A 1 min flush at 95% MeOH preceded each run. Analysis of data was conducted with Agilent Qualitative Analysis version 7.

DNA concentrations were calculated using a NanoDrop^{TM}One/OneC Microvolume UV-Vis Spectrophotometer, pipetting 1 μL onto the loading plate.

3 Chromatography and Equipment

TLC utilised to monitor reaction progress was conducted on plates pre-coated with silica gel (Merck 60F254). The eluent was as stated (where this consisted of more than one solvent the ratio is stated as volume:volume) and visualisation was by short wave (254 nm) ultraviolet light. Flash column chromatography purifications were carried out using Biotage SP4 and Isolera automated flash sustems

with UV montioring at 278 nm and collection at 254 nm. Grave Resolve pre-packed flash cartiridges were used for normal phase separations.

Preparative HPLC purification was performed onan Agilent 1260 Infinity system with a Phenomenex Clarity 5μm Oligo-RP column, 21.2 x 250 mm using a gradient of 20% MeOH to 60% MeOH over 11 mins against a 200 mM HFIP:15 mM DIPEA aqueous buffer solution. Fractions were analysed at 260 nm wavelength.

4 Supplementary Figures and Tables

4.1 Figure S1: Scheme for Linker Synthesis

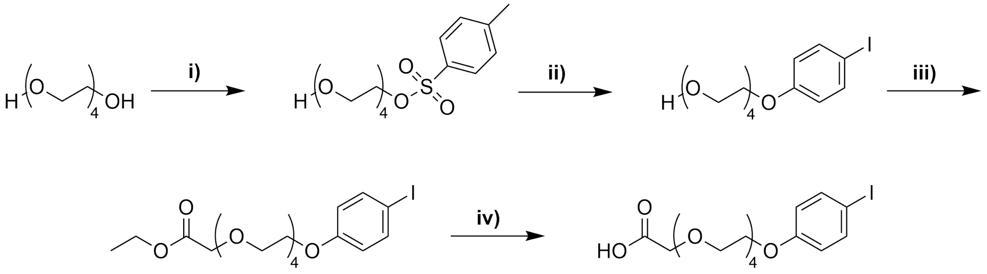

Conditions: *i)* TsCl, NEt_3, DCM, 98%; **ii)** 4-iodophenol, K_2CO_3, DMF, 50 ^{o}C, 81%; **iii)** ethyl diazoacetate, $Rh_2(OAc)_4$, DCM, 33%; **iv)** KOH, EtOH, 48%

4.2 Figure S2: Scheme for Construction of HP-1

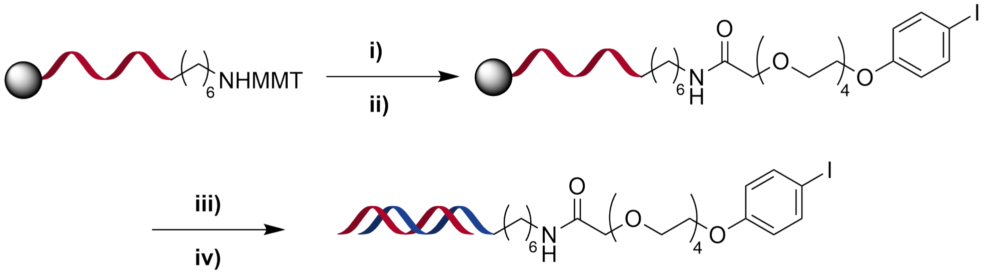

HP-1

Conditions: **i)** 3% TCA/DCM; **ii)** 14-(4-iodophenoxy)-3,6,9,12-tetraoxatetradecanoic acid, HATU, DIPEA, DMF; **iii)** aq. $NH_3/MeNH_2$; **iv)** complementary strand, 80 ^{o}C

4.3 Figure S3: Incubation of HP-1 with Pd(0)/Pd(II) Sources

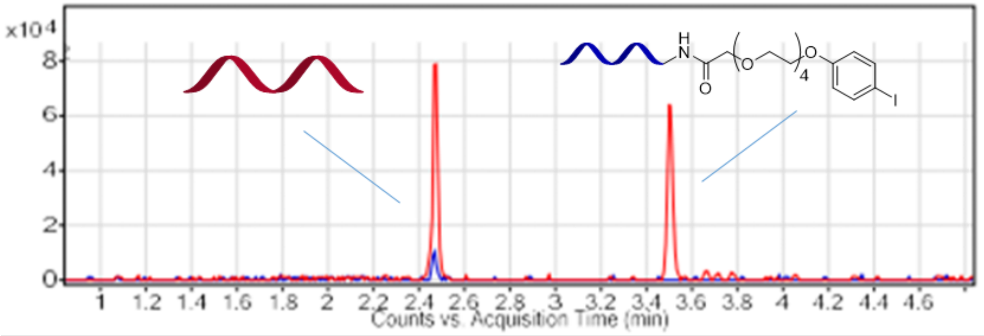

Comparison of chromatograms obtained following incubation of **HP-1** in the presence of Pd(0)/Pd(II) sources. Conditions: Red: 1 nmol DNA, 7.33 mM $Pd_2(dba)_3$, 7.33 mM dtbpf, 5.3 mM K_3PO_4, 2% TPGS-750-M (30 μL)/15% THF (4.5 μL), 60 ^oC, 1 hr; Blue: 1 nmol DNA, 7.33 mM $Pd(dtbpf)Cl_2$, 5.3 mM K_3PO_4, 2% TPGS-750-M (30 μL)/15% THF (4.5 μL), 60 ^oC, 1 hr

4.4 Table S1: Ligand Screen

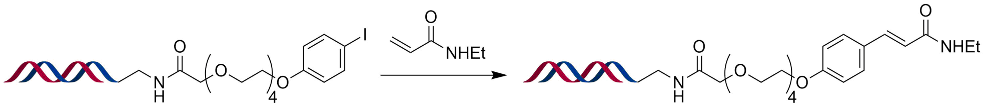

Entry	Catalyst	Ligand	Product (%)	SM (%)
1	$Pd_2(dba)_3$	CataCXium A	11	89
2	$Pd_2(dba)_3$	$P(o\text{-}tol)_3$	7	93
3	$Pd_2(dba)_3$	QPhos	16	84
4	$Pd_2(dba)_3$	XPhos	37	63
5	$Pd_2(dba)_3$	JohnPhos	29	71
6	$Pd[P(o\text{-}tol)_3]_2Cl_2$	N/A	0	100
7	$Pd[P(^tBu)_3]_2$	N/A	27	73

General reaction conditions: 2 nmol DNA, 7.33 mM "Pd", 14.66 mM "L", 5.3 mM K_3PO_4, 500 mM N-ethylacrylamide, 2% TPGS-750-M (30 μL)/15% THF (4.5 μL), 60 ^oC, 1 hr.

4.5 Table S2: Initial Scoping Reactions

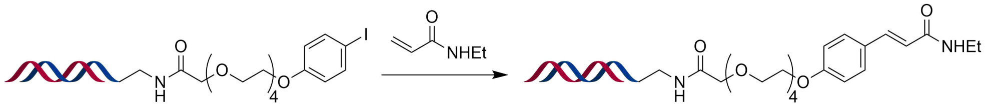

Entry	Modification	Product (%)	SM (%)	Dehal (%)
1	250 mM N-ethylacrylamide	41	52	1
2	7.33 mM $Pd_2(dba)_3$/ 29.32 mM XPhos	38	61	1
3	53 mM K_3PO_4	45	53	2
4	530 mM K_3PO_4	11	89	0
5	3.3% TPGS-750-M	41	58	1
6	Combined factors[a], 2% TPGS-750-M	29	71	0
7	Combined factors[a], 3.3% TPGS-750-M	23	77	0
8	125 mM N-ethylacrylamide[b]	49	50	1

General reaction conditions: 2 nmol DNA, 3.67 mM $Pd_2(dba)_3$, 14.66 mM XPhos, 5.3 mM K_3PO_4, 500 mM N-ethylacrylamide, 2% TPGS-750-M (30 μL)/15% THF (4.5 μL), 60 ^{o}C, 1 hr unless stated differently under modification column. [a]"Combined factors" refers to the combination of those individually determined to show improvement to reaction progression, i.e. 250 mM N-ethylacrylamide, 53 mM K_3PO_4 and 7.33 mM $Pd_2(dba)_3$/ 29.32 mM XPhos; [b]Reaction performed with 53 mM K_3PO_4.

4.6 Tables S3-4: Screening of Pd Source and Substrate Scope

Table S3: Pd Source Screen

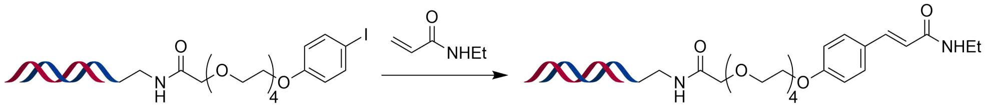

Entry	Catalyst	Ligand	Product (%)	SM (%)	Dehal (%)
1	$PdCl_2$	XPhos	86	10	4
2	$Pd(OAc)_2$	XPhos	91	7	2
3	$[(cin)PdCl]_2$	XPhos	95	0	5
4	XPhosPdG3	N/A	100	0	0

General reaction conditions: 2 nmol DNA, 7.33 mM "Pd", 14.66 mM XPhos, 53 mM K_3PO_4, 125 mM N-ethylacrylamide, 2% TPGS-750-M (30 μL)/15% THF (4.5 μL), 60 ^{o}C, 1 hr unless stated otherwise. Entry 4 relating to XPhosPdG3 contained no additional ligand resulting in overall reaction concentrations of 7.33 mM "Pd" and 7.33 mM XPhos.

Table S4: Substrate Scope with XPhosPdG3

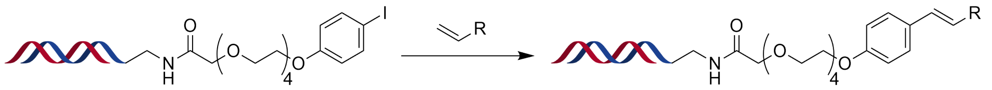

Entry	R	Product (%)	SM (%)	Dehal (%)
1	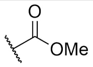	65[a]	0	35
2	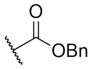	52[b]	0	48
3	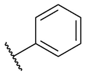	32	0	68
4	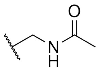	15	0	85
5	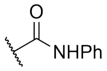	85	0	15
6	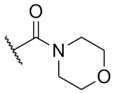	100	0	0

General reaction conditions: 2 nmol DNA, 7.33 mM XPhosPdG3, 53 mM K_3PO_4, 125 mM alkene, 2% TPGS-750-M (30 μL)/15% THF (4.5 μL), 60 ^oC, 1 hr; [a]20% hydrolysed; [b]33% hydrolysed.

4.7 Table S5: Influence of Alkene Concentration

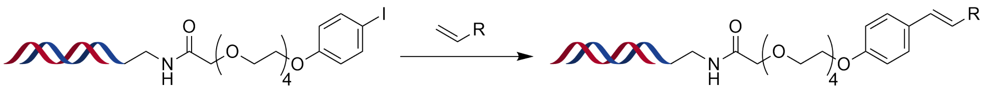

Entry	Pd/L	R	[Alkene] (mM)	Product (%)	SM (%)	Dehal (%)
1	XPhosPdG3	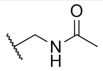	125	30	0	70
2	XPhosPdG3	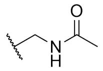	250	40	0	60
3	XPhosPdG3	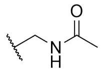	500	45	0	55
4	$[(cin)PdCl]_2$/XPhos	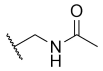	125	N.D.	N.D.	N.D.
5	$[(cin)PdCl]_2$/XPhos	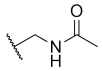	250	70	0	30
6	$[(cin)PdCl]_2$/XPhos	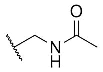	500	60	0	40
7	$[(cin)PdCl]_2$/XPhos	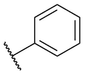	125	53	0	47
8	$[(cin)PdCl]_2$/XPhos	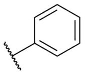	250	67	0	33
9	$[(cin)PdCl]_2$/XPhos	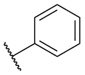	500	57	0	43

General reaction conditions: 2 nmol DNA, 7.33 mM ”Pd”, 14.66 mM ”L”, 53 mM K_3PO_4, 2% TPGS-750-M (30 μL)/15% THF (4.5 μL), 60 °C, 1 hr. Entries relating to XPhosPdG3 contained no additional ligand resulting in overall reaction concentrations of 7.33 mM ”Pd” and 7.33 mM XPhos.

4.8 Tables S6-8: Catalyst Pre-activation

Table S6: Pre-activation Prior to Transfer

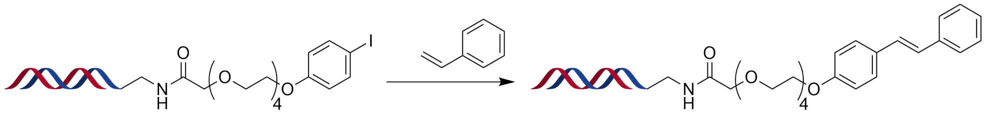

Entry	Pd/L	K_3PO_4 (mM)	RS:CS[a]	Product (%)	SM (%)	Dehal (%)
1	$[(cin)PdCl]_2$/XPhos	53	1.1	9	89	2
2	XPhosPdG3	53	1.1	0	100	0
3	$[(cin)PdCl]_2$/XPhos	530	1.0	11	86	14
4	XPhosPdG3	530	1.0	23	74	3

General reaction conditions: 2 nmol DNA, 7.33 mM "Pd", 14.66 mM "L", 250 mM styrene, 2% TPGS-750-M (30 μL)/15% THF (4.5 μL), 60 ^oC, 1 hr. Entries relating to XPhosPdG3 contained no additional ligand resulting in overall reaction concentrations of 7.33 mM "Pd" and 7.33 mM XPhos; [a]Ratio of reactive and complementary strands expressed as reactive strand area divided by complementary strand area.

"Pd", "L" and K_3PO_4 in THF/H_2O were mixed at 50 ^oC in an eppendorf for 10 minutes prior to being added to the reaction.

Table S7: Pre-activation in Reaction Vial at 50 ^{o}C

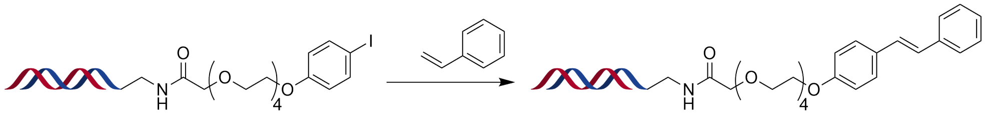

Entry	Pd/L	K_3PO_4 (mM)	RS:CS[a]	Product (%)	SM (%)	Dehal (%)
1	$[(cin)PdCl]_2$/XPhos	53	1.1	23	74	3
2	XPhosPdG3	53	1.1	8	91	1
3	$[(cin)PdCl]_2$/XPhos	530	1.0	42	50	8
4	XPhosPdG3	530	1.0	26	71	3

General reaction conditions: 2 nmol DNA, 7.33 mM "Pd", 14.66 mM "L", 250 mM styrene, 2% TPGS-750-M (30 μL)/15% THF (4.5 μL), 60 ^oC, 1 hr. Entries relating to XPhosPdG3 contained no additional ligand resulting in overall reaction concentrations of 7.33 mM "Pd" and 7.33 mM XPhos; [a]Ratio of reactive and complementary strands expressed as reactive strand area divided by complementary strand area.

"Pd", "L" and K_3PO_4 in THF/H_2O were heated at 50 ^oC in the reaction vial for 10 minutes prior to addition of remaining reaction components.

Table S8: Pre-activation in Vial at Room Temperature

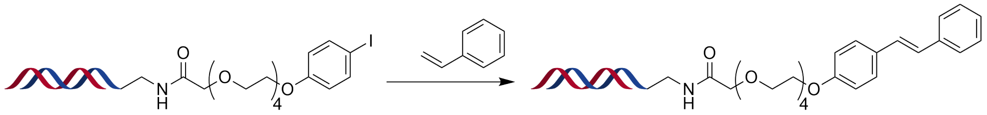

Entry	Pd/L	Time^a (min)	RS:CS^b	Product (%)	SM (%)	Dehal (%)
1	$[(cin)PdCl]_2$/XPhos	10	1.0	98	0	2
2	XPhosPdG3	10	1.1	26	72	2
3	$[(cin)PdCl]_2$/XPhos	0	1.0	83	9	8
4	XPhosPdG3	0	1.0	30	68	2

General reaction conditions: 2 nmol DNA, 7.33 mM ”Pd”, 14.66 mM ”L”, 250 mM styrene, 2% TPGS-750-M (30 μL)/15% THF (4.5 μL), 60 ^{o}C, 1 hr. Entries relating to XPhosPdG3 contained no additional ligand resulting in overall reaction concentrations of 7.33 mM ”Pd” and 7.33 mM XPhos; ^{a}”Time” refers to length of time catalyst pre-activation was performed for; ^{b}Ratio of reactive and complementary strands expressed as reactive strand area divided by complementary strand area.

”Pd”, ”L” and K_3PO_4 in THF/H_2O were stood at room temperature in the reaction vial for the specified number of minutes prior to addition of remaining reaction components.

4.9 Figure S4: Influence of Catalyst Pre-activation on Chromatograms

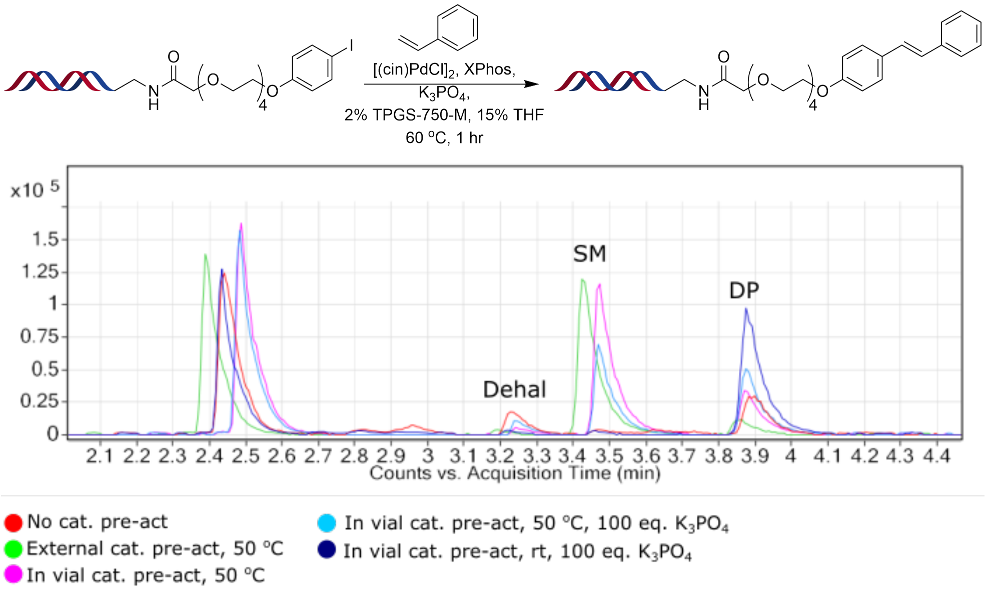

Overlay of differences in chromatograms obtained utilising alternative modes of catalyst pre-activation with $[(cin)PdCl]_2$/XPhos. Both reaction progression and DNA integrity were improved through the incorporation and optimisation of the procedure. DP = desired product.

4.10 Table S9: Substrate Scope After Implementation of Catalyst Pre-activation

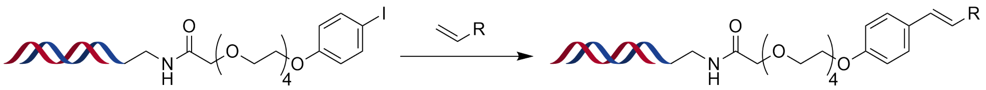

Entry	R	Product (%)	SM (%)	Dehal (%)
1	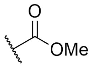	94[a]	2	4
2	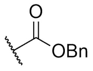	13[a]	85	2
3	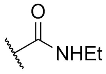	88	12	0
4	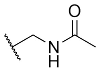	61	39	0
5	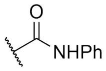	2	98	0
6	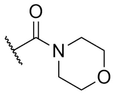	72	28	0

General reaction conditions: 2 nmol DNA, 3.67 mM $[(cin)PdCl]_2$, 14.66 mM XPhos, 530 mM K_3PO_4, 250 mM alkene, 2% TPGS-750-M (30 μL)/15% THF (4.5 μL), 60 ^{o}C, 1 hr; [a]present as hydrolysed product.

$[(cin)PdCl]_2$, XPhos and K_3PO_4 in THF/H_2O were stood at room temperature in the reaction vial for 10 minutes prior to addition of remaining reaction components.

4.11 Tables S10-11: Co-solvent Screen and Substrate Scope

Table S10: Co-solvent Screen

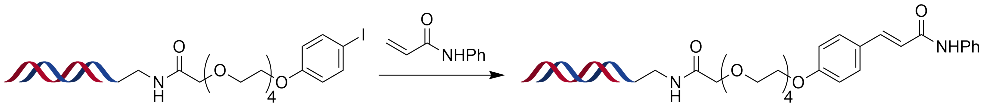

Entry	Co-solvent	Product (%)	SM (%)	Dehal (%)	Other (%)
1	THF	5	95	0	0
2	DMF	76	0	24	0
3	DMPU	57	0	0	43[a]
4	NMP	63	0	0	37[a]

General reaction conditions: 2 nmol DNA, 3.67 mM $[(cin)PdCl]_2$, 14.66 mM XPhos, 530 mM K_3PO_4, 250 mM N-phenylacrylamide, 2% TPGS-750-M (30 μL)/15% co-solvent (4.5 μL), 60 ^{o}C, 1 hr; [a]Variety of unidentified side products, 2-13%.

[(cin)PdCl]$_2$, XPhos and K_3PO_4 in co-solvent/H_2O were stood at room temperature in the reaction vial for 10 minutes prior to addition of remaining reaction components.

Table S11: Substrate Scope Employing DMF as Co-solvent

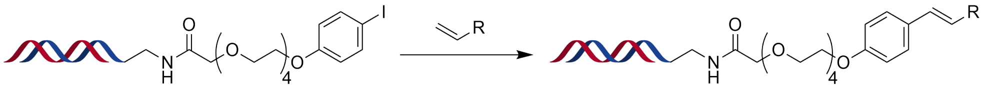

Entry	R	Product (%)	SM (%)	Dehal (%)	Other (%)
1	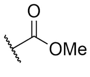	84[a]	10	6	0
2	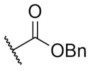	79[b]	2	19	0
3	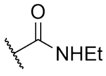	100	0	0	0
4	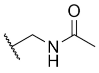	82	0	0	18
5	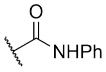	87	0	13	0
6	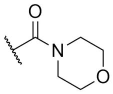	100	0	0	0
7	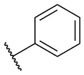	82	12	6	0

General reaction conditions: 2 nmol DNA, 3.67 mM [(cin)PdCl]$_2$, 14.66 mM XPhos, 530 mM K_3PO_4, 250 mM alkene, 2% TPGS-750-M (30 μL)/15% DMF (4.5 μL), 60 ^{o}C, 1 hr; [a]Present as the hydrolysed product; [b]67% present as the hydrolysed product; [c]Due to the solid nature of N-phenylacrylamide, this reagent was added first in this instance and as such was present during the catalyst pre-activation procedure.

[(cin)PdCl]$_2$, XPhos and K_3PO_4 in DMF/H_2O were stood at room temperature in the reaction vial for 10 minutes prior to addition of remaining reaction components.

4.12 Tables S12-13: Influence of Temperature and Additives

Table S12: Temperature Effects

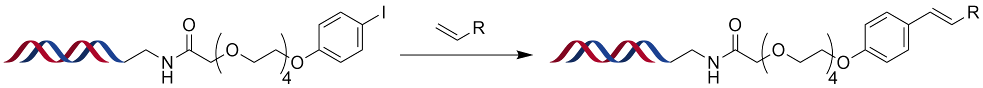

Entry	R	Product (%)	SM (%)	Dehal (%)
1	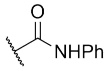	95	2	3
2	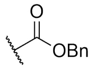	96[a]	1	3

General reaction conditions: 2 nmol DNA, 3.67 mM $[(cin)PdCl]_2$, 14.66 mM XPhos, 530 mM K_3PO_4, 250 mM alkene, 2% TPGS-750-M (30 μL)/15% DMF (4.5 μL), 50 ^{o}C, 2 hr; [a]31% present as the hydrolysed product.

$[(cin)PdCl]_2$, XPhos and K_3PO_4 in DMF/H_2O were stood at room temperature in the reaction vial for 10 minutes prior to addition of remaining reaction components.

Table S13: Additive Effects

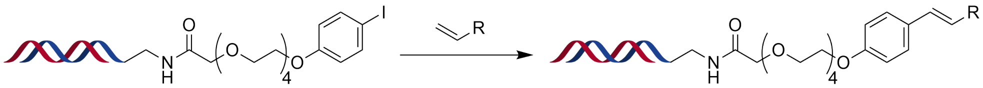

Entry	R	[Alkene] (mM)	L:Pd eq.	Additives	Product (%)	SM (%)	Dehal (%)
1	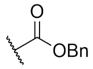	250	1	N/A	98[a]	0	2
2	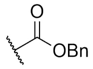	250	2	N/A	100[a]	0	0
3	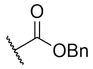	250	4	N/A	77[a]	23	0
4	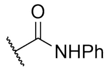	250	2	NaCl	100	0	0
5	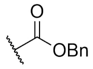	250	2	NaCl	95[a]	1	4
6	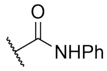	375	2	N/A	86	0	14
7	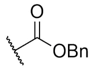	375	2	N/A	98[a]	0	2

General reaction conditions: 2 nmol DNA, 3.67 mM $[(cin)PdCl]_2$, 14.66 mM XPhos, 530 mM K_3PO_4, 250 mM alkene, 2% TPGS-750-M (30 μL)/15% DMF (4.5 μL), 60 ^{o}C, 1 hr unless indicated otherwise;

[a]Present as a mixture of both ester and the hydrolysed product.

$[(cin)PdCl]_2$, XPhos and K_3PO_4 in DMF/H_2O were stood at room temperature in the reaction vial for 10 minutes prior to addition of remaining reaction components.

4.13 Figure S5: Scheme for Construction of HP-2

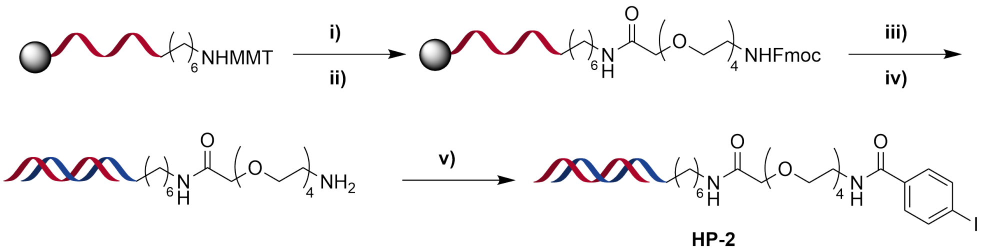

HP-2

Conditions: **i)** 3% TCA/DCM; **ii)** 1-(9H-fluoren-9-yl)-3-oxo-2,7,10, 13,16-pentaoxa-4-azaoctadecan-18-oic acid, HATU, DIPEA, DMF; **iii)** aq. NH_3/$MeNH_2$; **iv)** complementary strand, 80 ^{o}C; **v)** 4-iodobenzoic acid, HATU, 2,6-lutidine, 3.5% TPGS-750-M, 45 ^{o}C

4.14 Figure S6: Scheme for Construction of HP-3

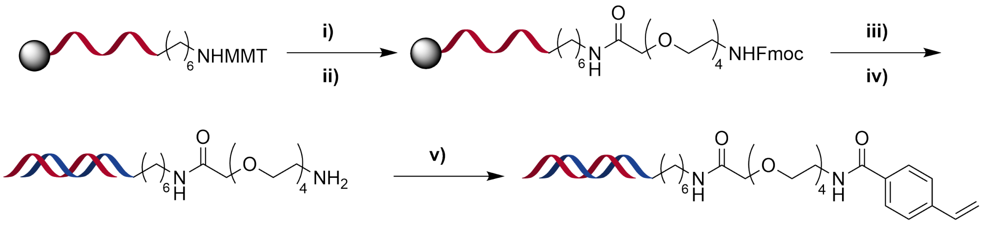

HP-3

Conditions: **i)** 3% TCA/DCM; **ii)** 1-(9H-fluoren-9-yl)-3-oxo-2,7,10, 13,16-pentaoxa-4-azaoctadecan-18-oic acid, HATU, DIPEA, DMF; **iii)** aq. NH_3/$MeNH_2$; **iv)** complementary strand, 80 ^{o}C; **v)** 4-vinylbenzoic acid, HATU, 2,6-lutidine, 3.5% TPGS-750-M, 45 ^{o}C

5 Procedures

5.1 Off-DNA

5.1.1 2-(2-(2-(2-hydroxyethoxy)ethoxy)ethoxy)ethyl 4-methylbenzenesulfonate

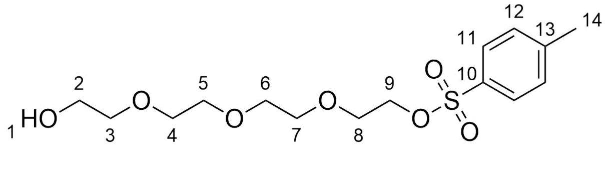

Under an N_2 atmosphere, tetraethylene glycol (3880 mg, 20 mmol) and NEt_3 (0.56 mL, 4 mmol) were suspended in DCM (50 mL) and the solution was cooled to 0 ^{o}C. TsCl (380 mg, 2 mmol) was added in portions and the resulting reaction mixture was allowed to warm to room temperature and left to stir for 16 hours. The solution was diluted with DCM (50 mL) and washed with H_2O (3 x 50 mL), sat. $NaHCO_3$ (2 x 50 mL) and brine (50 mL). The organics were then dried over Na_2SO_4, filtered and concentrated to yield the product as a pale yellow oil (687 mg, 1.96 mmol, 98%). No further purification was required.

R_f: 0.13 (1:19 MeOH/DCM); UV λ_{max} (EtOH/nm) 222.4; IR ν_{max} (cm^{-1}) 3428 (s, br, O-H alcohol), 2866 (m, C-H alkane), 2090 (w, aromatic overtones), 1350 (s, S=O sulfonate), 1095 (s, C-O ether); ^{1}H NMR (500 MHz, $CDCl_3$) δ 7.79 (d, J= 8.1 Hz, 2H, H11), 7.33 (d, J= 8.1 Hz, 2H, H12), 4.17-4.12 (m, 2H, H9), 3.73-3.66 (m, 4H, PEG-H), 3.66-3.64 (m, 2H, PEG-H), 3.64-3.60 (m, 2H, PEG-H), 3.60-3.54 (m, 6H, PEG-H), 2.44 (s, 3H, H14); ^{13}C NMR: (126 MHz, $CDCl_3$) δ 144.92 (C10), 133.11 (C13), 129.94 (C12), 128.09 (C11), 72.56 (PEG-C), 70.86 (PEG-C), 70.78 (PEG-C), 70.60 (PEG-C), 70.47 (PEG-C), 69.37 (PEG-C), 68.83 (PEG-C), 61.86 (PEG-C), 21.76 (C14); LRMS(ES^{+}) m/z calculated for $C_{15}H_{24}O_7S$: 349.1 ([M+H]$^{+}$); observed 349.2 [M+H]$^{+}$.

5.1.2 2-(2-(2-(2-(4-iodophenoxy)ethoxy)ethoxy)ethoxy)ethan-1-ol

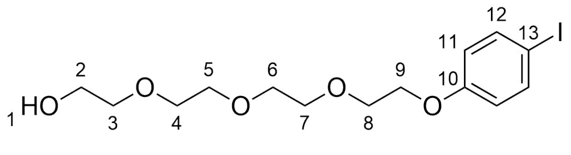

2-(2-(2-(2-hydroxyethoxy)ethoxy)ethoxy)ethyl 4-methylbenzenesulfonate (531 mg, 1.52 mmol) and K_2CO_3 (773 mg, 5.60 mmol) were suspended in DMF (10 mL) under N_2. 4-Iodophenol (308 mg, 1.40 mmol) was added to the solution and the reaction mixture was stirred at 60 ^{o}C for 16 hours. H_2O (40 mL) was added to the reaction and extraction was performed with DCM (3 x 30 mL). Organic fractions were combined, washed with 10% aq. citric acid (2 x 30 mL) and brine (30 mL), dried over Na_2SO_4, filtered and concentrated. The product was obtained as a colourless oil (465 mg, 1.13 mmol, 81%).

R_f: 0.42 (1:19 MeOH/DCM); UV λ_{max} (EtOH/nm) 281.0, 233.4; IR ν_{max} (cm^{-1}) 3429 (s, br, O-H alcohol), 2868 (m, C-H alkane), 1874 (w, aromatic overtones); ^{1}H NMR (500 MHz, $CDCl_3$) δ 7.52 (d, J= 8.6 Hz, 2H, H11), 6.68 (d, J= 8.6 Hz, 2H, H12), 4.11-4.06 (m, 2H, H9), 3.87-3.76 (m, 2H, H8), 3.73-3.60 (m, 12H, PEG-H); ^{13}C NMR: (126 MHz, $CDCl_3$) δ 158.90 (C10), 138.49 (C12), 117.37 (C11), 83.32 (C13), 71.45 (PEG-C), 71.15 (PEG-C), 70.89 (PEG-C), 70.72 (PEG-C), 70.58 (PEG-C), 69.90 (PEG-C), 69.27 (PEG-C), 67.70 (PEG-C); LRMS(ES^{+}) m/z calculated for $C_{15}H_{21}IO_5$: 397.1 ([M+H]$^{+}$); observed 397.2 [M+H]$^{+}$

5.1.3 Ethyl 14-(4-iodophenoxy)-3,6,9,12-tetraoxatetradecanoate

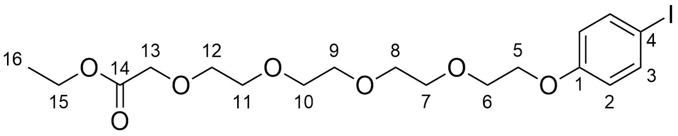

Under an N_2 atmosphere, 2-(2-(2-(2-(4-iodophenoxy)ethoxy)ethoxy)ethoxy)ethano-1-ol (465 mg, 1.13 mmol) and $Rh_2(OAc)_4$ (4 mg, 9 μmol) were suspended in DCM (20 mL) and cooled to 0 ^{o}C. Ethyl diazoacetate (0.13 mL, 1.24 mmol) was added slowly and the reaction mixture was allowed to warm to room temperature and left to stir for 16 hours. The solution was then diluted with DCM (40 mL) and filtered through celite. The filtrate was washed with brine (20 mL), dried over Na_2SO_4, filtered and concentrated. Purification was performed via column chromatography (0-20% MeOH/DCM) to yield the product as a pale yellow oil (221 mg, 0.37 mmol, 33%).

R_f: 0.84 (1:19 MeOH/DCM); UV λ_{max} (EtOH/nm) 233.4; IR ν_{max} (cm^{-1}) 2869 (m, C-H alkane), 1882 (w, aromatic overtones), 1747 (s, C=O ester), 1099 (s, C-O ether); ^{1}H NMR (500 MHz, $CDCl_3$) δ 7.53 (d, J= 9.0 Hz, 2H, H2), 6.69 (d, J= 9.0 Hz, 2H, H3), 4.20 (q, J= 7.1 Hz, 2H, H15), 4.14 (s, 2H, H13), 4.10-4.05 (m, 2H, PEG-H), 3.85-3.82 (m, 2H, PEG-H), 3.72-3.62 (m, 12H, PEG-H), 1.27 (t, J = 7.1 Hz, 3H, H16); ^{13}C NMR: (126 MHz, $CDCl_3$) δ 170.59 (C14), 158.78 (C1), 138.29 (C3), 117.15 (C2), 83.10 (C4), 72.60 (PEG-C), 70.96 (PEG-C), 70.76 (PEG-C), 70.68 (PEG-C), 70.42 (PEG-C), 69.73 (PEG-C), 68.83 (PEG-C), 67.63 (PEG-C), 61.86 (PEG-C), 60.96 (C15), 14.24 (C16); LRMS(ES^+) m/z calculated for $C_{18}H_{28}IO_7$: 483.1 ($[M+H]^+$); observed 483.3 $[M+H]^+$.

5.1.4 14-(4-iodophenoxy)-3,6,9,12-tetraoxatetradecanoic acid

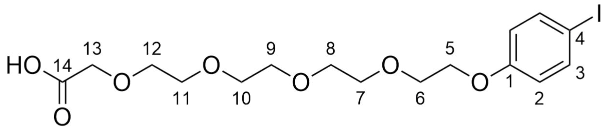

Ethyl 14-(4-iodophenoxy)-3,6,9,12-tetraoxatetradecanoate (221 mg, 0.46 mmol) was suspended in MeOH (10 mL); potassium hydroxide (51 mg, 0.92 mmol) was added to the solution and the reaction mixture was stirred overnight at room temperature. The reaction was diluted with H_2O (30 mL) and extracted with DCM (3 x 19 mL) to remove any unreacted starting material. The aqueous layer was then acidified to ca. pH 2 using 1 M HCl and extracted further with DCM (3 x 10 mL). Organic layers were combined, dried over Na_2SO_4, filtered and concentrated to yield the product as a yellow oil (99 mg, 0.23 mmol, 48%).

R_f: 0.16 (100% EtOAc); UV λ_{max} (EtOH/nm) 280.4, 233.4; IR ν_{max} (cm^{-1}) 3064 (s, br, 0-H carboxylic acid), 2870 (m, C-H alkane), 1876 (w, aromatic overtones), 1749 (s, C=O acid), 1097 (s, C-O ether); ^{1}H NMR (500 MHz, $CDCl_3$) δ 7.53 (d, J= 8.9 Hz, 2H, H2), 6.69 (d, J= 8.9 Hz, 2H, H3), 4.14 (s, 2H, H13), 4.11-4.05 (m, 2H, PEG-H), 3.91-3.78 (m, 2H, PEG-H), 3.77-3.69 (m, 4H, PEG-H), 3.69-3.63 (m, 8H, PEG-H); ^{13}C NMR: (126 MHz, $CDCl_3$) δ 171.95 (C14), 158.74 (C1), 138.31 (C3), 117.20 (C2), 83.12 (C4), 71.48 (PEG-C), 71.00 (PEG-C), 70.76 (PEG-C), 70.56 (PEG-C), 70.43 (PEG-C), 70.33 (PEG-C), 69.72 (PEG-C), 69.20 (PEG-C), 67.55 (PEG-C); LRMS(ES^-) m/z calculated for $C_{16}H_{22}IO_7$: 453.1 ($[M-H]^-$); observed 453.1 $[M+H]^-$.

5.2 On-DNA

5.2.1 General Procedure for Ethanol Precipitation of DNA Conjugates

To the reaction mixture was added 10% vol. NaCl (5 M in H_2O) and 3x vol. cold abs. EtOH; the reaction mixture was then stored at -20 ^oC for 16 hours. The sample was centrifuged at 13400 rpm for 10 minutes, the supernatant discarded and the residual pellet washed with 70% cold EtOH/H_2O before being centrifuged for a further 10 minutes at 13400 rpm. The supernatant was again discarded and the pellet allowed to air dry before being resuspended in water as a 1 mM solution.

5.2.2 General Procedure for MMT-Deprotection

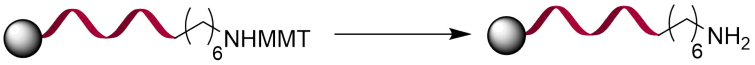

The average loading of single stranded DNA attached to solid support was found by cleavage from solid support using the below method and repeating three times. Nanodrop concentration of cleaved DNA showed that 103 mg yielded 2 μmol of DNA. The single-stranded DNA employed was a 14-mer (GTCTTGCCGAATTC) modified with a 5' MMT-amino C6 linker bound to solid support at the 3' end. Solid supported DNA (105 mg, ca. 2 μmol) was washed with 3% trichloroacetic acid in DCM (10 x 500 μL). A yellow colour indicated that the deprotection was in progress. Once this colour subsided the solid supported DNA was washed with DCM (3 x 500 μL) and left to air dry for 20 minutes.

5.2.3 General Procedure for Synthesis of Headpieces

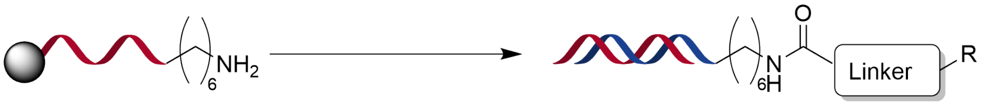

To a 1.5 mL microcentrifuge tube was added HATU (17 mg, 44 μmol), DIPEA (17 μL, 100 μmol) and DMF (1 mL). To this was added the required acid linker (40 μmol) and the mixture was shaken for 20 minutes at room temperature. Deprotected solid supported DNA (ca. 2 μmol) was added and the reaction was shaken overnight at room temperature. The mixture was then filtered and washed with DMF (3 x 500 μL), MeCN (3 x 500 μL), MeOH (3 x 500μL) and DCM (3 x 500 μL), before being allowed to air dry for 20 minutes.

40% methylamine in water (500μL) and 33% ammonia in water (500 μL) were mixed in a 1.5 mL microcentrifuge tube. The solid supported DNA was added and the mixture shaken overnight at room temperature. The mixture was then filtered and the beads washed with water (3 x 500 μL); the resulting filtrate was concentrated to ca. 0.5 mL using a Genevac at 40 ^oC. The crude product was then purified by HPLC, required fractions concentrated to dryness using a Genevac at 40 ^oC and resuspended in water (1 mL). The concentration of samples was then quantified by UV using a NanoDrop One by ThermoFisher. The usual amount was ca. 0.5-1 μmol of DNA after HPLC purification. The exact amount of the complimentary 14-mer (GAATTCGGCAAGAC) was then added added in water; the solution was heated to 80 ^oC for 1 hour then allowed to cool slowly. The double stranded DNA was concentrated to dryness using a Genevac at 40 ^oC and resuspended in water to form a 1 mM solution of the product.

DNA Conjugate HP-1

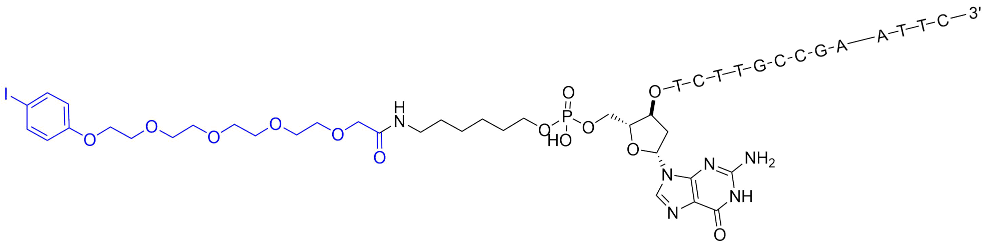

DNA conjugate **HP-1** was prepared according to the general procedure utilising 14-(4-iodophenoxy)-3,6,9,12-tetraoxatetradecanoic acid (numb , 18 mg).

HRMS (ESI): m/z calculated mass: 4842.8421; observed mass: 4842.8233

DNA Conjugate PEG4-NH$_2$

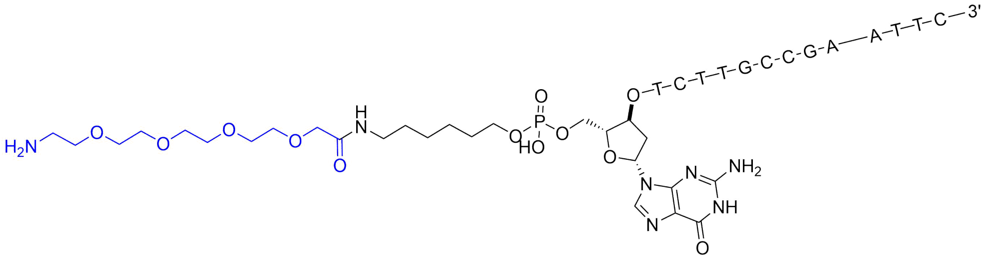

DNA conjugate **PEG4-NH$_2$** was prepared according to the general procedure utilising 1-(9H-fluoren-9-yl)-3-oxo-2,7,10, 13,16-pentaoxa-4-azaoctadecan-18-oic acid (19 mg).

HRMS (ESI): m/z calculated mass: 4639.9301; observed mass: 4639.9183

5.2.4 General Procedure for on-DNA Amide Coupling

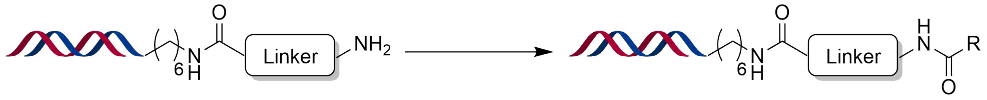

To a 50 μL glass insert containing the corresponding carboxylic acid (15.0 μmol) was added HATU (5.70 mg, 15.0 μmol), 5% TPGS-750-M (21 μL), 2,6-lutidine (6.92 μL, 60.0 μmol) and DNA (9 μL, 1 mM in H_2O). Vials were vortexed for 30 seconds, then heated at 45 ^oC for 16 hours in a Paradox^{TM} 96-well microphotoredox/optimisation plate. After cooling to room temperature, the reaction mixture was diluted with water (up to 200 μL) and washed with DCM (3 x 400 μL). Samples were then filtered through a hydrophilic PTFE filter and precipitated according to the general ethanol precipitation procedure.

DNA Conjugate HP-2

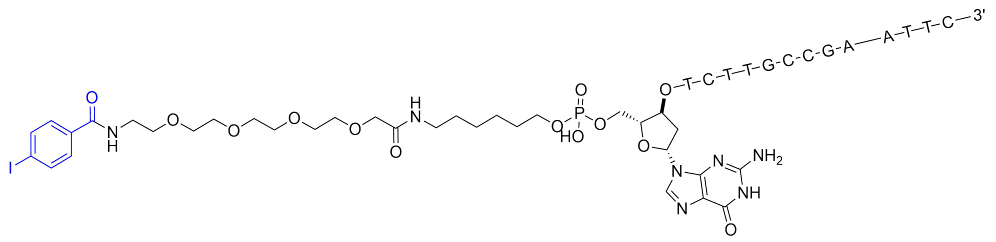

DNA conjugate **HP-2** was prepared according to the general procedure for on-DNA amide coupling employing 4-iodobenzoic acid (3.7 mg).

HRMS (ESI): m/z calculated mass: 4869.8530; observed mass: 4869.8476

DNA Conjugate HP-3

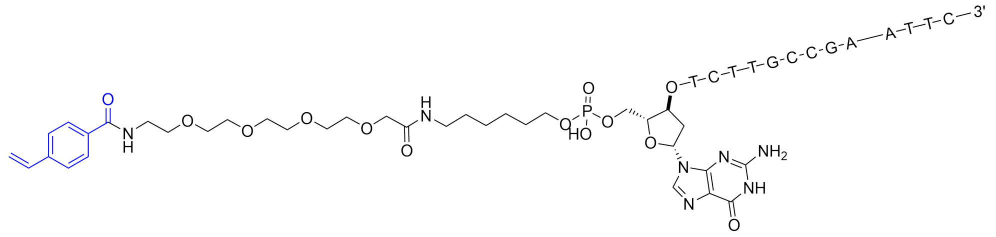

DNA conjugate **HP-3** was prepared according to the general procedure for on-DNA amide coupling employing 4-vinylbenzoic acid (2.2 mg).

HRMS (ESI): m/z calculated mass: 4769.9720; observed mass: 4769.9276

5.2.5 General Procedure for Optimised on-DNA Heck Reaction

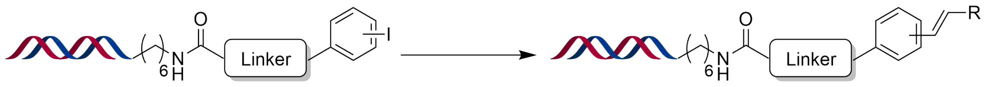

To a 50 μL glass insert for a Para-dox^{TM} 96-well microphotoredox plate was added K_3PO_4 (8 μL, 113.2 mg in 200 μL H_2O), [(cinnamyl)PdCl]$_2$ (2.25 μL, 5.1 mg in 200 μL DMF) and XPhos (2.25 μL, 9.5 mg in 100 μL DMF). Samples were vortexed for 30 seconds then allowed to stand at room temperature for a further 10 minutes before addition of 5% TPGS-750-M (12 μL), aq. NaCl (8 μL, 6.14 M), DNA (2 μL, 1 mM in H_2O) and alkene (11 μmol). Samples were vortexed for a further 30 seconds, then heated at 50 ^oC for 2 hours in a Paradox^{TM} 96-well microphotoredox/optimisation plate. Sodium diethyldithiocarbamate (6 μL, 1 M in H_2O) was then added to the reactions, which were heated at 60 ^oC for a further 30 minutes. The reaction mixtures were allowed to cool to room temperature then diluted with H_2O (up to 200 μL) and washed with DCM (3 x 400 μL). The organics were removed; the aqueous solutions were then filtered through a hydrophilic PTFE filter and subsequently analysed via mass spectrometry.

In instances where an alkenyl DNA conjugate was employed, aryl halide (11 μmol) was used in place of the alkene component.

Table of Results

Entry	Code	DNA-HP	Coupling Partner	Product (%)	SM (%)	Dehal (%)
1	1A	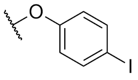	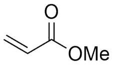	100[a]	0	0
2	1B	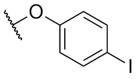	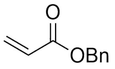	92[a]	6	2
3	1C	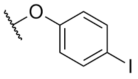	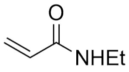	100	0	0
4	1D	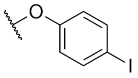	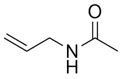	91	4	5
5	1E	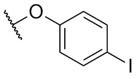	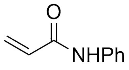	100	0	0
6	1F	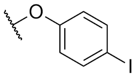	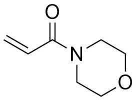	100	0	0
7	1G	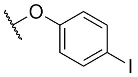	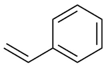	88	10	2
8	2A	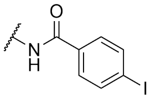	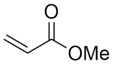	100[a]	0	0
9	2B	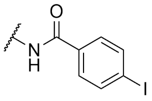	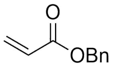	80[a]	3	17
10	2C	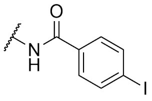	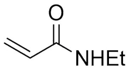	100	0	0
11	2D	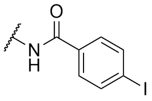	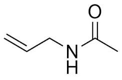	100	0	0
12	2E	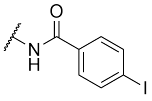	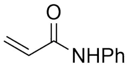	100	0	0
13	2F	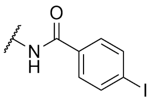	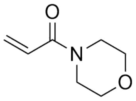	100	0	0
14	2G	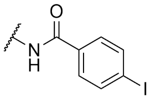	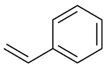	93	2	5

[a]Present as a mixture of the ester and the hydrolysed product

Entry	Code	DNA-HP	Coupling Partner	Product (%)	SM (%)	Dehal (%)
15	2H	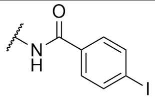	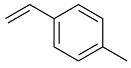	93	2	5
16	2I	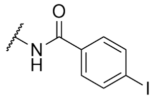	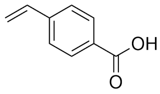	100	0	0
17	2J	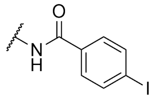	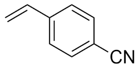	84	10	6
18	2K	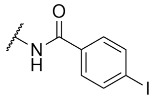	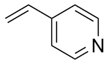	95	0	5
19	2L	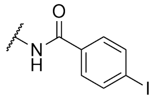	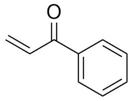	100	0	0
20	2M	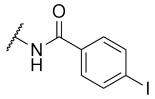	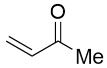	82	0	0
21	3N	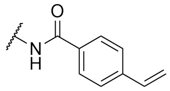	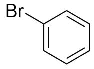	80	20	N/A
22	3O	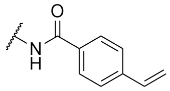	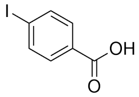	100[b]	0	0
23	3P	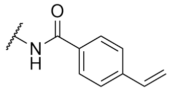	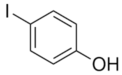	100[c]	0	N/A
24	3Q	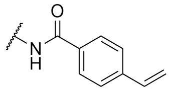	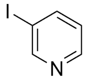	61	30	N/A

[b]15% present as double-substitution product; [c]91% present as double-substitution product.

DNA Conjugate 1A

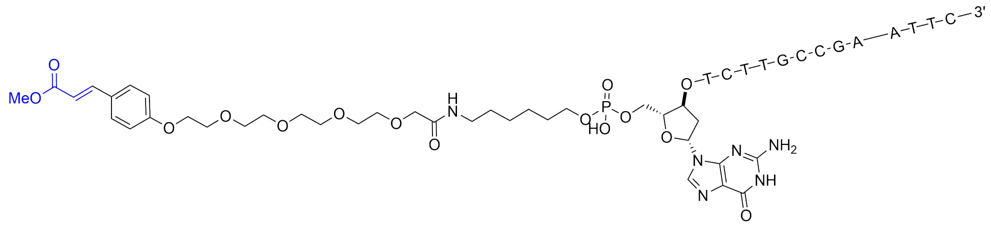

DNA conjugate **1A** was prepared according to the general procedure for optimised on-DNA Heck reaction employing Headpiece **HP-1** and methyl acrylate (1.02 μL).

HRMS (ESI): m/z calculated mass: 4800.9666 (acid: 4786.9509); observed mass: 4800.9272 (51%), 4786.9158 (49%)

DNA Conjugate 1B

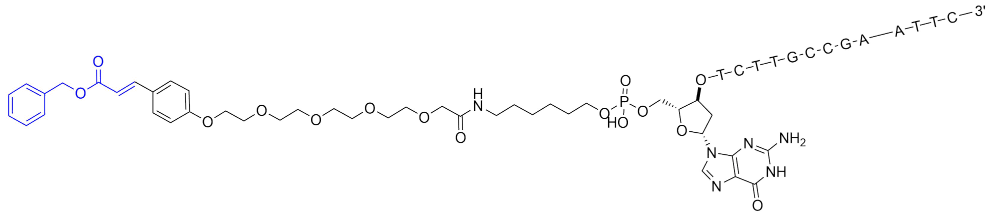

DNA conjugate **1B** was prepared according to the general procedure for optimised on-DNA Heck reaction employing Headpiece **HP-1** and benzyl acrylate (1.65 μL).

HRMS (ESI): m/z calculated mass: 4876.9979 (acid: 4786.9509); observed mass: 4876.9298 (49%), 4786.8529 (43%)

DNA Conjugate 1C

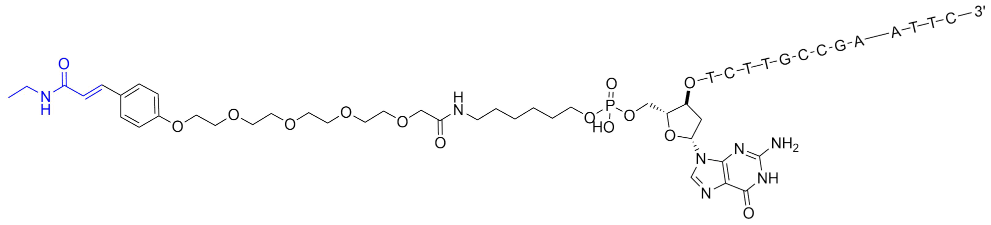

DNA conjugate **1C** was prepared according to the general procedure for optimised on-DNA Heck reaction employing Headpiece **HP-1** and N-ethylacrylamide (1.20 μL).

HRMS (ESI): m/z calculated mass: 4813.9982; observed mass: 4813.9550

DNA Conjugate 1D

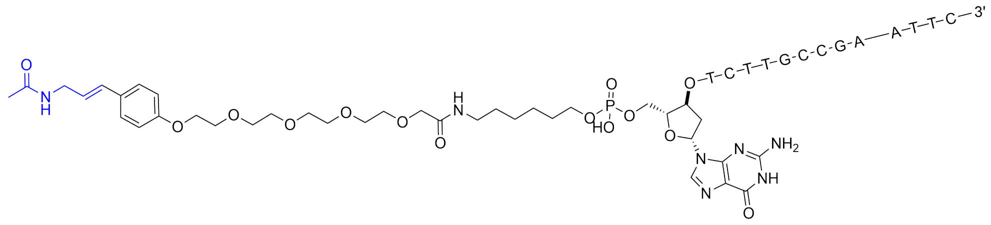

DNA conjugate **1D** was prepared according to the general procedure for optimised on-DNA Heck reaction employing Headpiece **HP-1** and N-allylacetamide (0.78 μL).

HRMS (ESI): m/z calculated mass: 4813.9982; observed mass 4813.9607

DNA Conjugate 1E

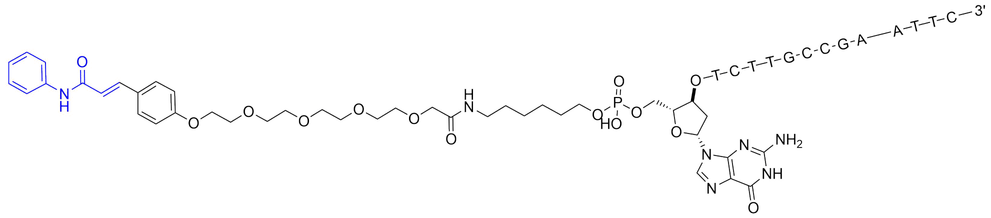

DNA conjugate **1E** was prepared according to the general procedure for optimised on-DNA Heck reaction employing Headpiece **HP-1** and N-phenylacrylamide (1.65 mg). In this instance due to the solid-phase nature of the alkene this was added to the reaction vial first and thus was present during the catalyst pre-activation step; no detrimental influence on reaction progression and/or DNA integrity was observed.

HRMS (ESI): m/z calculated mass: 4861.9982; observed mass: 4861.9578

DNA Conjugate 1F

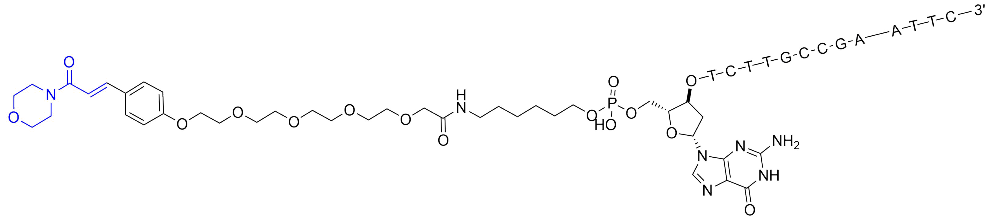

DNA conjugate **1F** was prepared according to the general procedure for optimised on-DNA Heck reaction employing Headpiece **HP-1** and N-acryloylmorpholine (1.41 μL).

HRMS (ESI): m/z calculated mass: 4856.0088; observed mass: 4855.9637

DNA Conjugate 1G

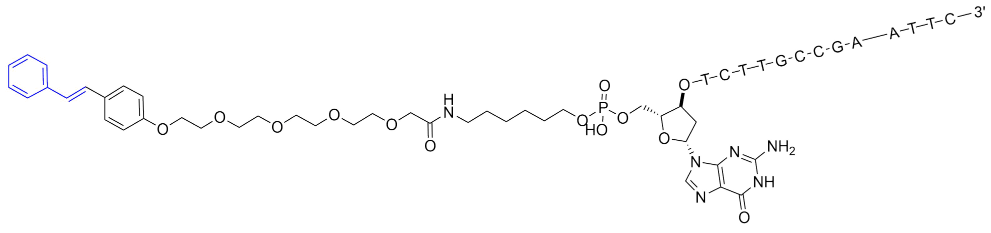

DNA conjugate **1G** was prepared according to the general procedure for optimised on-DNA Heck reaction employing Headpiece **HP-1** and styrene (1.29 μL).

HRMS (ESI): *m/z* calculated mass: 4818.9924; observed mass: 4818.9153

DNA Conjugate 2A

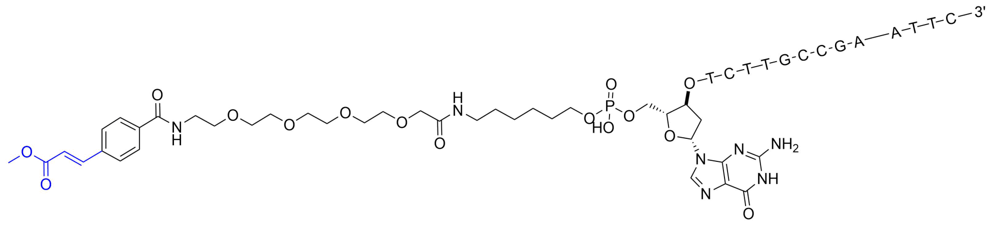

DNA conjugate **2A** was prepared according to the general procedure for optimised on-DNA Heck reaction employing Headpiece **HP-2** and methyl acrylate (1.02 μL).

HRMS (ESI): *m/z* calculated mass: 4827.9775 (acid: 4813.9618); observed mass: 4827.9600 (30%), 4813.9481 (70%)

DNA Conjugate 2B

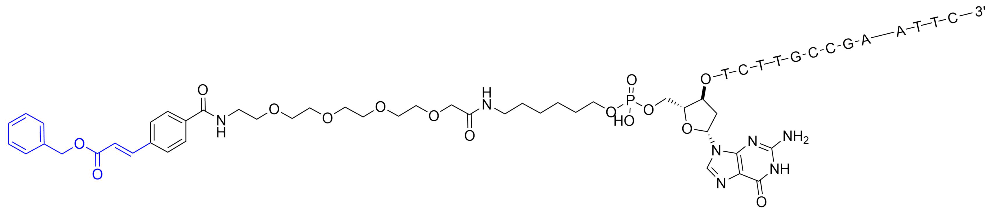

DNA conjugate **2B** was prepared according to the general procedure for optimised on-DNA Heck reaction employing Headpiece **HP-2** and benzyl acrylate (1.65 μL).

HRMS (ESI): *m/z* calculated mass: 4904.0088 (acid 4813.9618); observed mass: 4903.9928 (1%), 4813.9436 (80%)

DNA Conjugate 2C

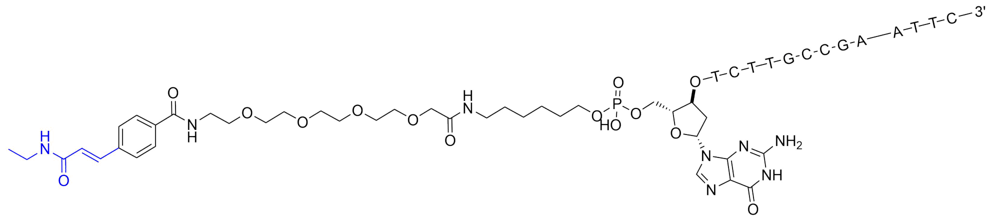

DNA conjugate **2C** was prepared according to the general procedure for optimised on-DNA Heck reaction employing Headpiece **HP-2** and N-ethylacrylamide (1.20 μL).

HRMS (ESI): m/z 4841.0091; observed mass: 4840.9905

DNA Conjugate 2D

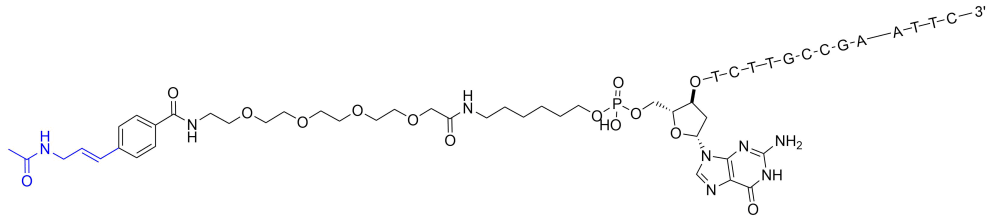

DNA conjugate **2D** was prepared according to the general procedure for optimised on-DNA Heck reaction employing Headpiece **HP-2** and N-allylacetamide (0.78 μL).

HRMS (ESI): m/z calculated mass: 4841.0091; observed mass: 4840.9974

DNA Conjugate 2E

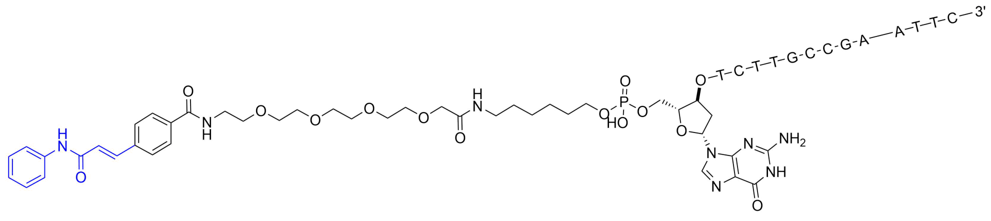

DNA conjugate **2E** was prepared according to the general procedure for optimised on-DNA Heck reaction employing Headpiece **HP-2** and N-phenylacrylamide (1.65 mg). In this instance due to the solid-phase nature of the alkene this was added to the reaction vial first and thus was present during the catalyst pre-activation step; no detrimental influence on reaction progression and/or DNA integrity was observed.

HRMS (ESI): m/z calculated mass: 4889.0091; observed mass: 4888.9932

DNA Conjugate 2F

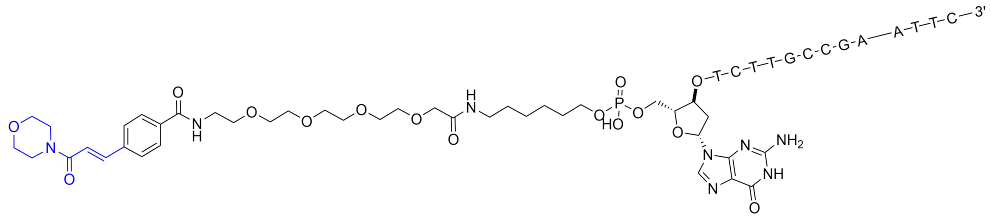

DNA conjugate **2F** was prepared according to the general procedure for optimised on-DNA Heck reaction employing Headpiece **HP-2** and N-acryloylmorpholine (1/41 μL).

HRMS (ESI): m/z calculated mass: 4883.0197; observed mass: 4882.9992

DNA Conjugate 2G/3N

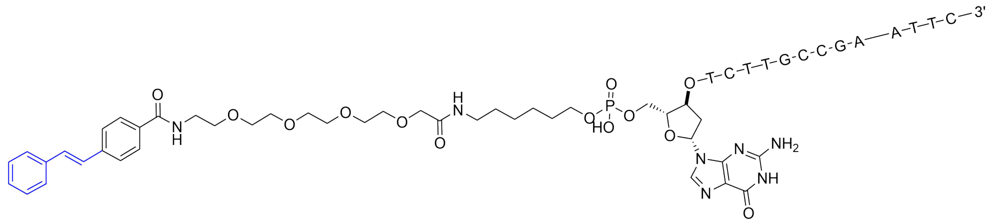

DNA conjugate **2G** was prepared according to the general procedure for optimised on-DNA Heck reaction employing Headpiece **HP-2** and styrene (1.29 μL).

HRMS (ESI): m/z calculated mass: 4846.0033; observed mass: 4845.9896

OR

DNA conjugate **3N** was prepared according to the general procedure for optimised on-DNA Heck reaction employing Headpiece **HP-3** and bromobenzene (1.15 μL)

HRMS (ESI): m/z calculated mass: 4846.0033; observed mass: 4845.9443 (80%)

DNA Conjugate 2H

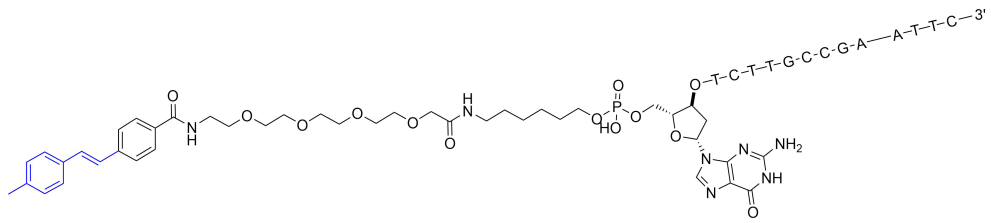

DNA conjugate **2H** was prepared according to the general procedure for optimised on-DNA Heck reaction employing Headpiece **HP-2** and 4-methylstyrene (1.42 μL).

HRMS (ESI): m/z calculated mass: 4860.0189; observed mass: 4859.9713

DNA Conjugate 2I/3O

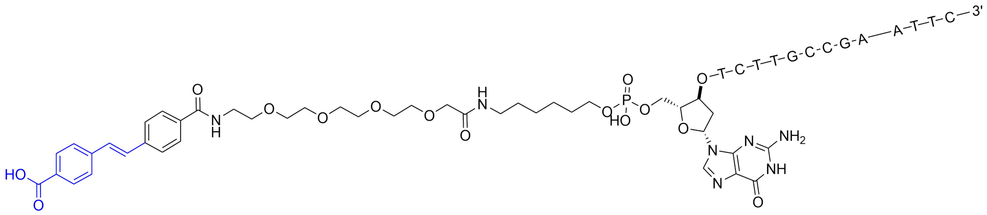

DNA conjugate **2I** was prepared according to the general procedure for optimised on-DNA Heck reaction employing Headpiece **HP-2** and 4-vinylbenzoic acid (1.63 mg). In this instance due to the solid-phase nature of the alkene this was added to the reaction vial first and thus was present during the catalyst pre-activation step; no detrimental influence on reaction progression and/or DNA integrity was observed.

HRMS (ESI): m/z calculated mass: 4889.9931; observed mass: 4889.9475

OR

DNA conjugate **3O** was prepared according to the general procedure for optimised on-DNA Heck reaction employing Headpiece **HP-3** and 4-iodobenzoic acid (2.73 mg). In this instance due to the solid-phase nature of the aryl halide this was added to the reaction vial first and thus was present during the catalyst pre-activation step; no detrimental influence on reaction progression and/or DNA integrity was observed.

HRMS (ESI): m/z calculated mass: 4889.9931 (di-substituted: 5010.0143); observed mass: 4889.9777 (85%), 5009.9993 (15%)

DNA Conjugate 2J

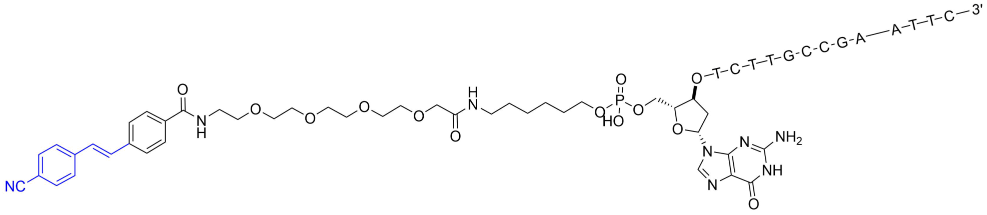

DNA conjugate **2J** was prepared according to the general procedure for optimised on-DNA Heck reaction employing Headpiece **HP-2** and 4-cyanostyrene (1.30 μL).

HRMS (ESI): m/z calculated mass: 4870.9985; observed mass: 4870.9538

DNA Conjugate 2K

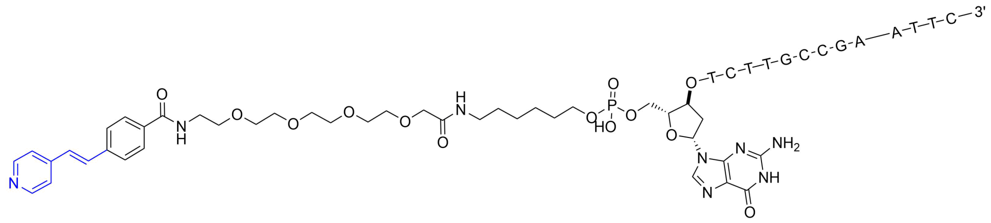

DNA conjugate **2K** was prepared according to the general procedure for optimised on-DNA Heck reaction employing Headpiece **HP-2** and 4-vinylpyridine (1.14 μL).

HRMS (ESI): m/z calculated mass: 4846.9985; observed mass: 4846.9564

DNA Conjugate 2L

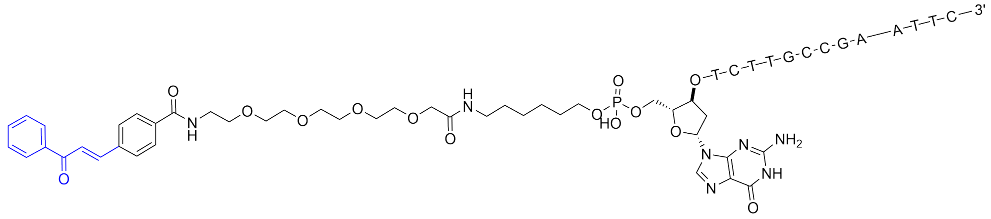

DNA conjugate **2L** was prepared according to the general procedure for optimised on-DNA Heck reaction employing Headpiece **HP-2** and acrylophenone (1.45 mg).

HRMS (ESI): m/z calculated mass: 4873.9982; observed mass: 4873.9832

DNA Conjugate 2M

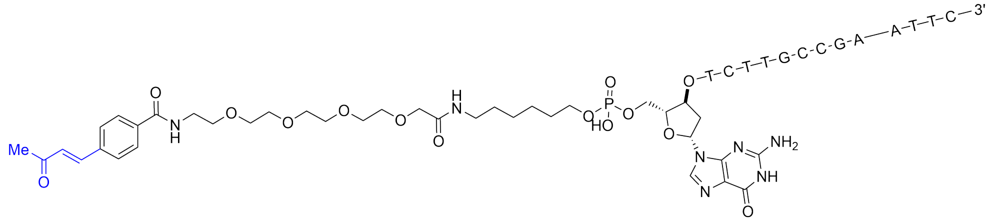

DNA conjugate **2M** was prepared according to the general procedure for optimised on-DNA Heck reaction employing Headpiece **HP-2** and methyl vinyl ketone (0.91 μL).

HRMS (ESI): m/z calculated mass: 4811.9826; observed mass: 4811.9740

DNA Conjugate 3P

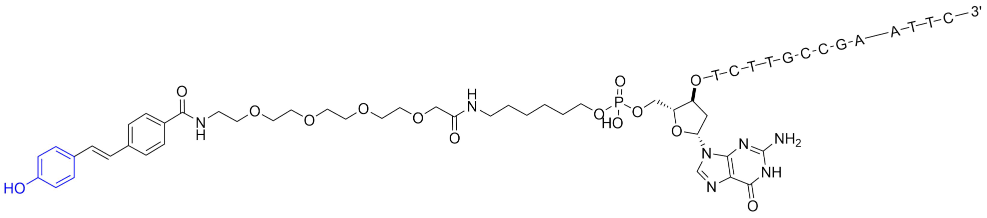

DNA conjugate **3P** was prepared according to the general procedure for optimised on-DNA Heck reaction employing Headpiece **HP-3** and 4-iodophenol (2.42 mg). In this instance due to the solid-phase nature of the alkene this was added to the reaction vial first and thus was present during the catalyst pre-activation step; no detrimental influence on reaction progression and/or DNA integrity was observed.

HRMS (ESI): m/z calculated mass: 4861.9982 (di-substituted: 4954.0244); observed mass: 4861.9861 (9%), 4954.0216 (91%)

DNA Conjugate 3Q

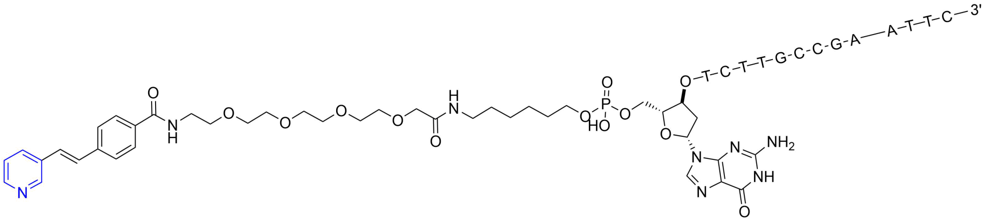

DNA conjugate **3Q** was prepared according to the general procedure for optimised on-DNA Heck reaction employing Headpiece **HP-3** and 3-iodopyridine (2.26 mg). In this instance due to the solid-phase nature of the alkene this was added to the reaction vial first and thus was present during the catalyst pre-activation step; no detrimental influence on reaction progression and/or DNA integrity was observed.

HRMS (ESI): m/z calculated mass: 4846.9985; observed mass: 4846.9868 (61%)

6 Chromatograms and Spectra

6.1 NMR Spectra

6.1.1 2-(2-(2-(2-hydroxyethoxy)ethoxy)ethoxy)ethyl 4-methylbenzenesulfonate

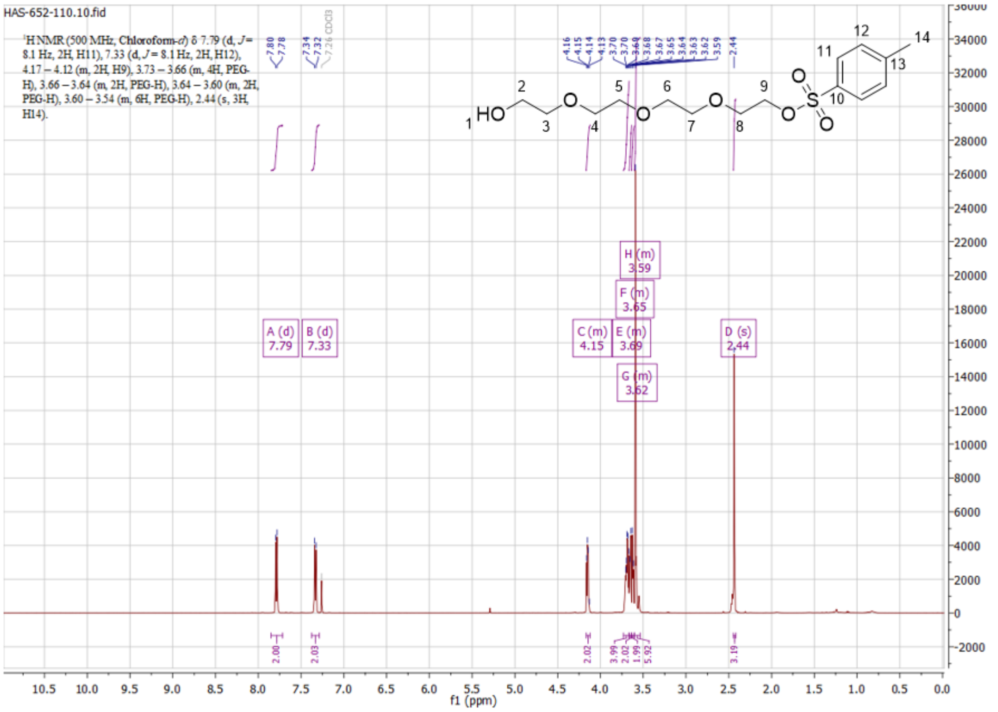

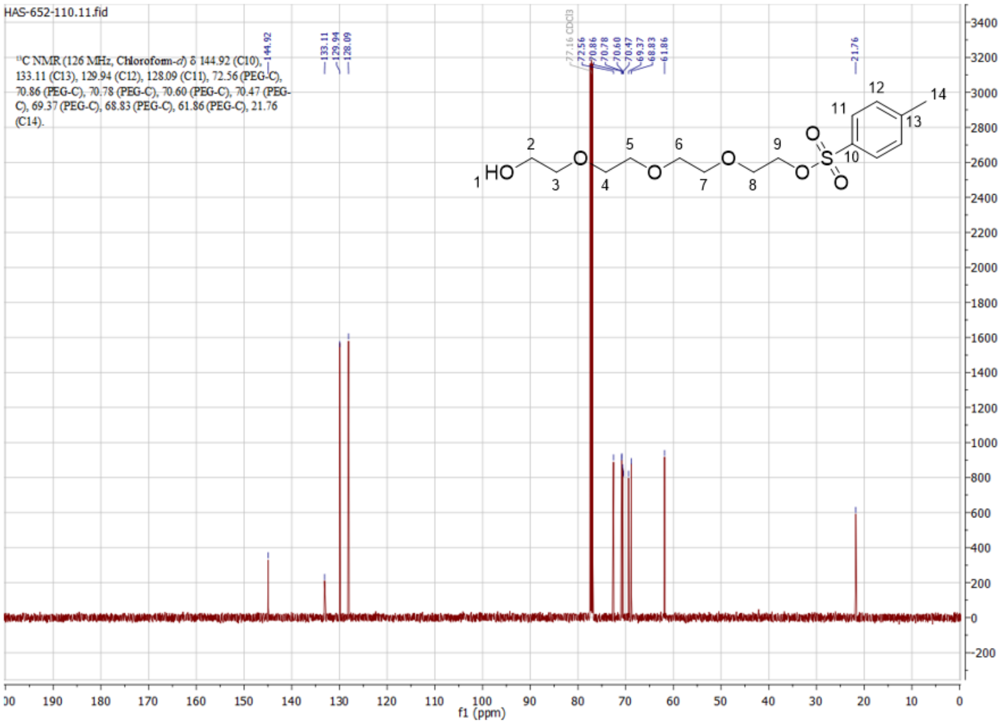

6.1.2 2-(2-(2-(2-(4-iodophenoxy)ethoxy)ethoxy)ethoxy)ethan-1-ol

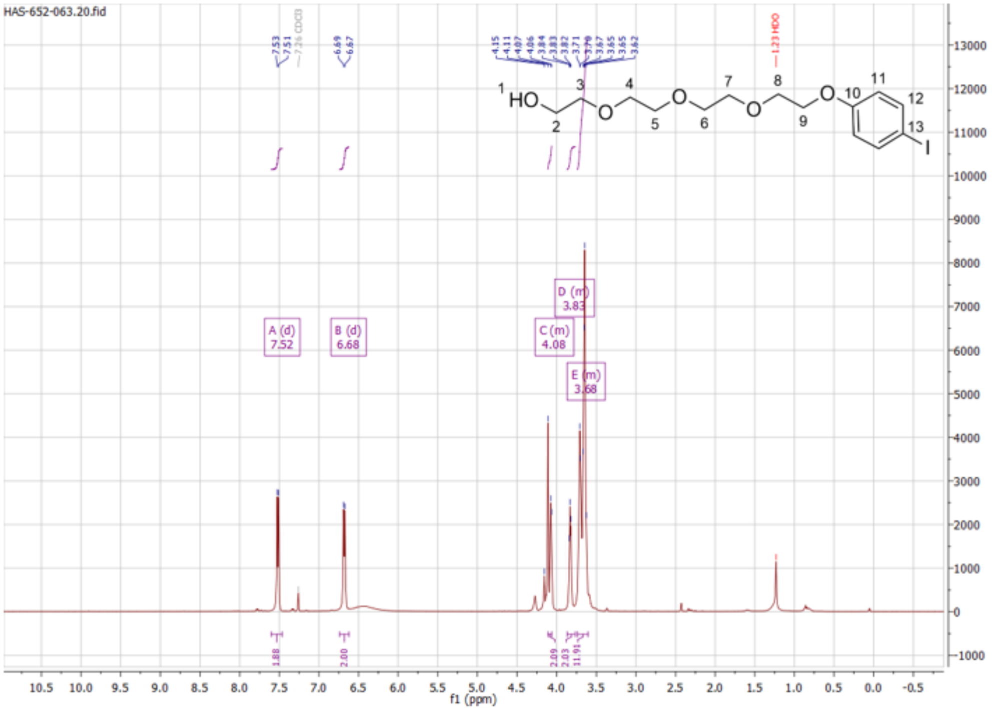

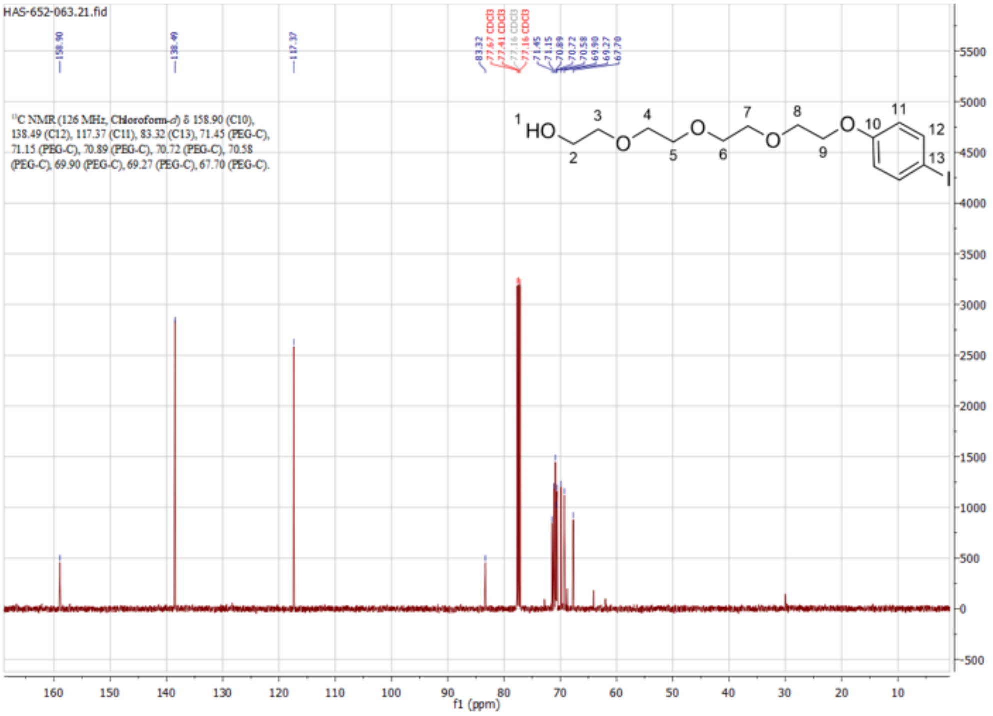

6.1.3 Ethyl 14-(4-iodophenoxy)-3,6,9,12-tetraoxatetradecanoate

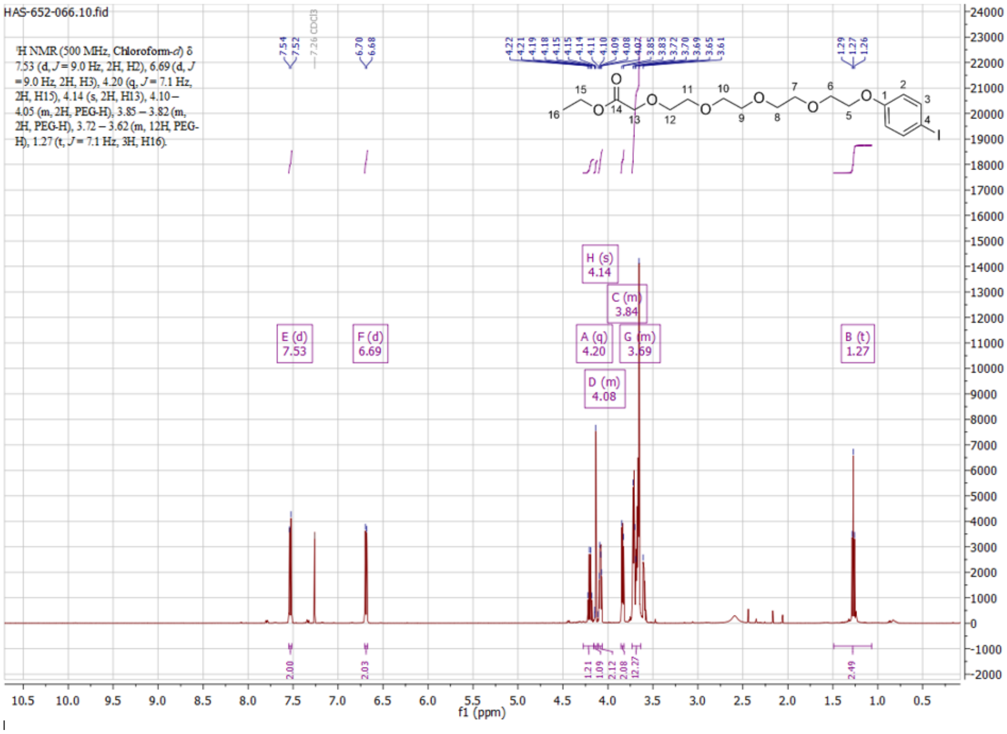

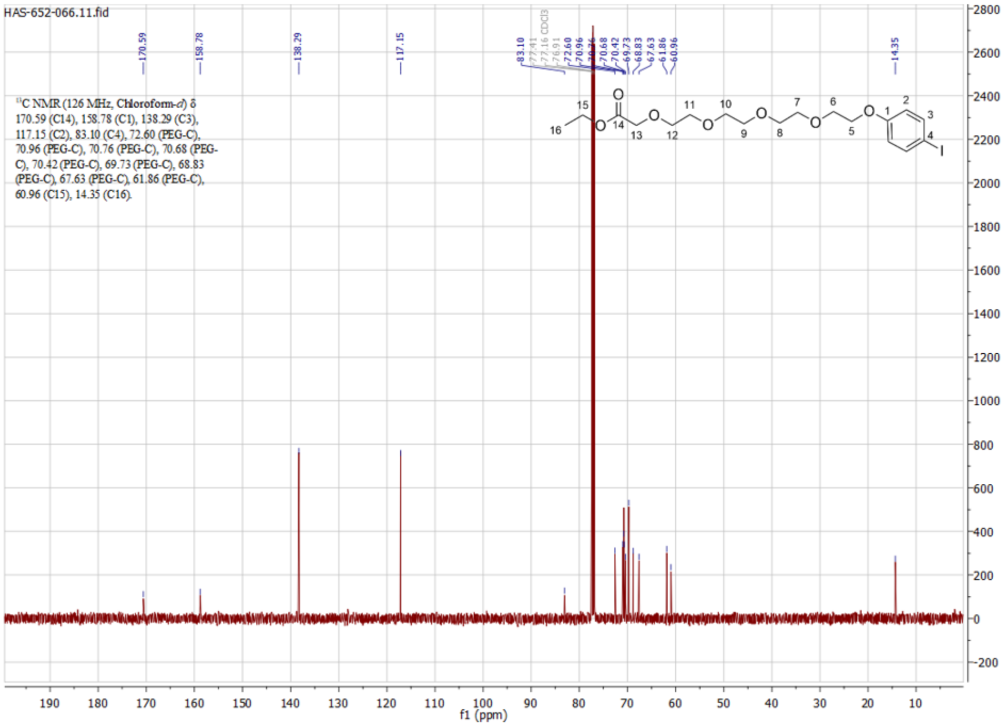

6.1.4 14-(4-iodophenoxy)-3,6,9,12-tetraoxatetradecanoic acid

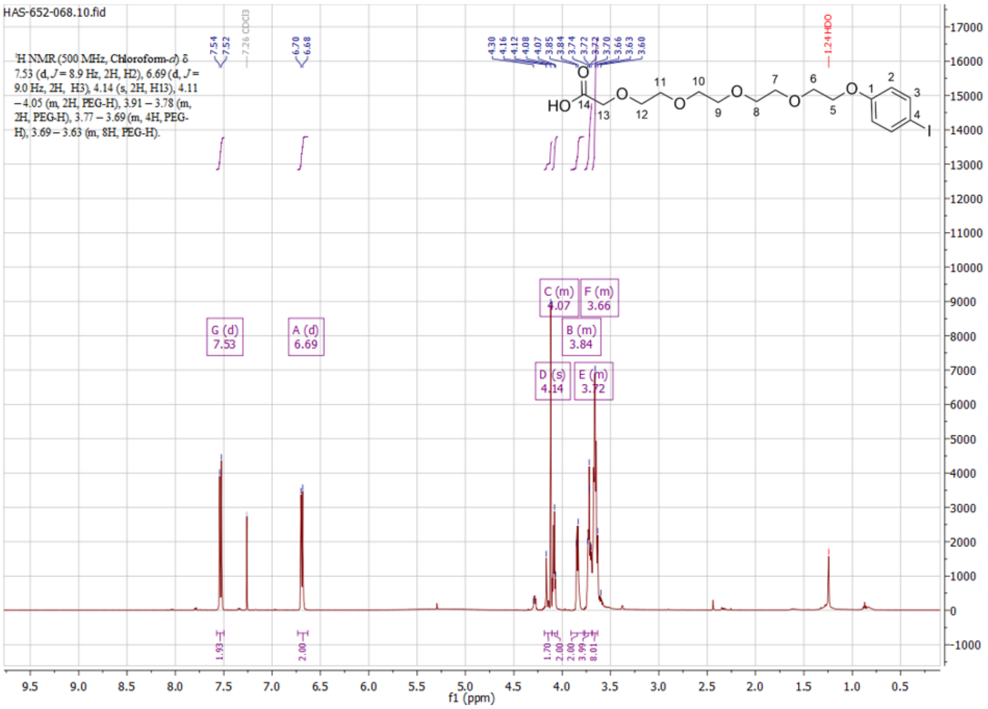

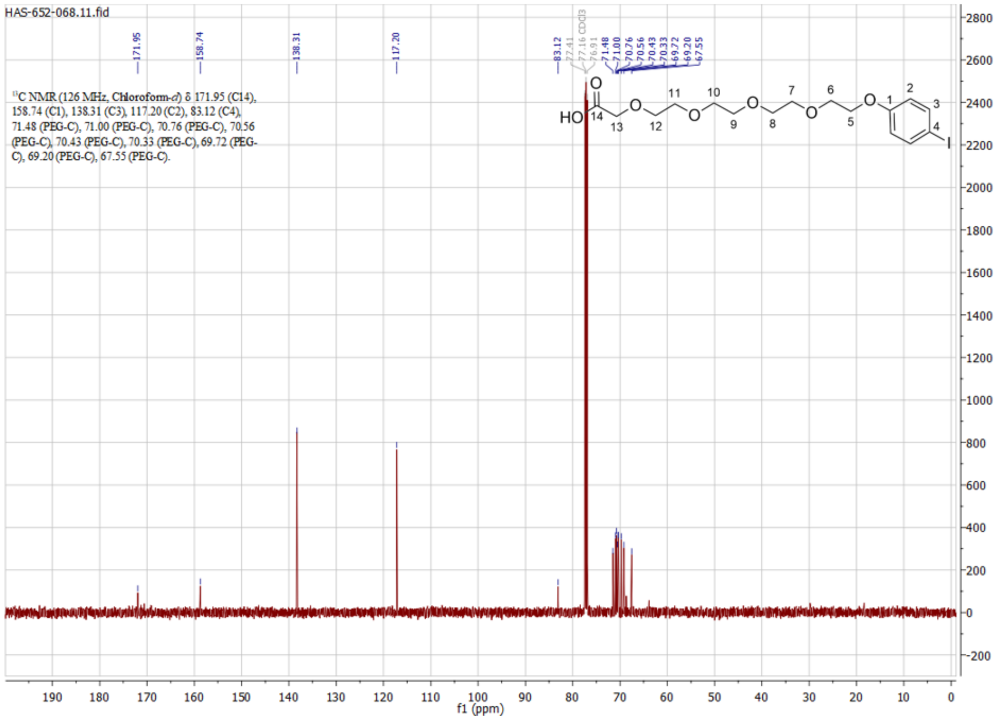

6.2 Oligonucleotide Chomatograms and Mass Spectra

DNA Conjugate HP1

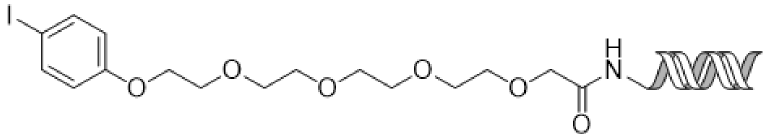

Calculated mass: 4842.8421
Observed mass: 4842.8233

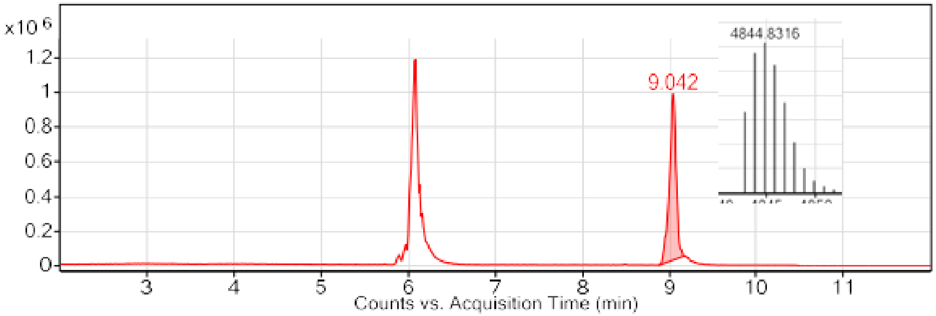

DNA Conjugate PEG4-NH_2

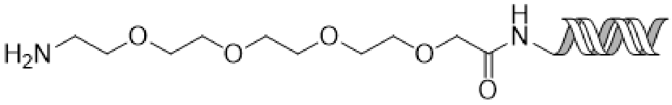

Calculated mass: 4639.9301
Observed mass: 4639.9183

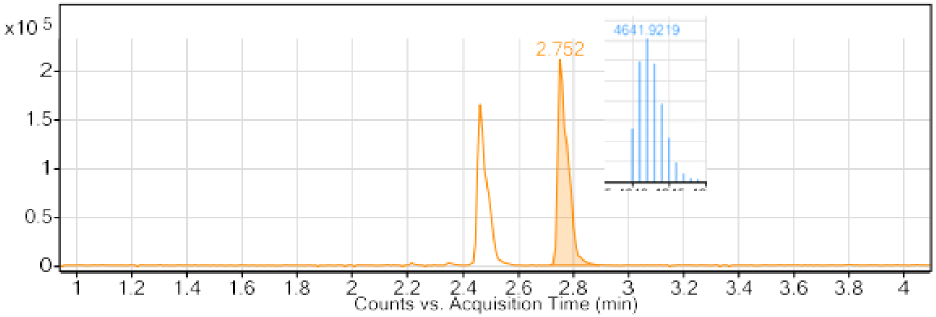

DNA Conjugate HP-2

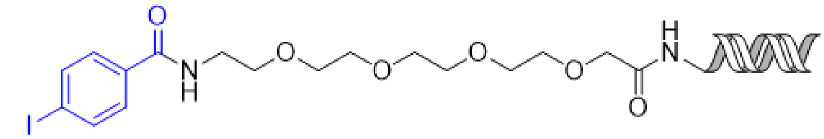

Calculated mass: 4869.8530
Observed mass: 4869.8476

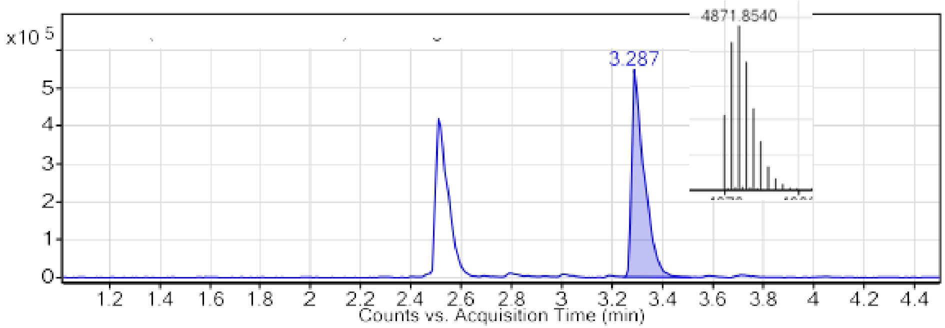

DNA Conjugate HP-3

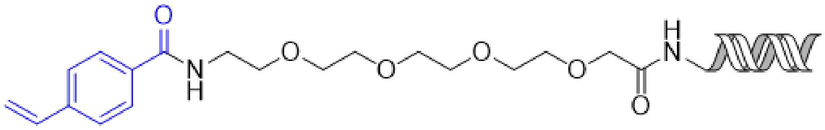

Calculated mass: 4769.9720
Observed mass: 4769.9276

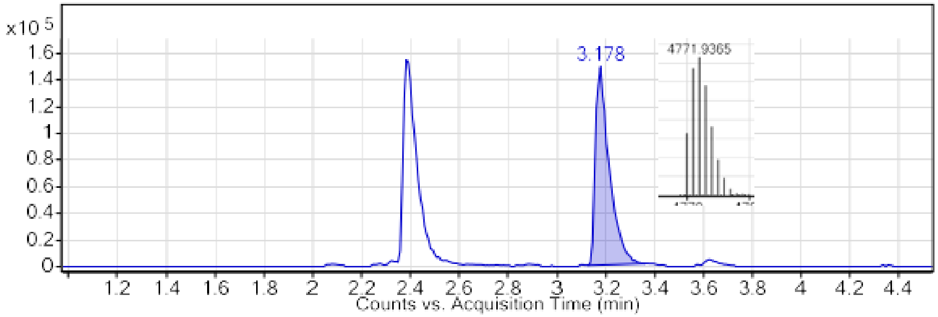

DNA Conjugate 1A

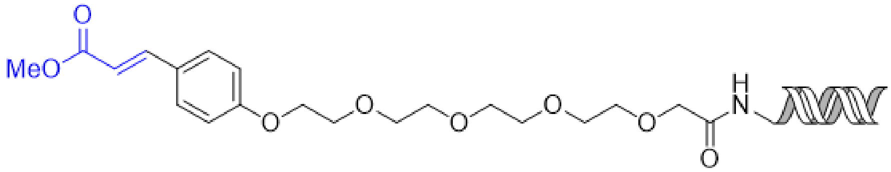

Calculated mass: 4800.9666 (acid: 4786.9509)
Observed mass: 4800.9272 (51%), 4786.9158 (49%)

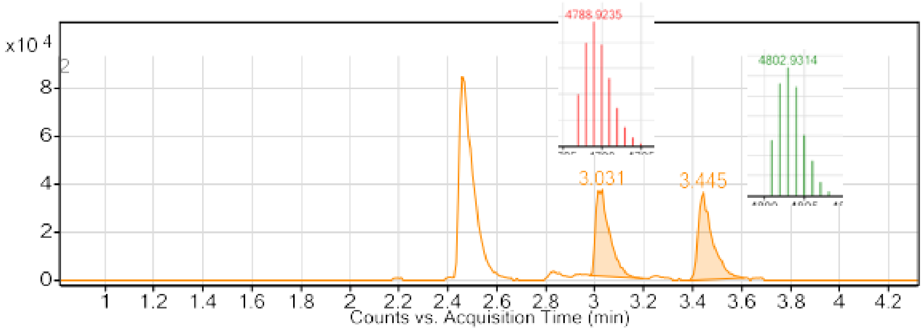

DNA Conjugate 1B

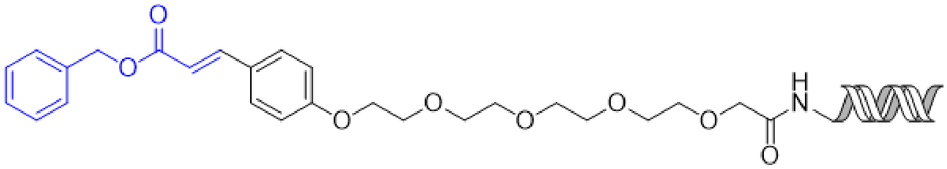

Calculated mass: 4876.9979 (acid: 4786.9509)
Observed mass: 4876.9298 (49%), 4786.8529 (43%)

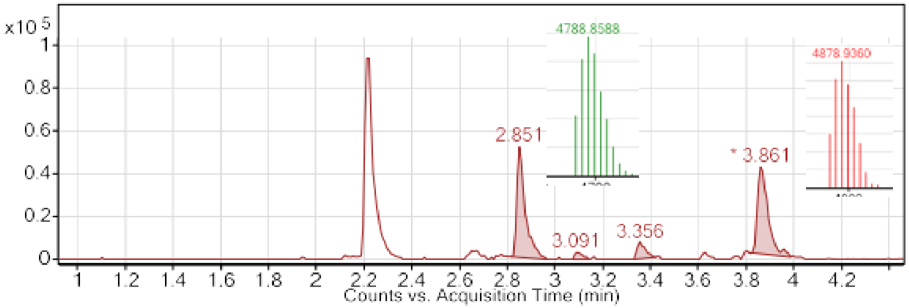

DNA Conjugate 1C

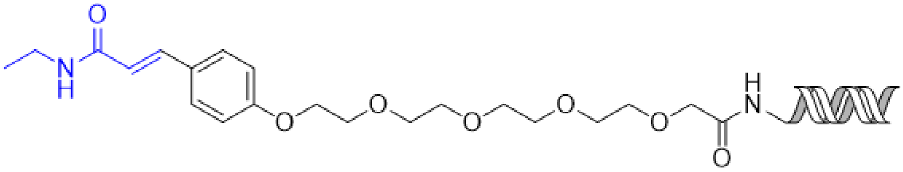

Calculated mass: 4813.9982
Observed mass: 4813.9550

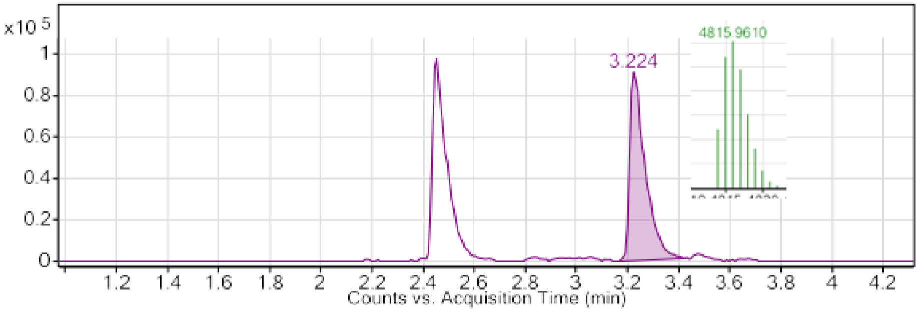

DNA Conjugate 1D

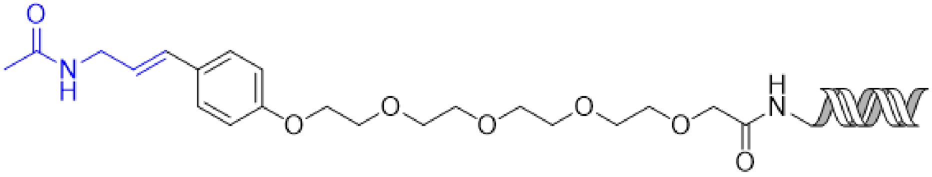

Calculated mass: 4813.9982
Observed mass: 4813.9607

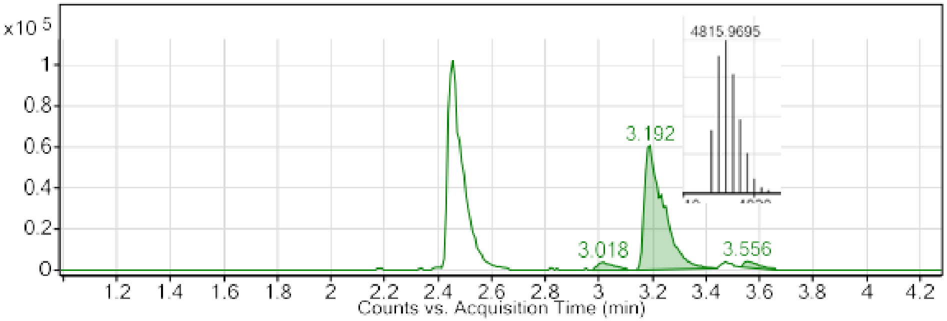

DNA Conjugate 1E

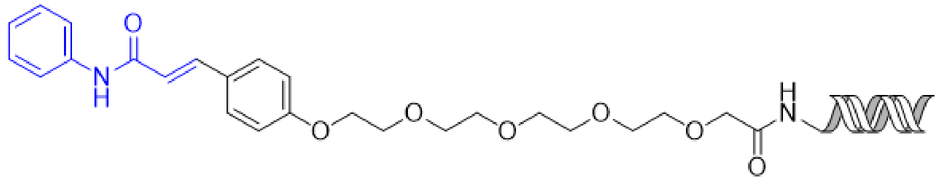

Calculated mass: 4861.9982

Observed mass: 4861.9578

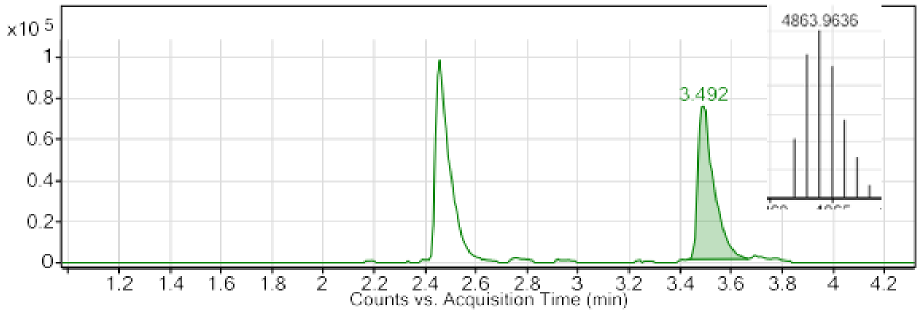

DNA Conjugate 1F

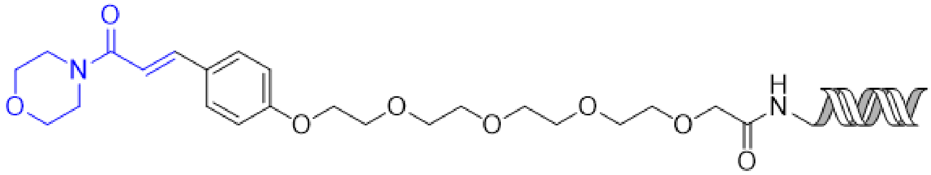

Calculated mass: 4856.0088

Observed mass: 4855.9637

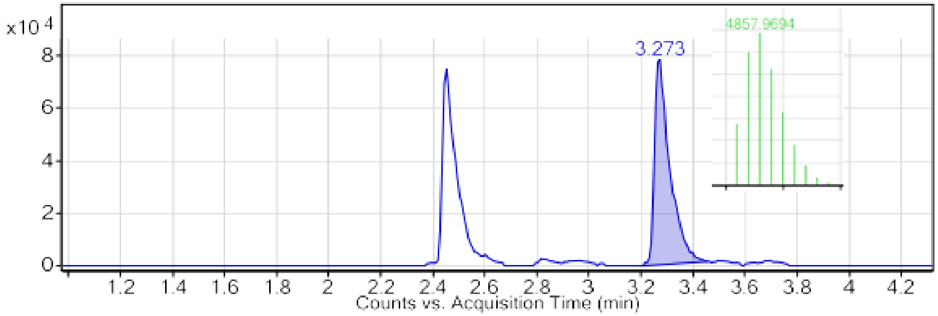

DNA Conjugate 1G

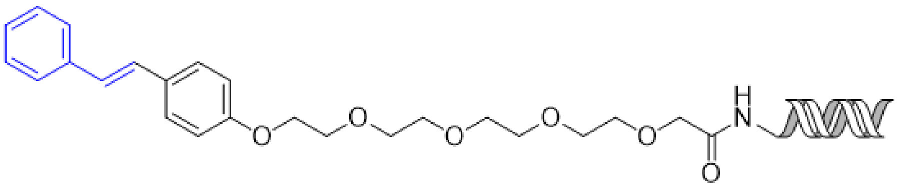

Calculated mass: 4818.9924
Observed mass: 4818.9153

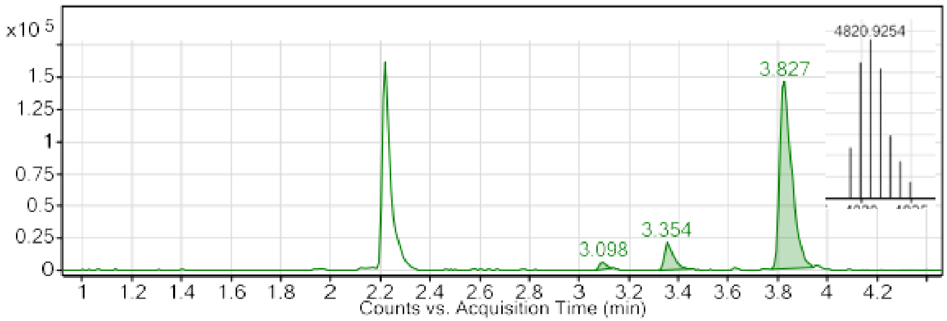

DNA Conjugate 2A

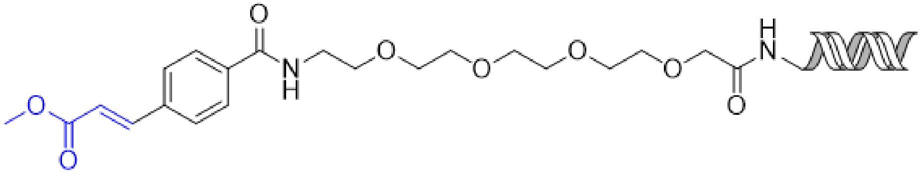

Calculated mass: 4827.9775 (acid: 4813.9618)
Observed mass: 4827.9600 (30%), 4813.9481 (70%)

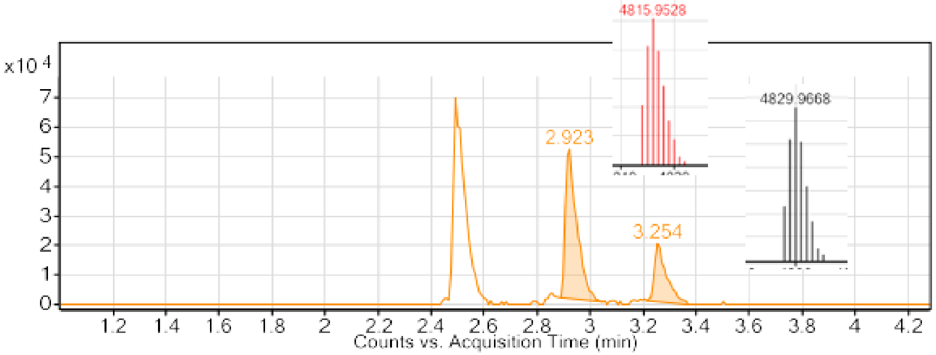

DNA Conjugate 2B

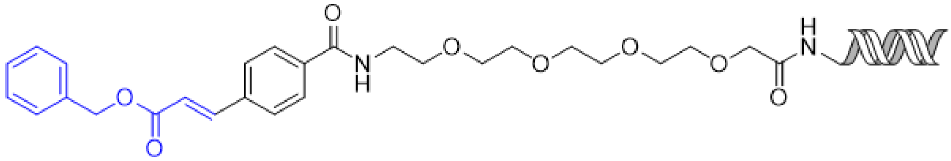

Calculated mass: 4904.0088, (acid: 4813.9618)
Observed mass: 4903.9928 (1%), 4813.9436 (80%)

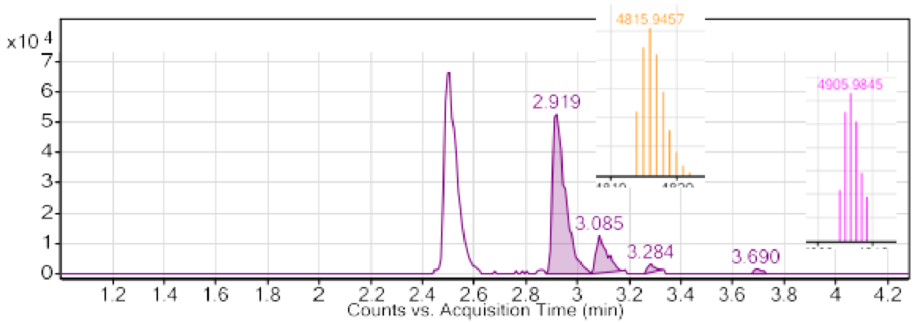

DNA Conjugate 2C

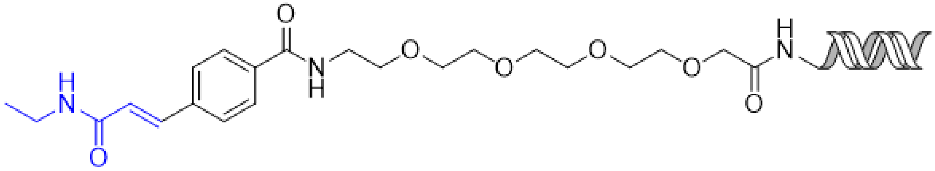

Calculated mass: 4841.0091
Observed mass: 4840.9905

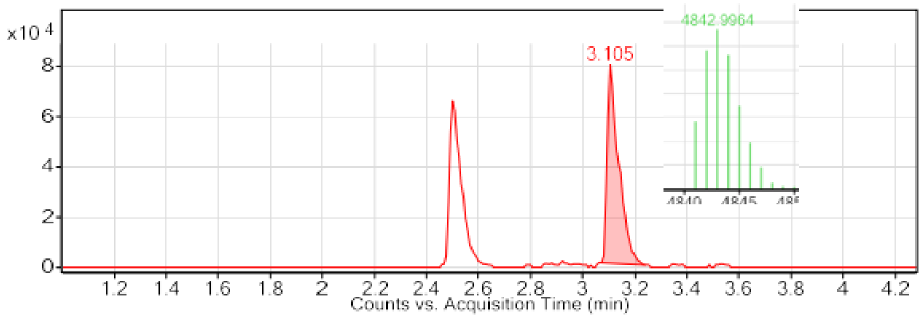

DNA Conjugate 2D

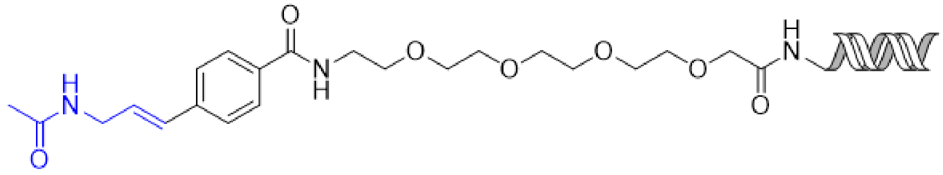

Calculated mass: 4841.0091
Observed mass: 4840.9974

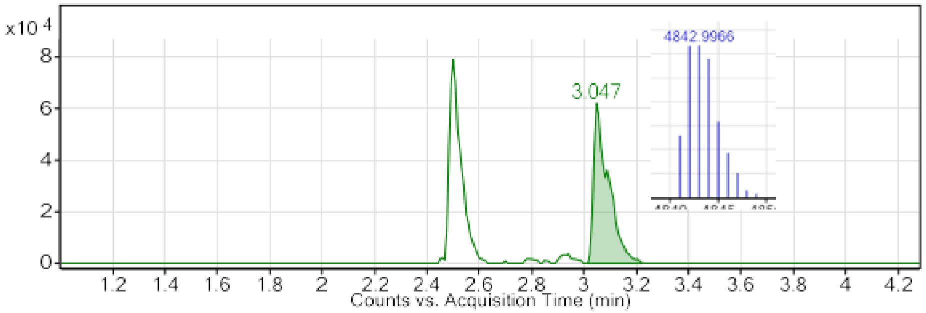

DNA Conjugate 2E

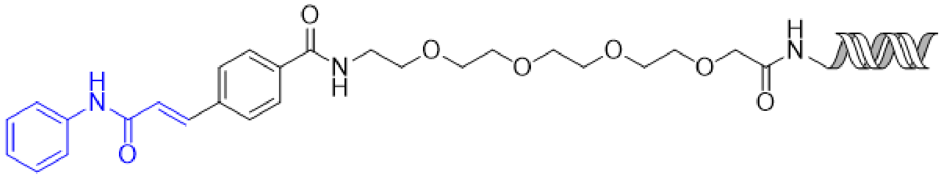

Calculated mass: 4889.0091
Observed mass: 4888.9932

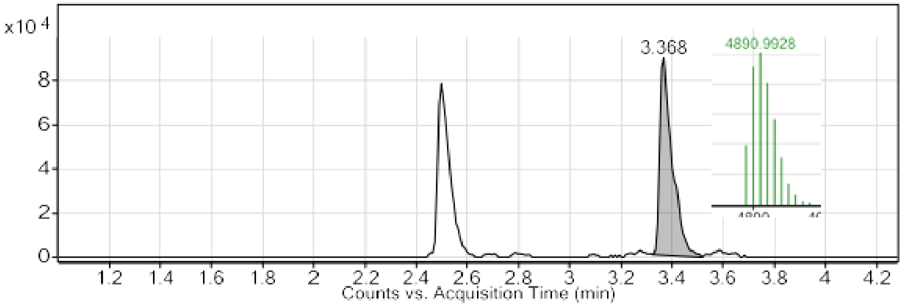

DNA Conjugate 2F

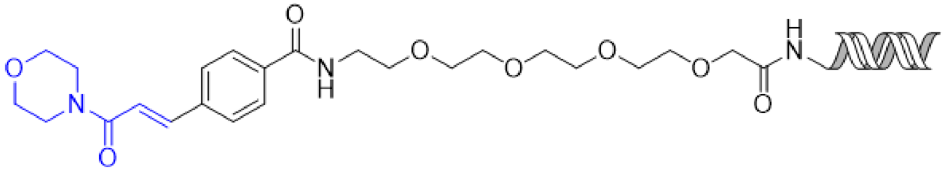

Calculated mass: 4883.0197
Observed mass: 4882.9992

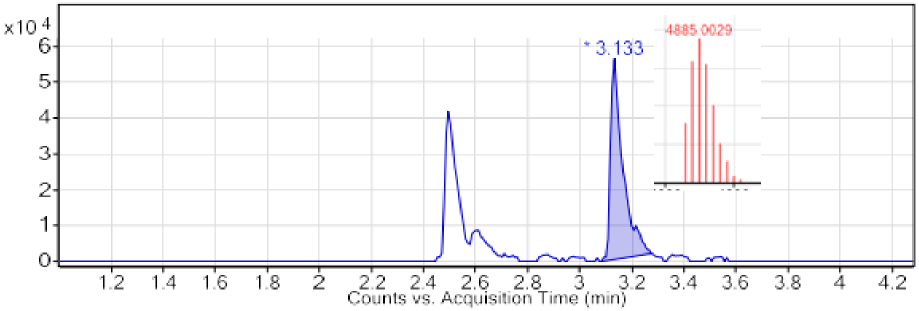

DNA Conjugate 2G from HP-2

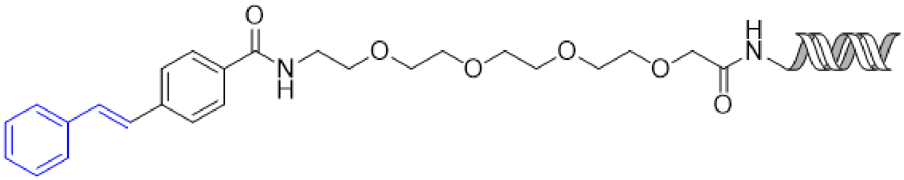

Calculated mass: 4846.0033
Observed mass: 4845.9896

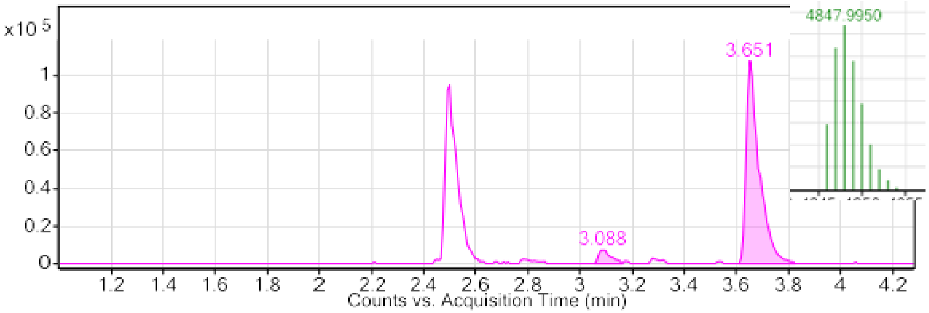

DNA Conjugate 3N from HP-3

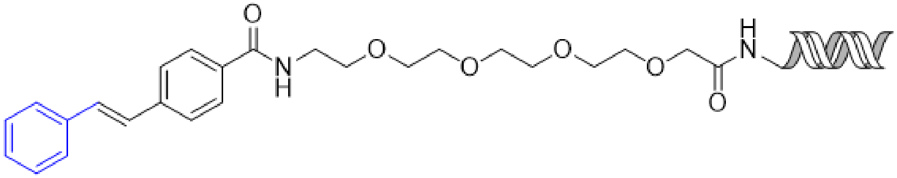

Calculated mass: 4846.0033
Observed mass: 4845.9443

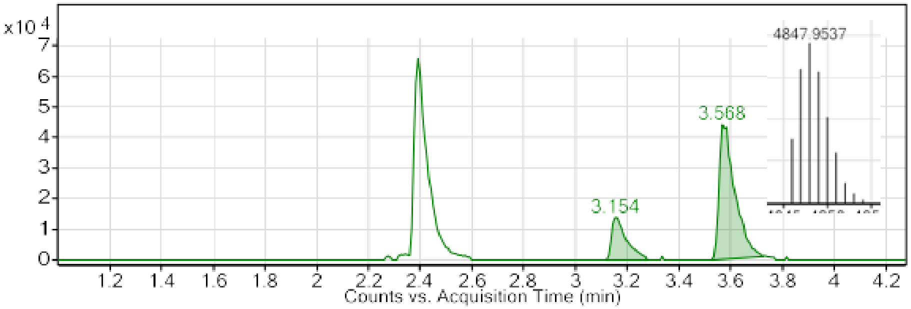

DNA Conjugate 2H

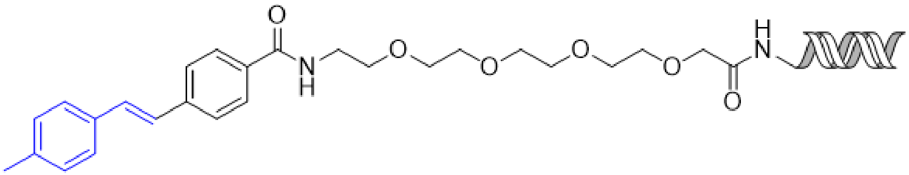

Calculated mass: 4860.0189
Observed mass: 4859.9713

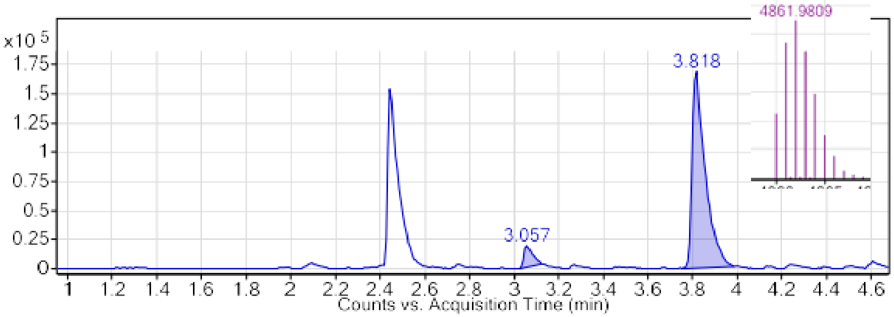

DNA Conjugate 2I from HP-2

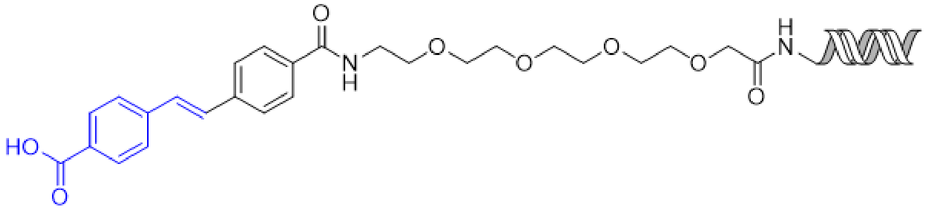

Calculated mass: 4889.9931

Observed mass: 4889.9475

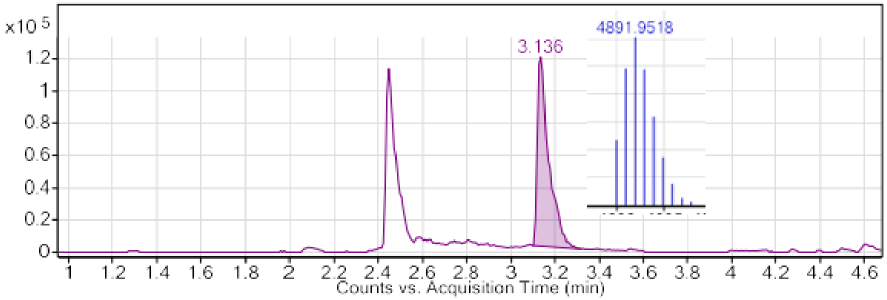

DNA Conjugate 3O from HP-3

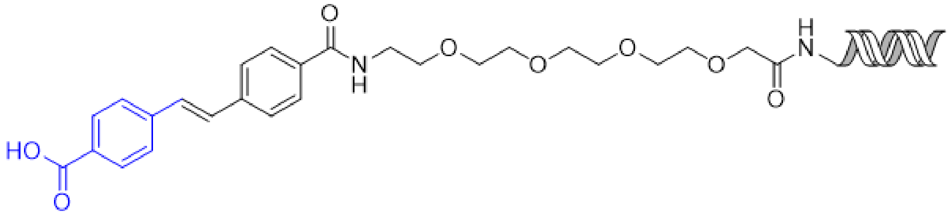

Calculated mass: 4889.9931, (double: 5010.0143)

Observed mass: 4889.9777 (85%), 5009.9993 (15%)

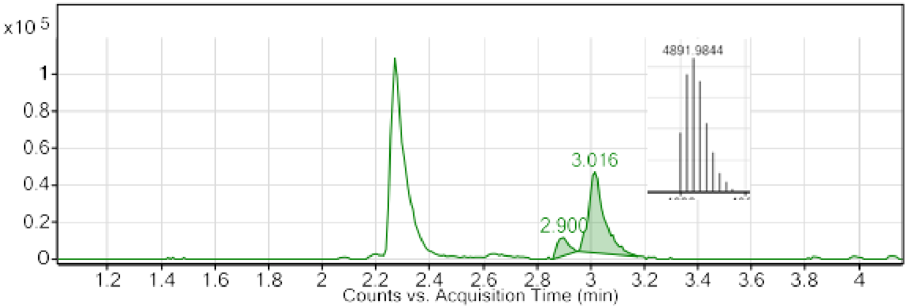

DNA Conjugate 2J

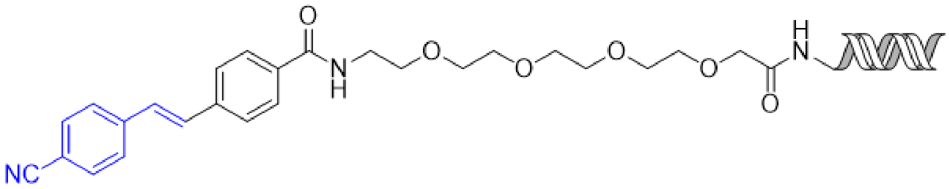

Calculated mass: 4870.9985
Observed mass: 4870.9538

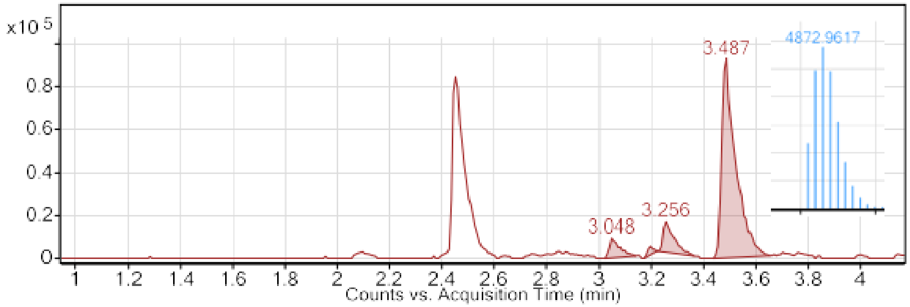

DNA Conjugate 2K

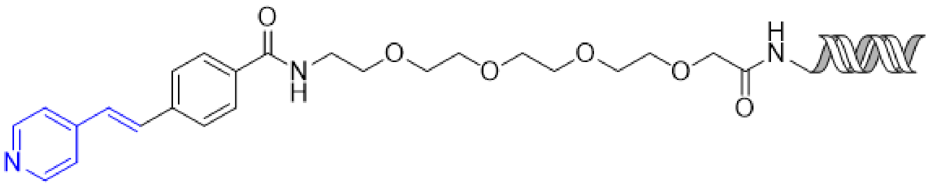

Calculated mass: 4846.9985
Observed mass: 4846.9564

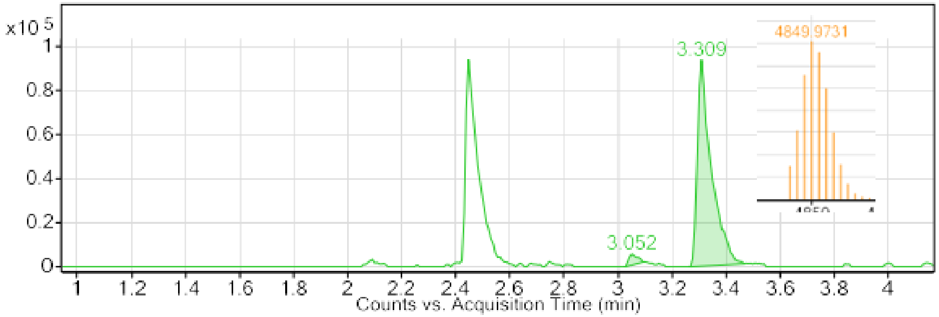

DNA Conjugate 2L

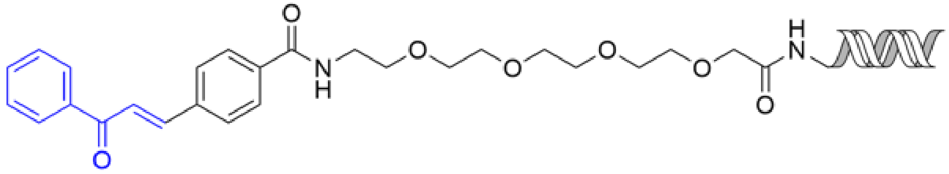

Calculated mass: 4873.9982
Observed mass: 4873.9832

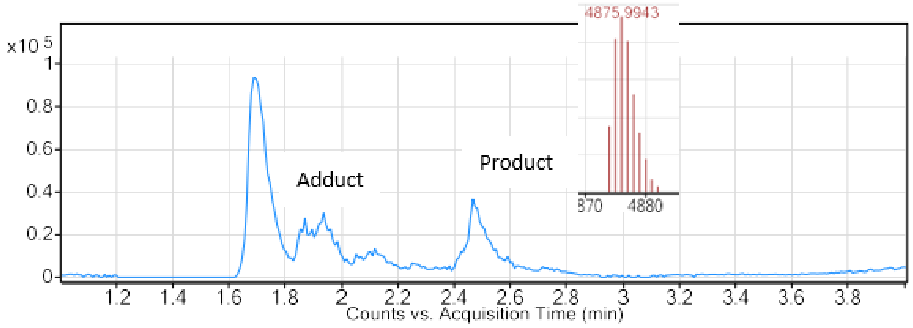

DNA Conjugate 2M

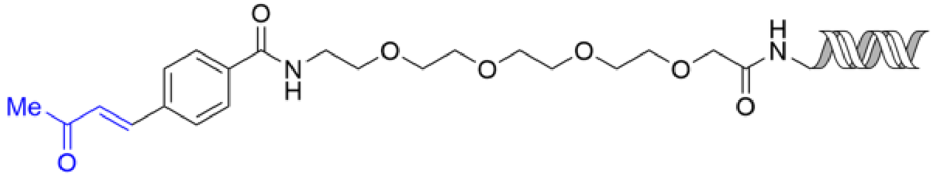

Calculated mass: 4811.9826
Observed mass: 4811.9740

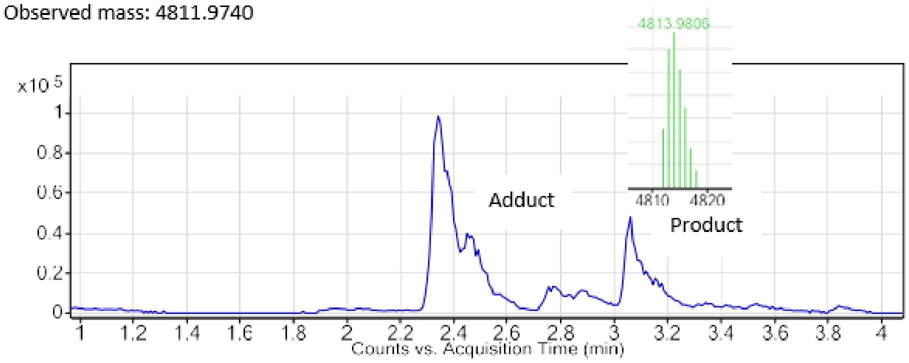

DNA Conjugate 3P

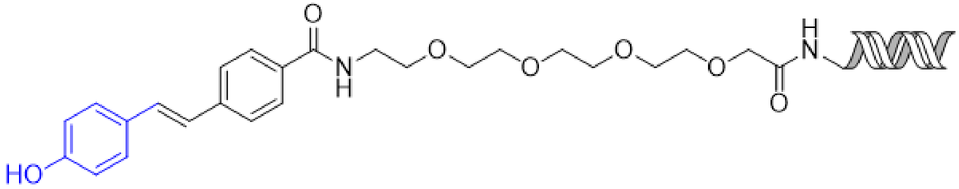

Calculated mass: 4861.9982, (double: 4954.0244)

Observed mass: 4861.9861 (9%), 4954.0216 (91%)

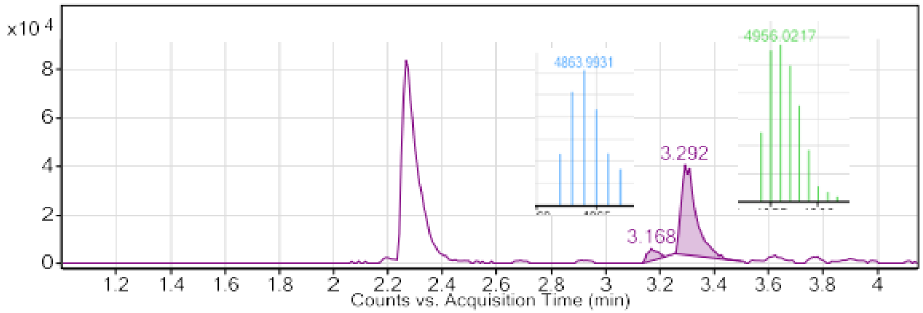

DNA Conjugate 3Q

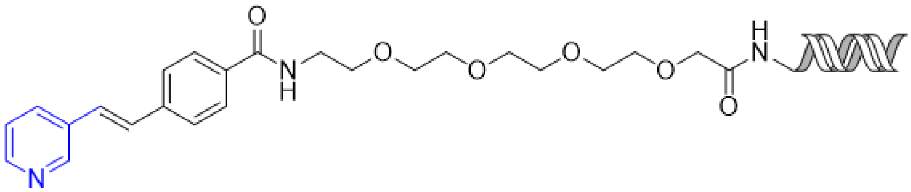

Calculated mass: 4846.9985

Observed mass: 4846.9868 (61%)

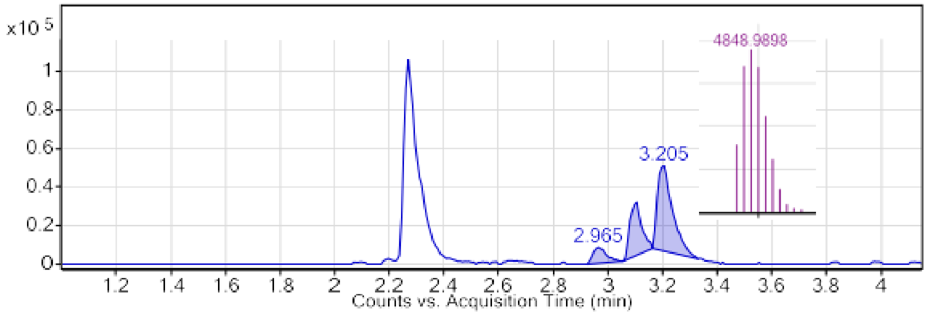

Supplement: Supplementary file 1 — bc3c00051_si_001.pdf [file bc3c00051_si_001.pdf]
