# Supplementary figures and images for: Development of a Micellar-Promoted Heck Reaction for the Synthesis of DNA-Encoded Libraries (part 2 of 2)
Source: Bioconjug Chem. 2023 Mar 8;34(4):756–63. doi: 10.1021/acs.bioconjchem.3c00051 (PMC10119937; doi:10.1021/acs.bioconjchem.3c00051)

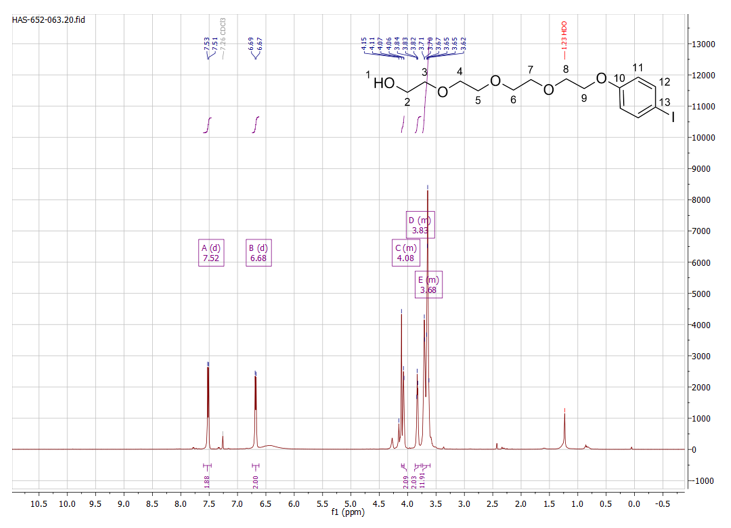

Supplement: Supplementary file 2 — bc3c00051_si_002.zip [file bc3c00051_si_002.zip › Chrom_Spectra/PEG4-PhI_1Hf.PNG]

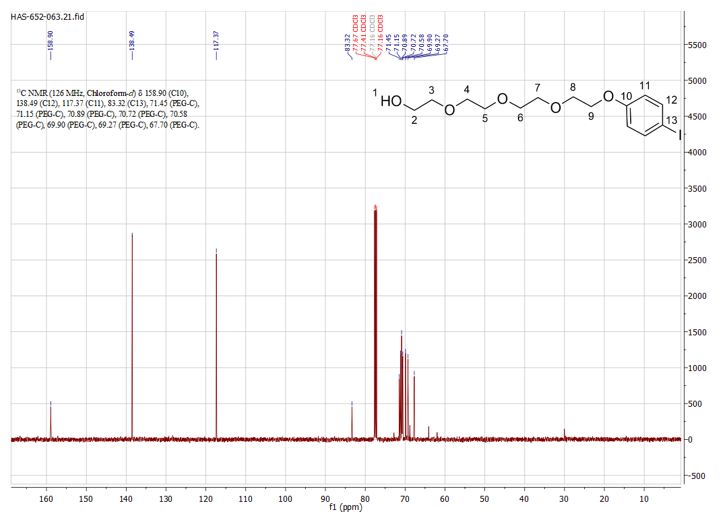

Supplement: Supplementary file 2 — bc3c00051_si_002.zip [file bc3c00051_si_002.zip › Chrom_Spectra/PEG4-PhI_13Cf.PNG]

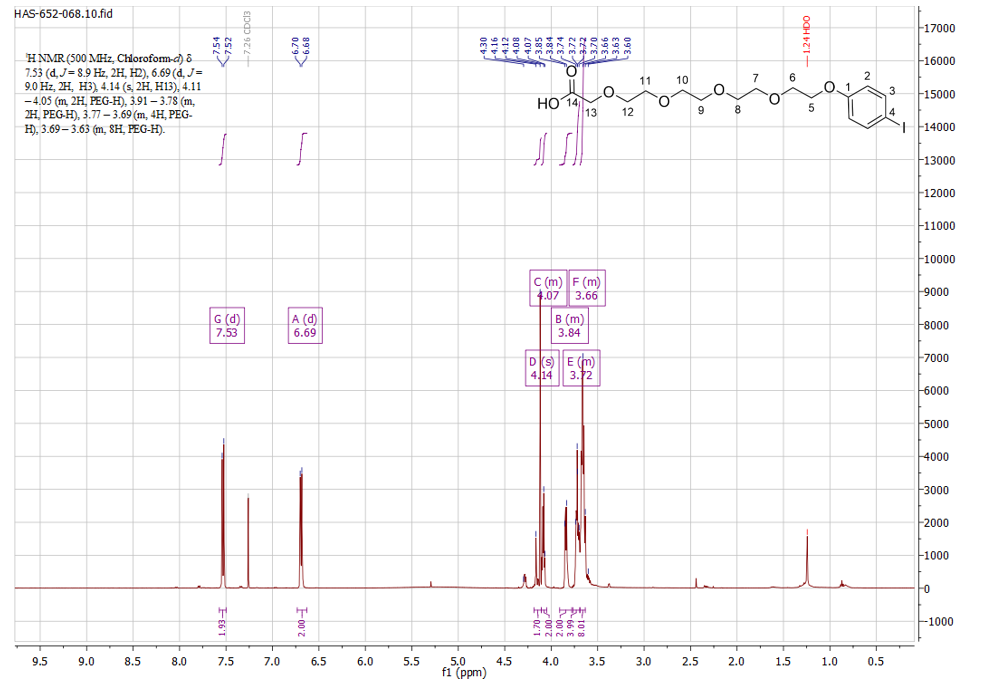

Supplement: Supplementary file 2 — bc3c00051_si_002.zip [file bc3c00051_si_002.zip › Chrom_Spectra/Acid-Peg4-PhI_1Hf.PNG]

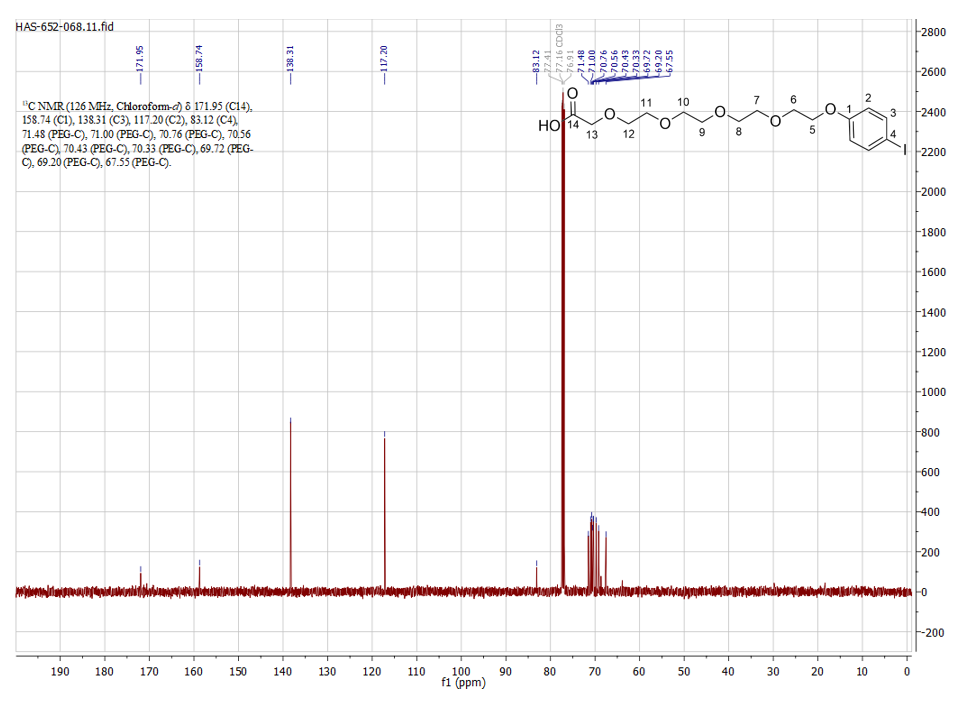

Supplement: Supplementary file 2 — bc3c00051_si_002.zip [file bc3c00051_si_002.zip › Chrom_Spectra/Acid-Peg4-PhI_13Cf.PNG]

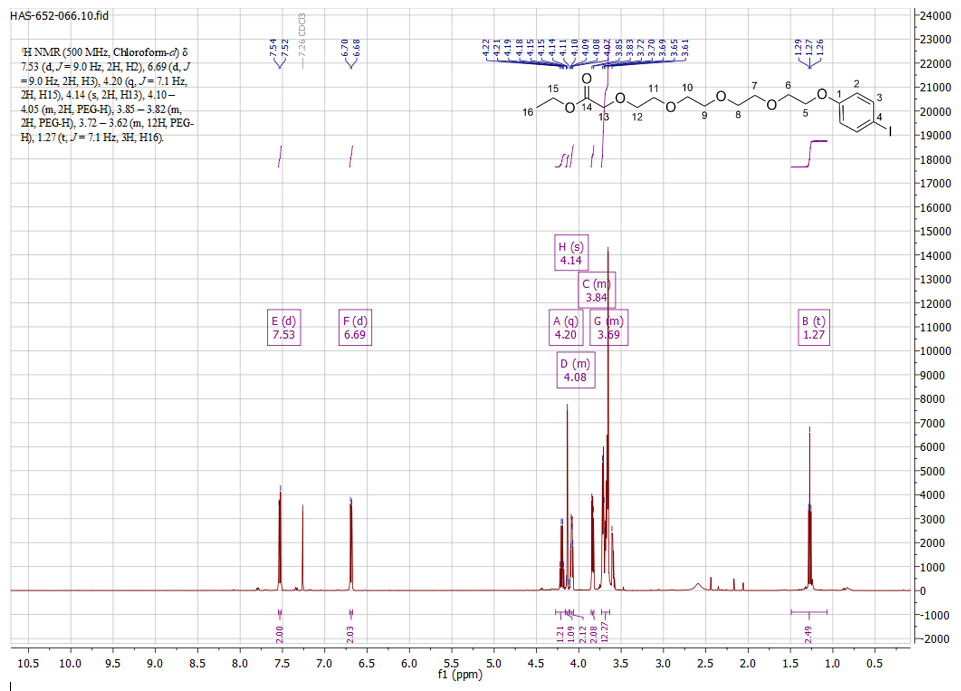

Supplement: Supplementary file 2 — bc3c00051_si_002.zip [file bc3c00051_si_002.zip › Chrom_Spectra/Ester-Peg4-PhI_1Hf.PNG]

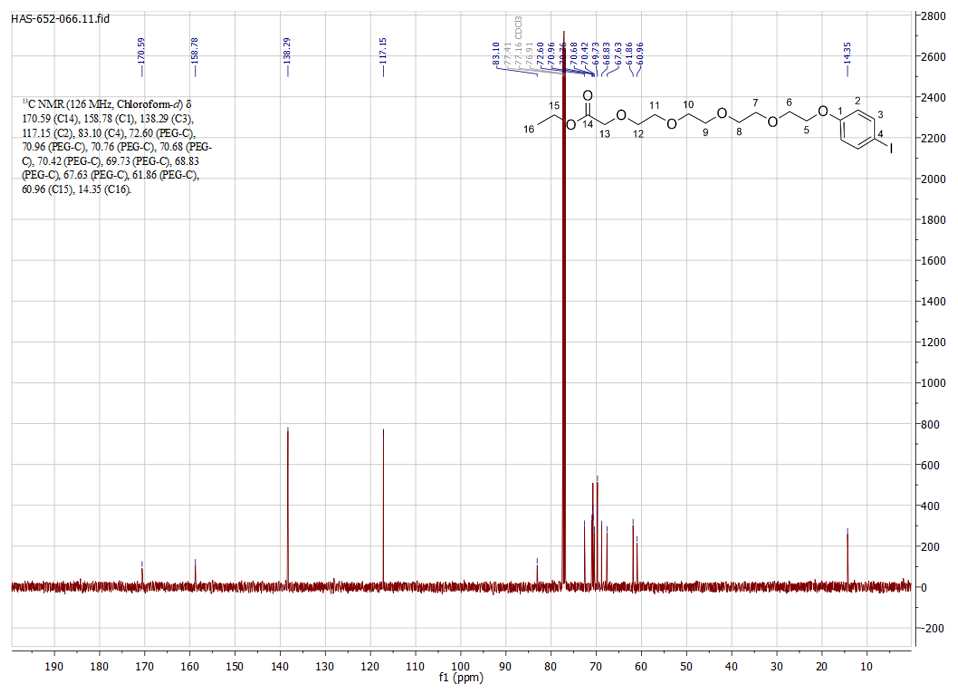

Supplement: Supplementary file 2 — bc3c00051_si_002.zip [file bc3c00051_si_002.zip › Chrom_Spectra/Ester-Peg4-PhI_13Cf.PNG]

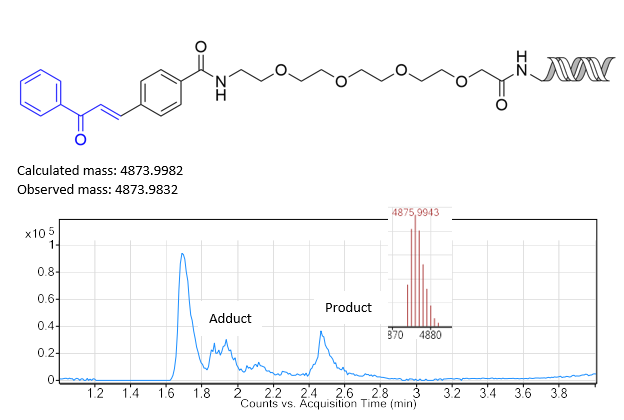

Supplement: Supplementary file 2 — bc3c00051_si_002.zip [file bc3c00051_si_002.zip › Chrom_Spectra/AcrylophenoneChrom.PNG]

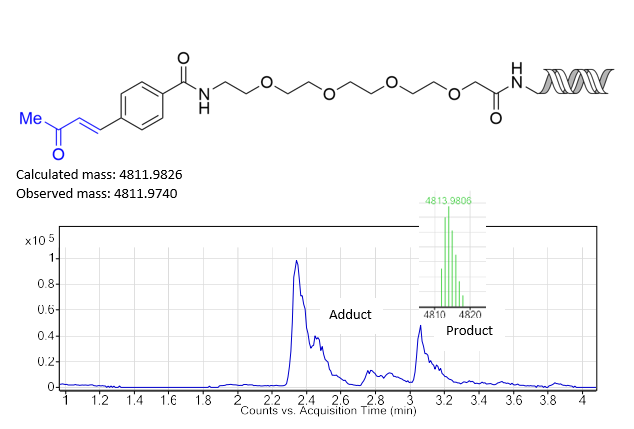

Supplement: Supplementary file 2 — bc3c00051_si_002.zip [file bc3c00051_si_002.zip › Chrom_Spectra/MVKChrom.PNG]
