# Supplementary figures and images for: Development of a Micellar-Promoted Heck Reaction for the Synthesis of DNA-Encoded Libraries (part 1 of 2)
Source: Bioconjug Chem. 2023 Mar 8;34(4):756–63. doi: 10.1021/acs.bioconjchem.3c00051 (PMC10119937; doi:10.1021/acs.bioconjchem.3c00051)

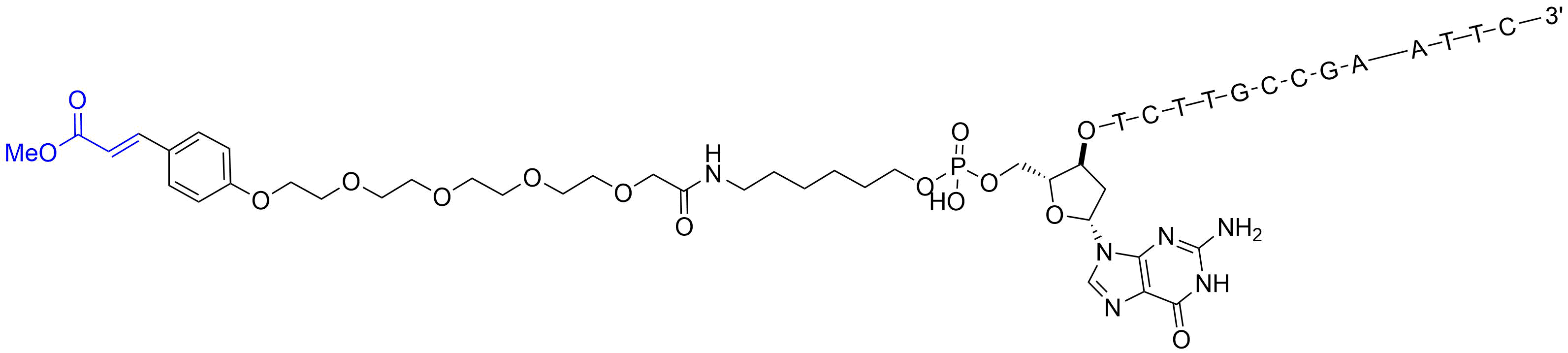

Supplement: Supplementary file 2 — bc3c00051_si_002.zip [file bc3c00051_si_002.zip › Images/DNA40.png]

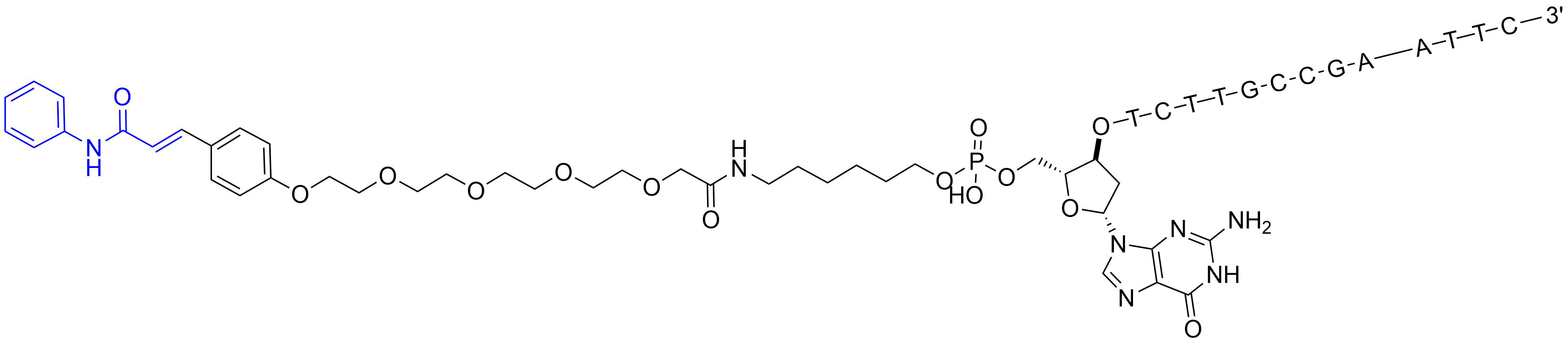

Supplement: Supplementary file 2 — bc3c00051_si_002.zip [file bc3c00051_si_002.zip › Images/DNA45.png]

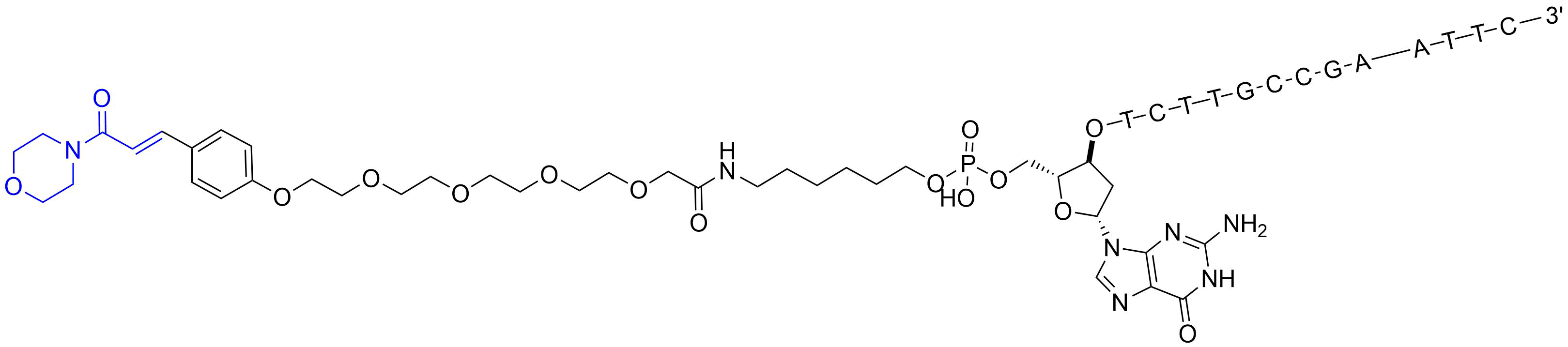

Supplement: Supplementary file 2 — bc3c00051_si_002.zip [file bc3c00051_si_002.zip › Images/DNA46.png]

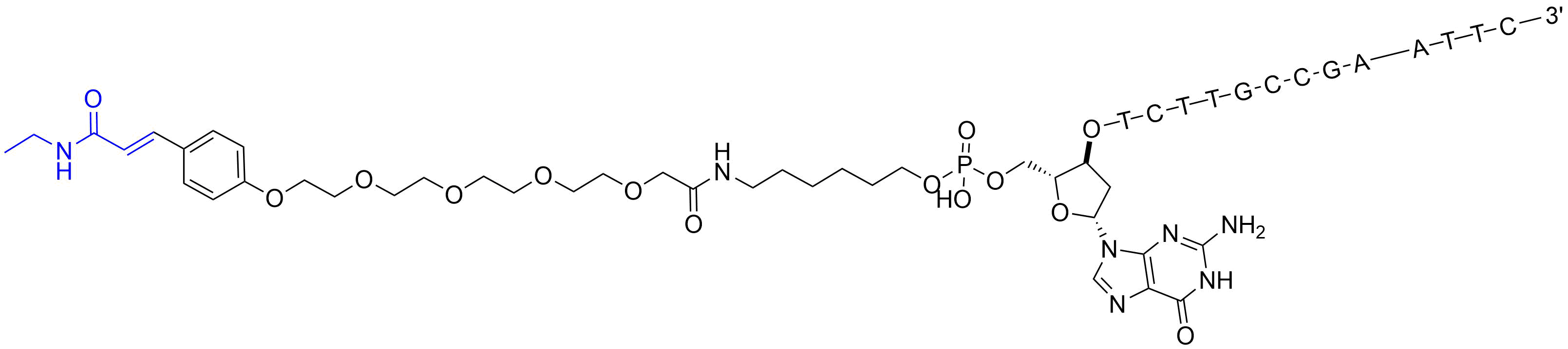

Supplement: Supplementary file 2 — bc3c00051_si_002.zip [file bc3c00051_si_002.zip › Images/DNA47.png]

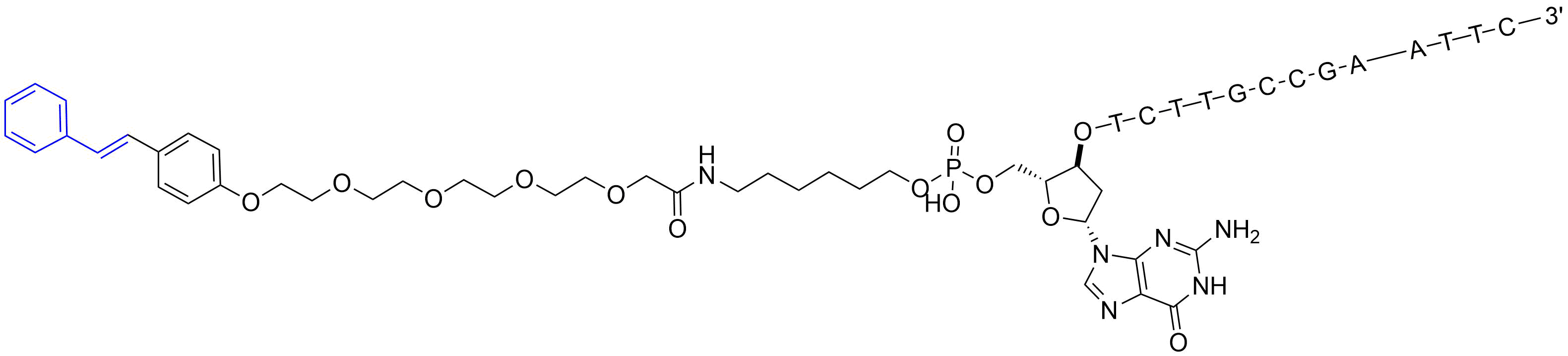

Supplement: Supplementary file 2 — bc3c00051_si_002.zip [file bc3c00051_si_002.zip › Images/DNA48.png]

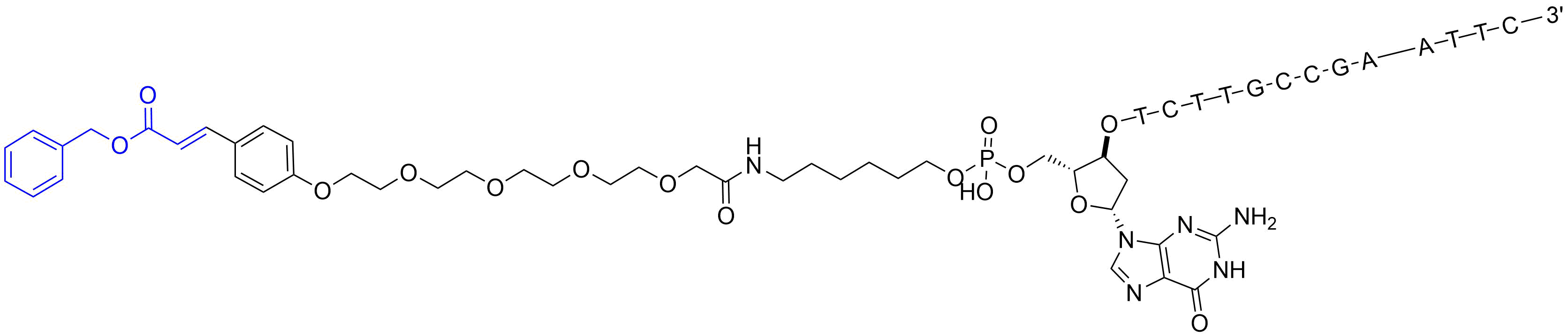

Supplement: Supplementary file 2 — bc3c00051_si_002.zip [file bc3c00051_si_002.zip › Images/DNA49.png]

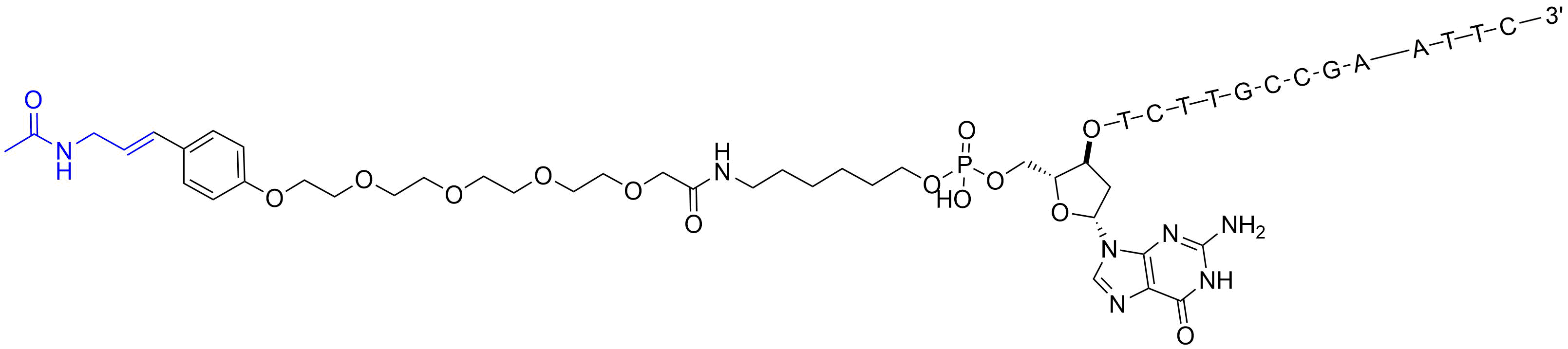

Supplement: Supplementary file 2 — bc3c00051_si_002.zip [file bc3c00051_si_002.zip › Images/DNA50.png]

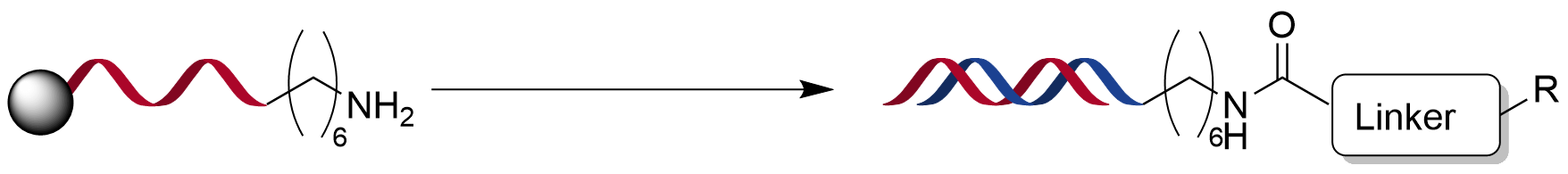

Supplement: Supplementary file 2 — bc3c00051_si_002.zip [file bc3c00051_si_002.zip › Images/HPSynthesisGen.png]

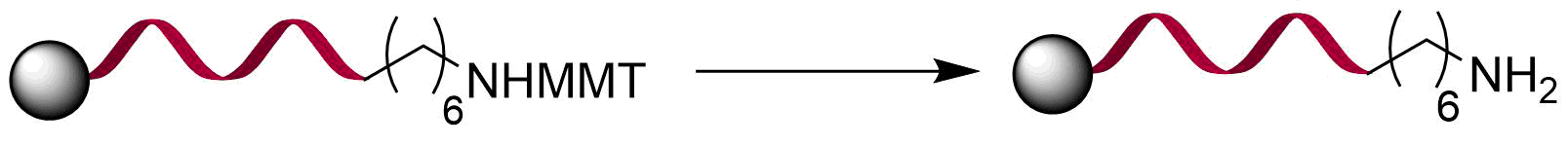

Supplement: Supplementary file 2 — bc3c00051_si_002.zip [file bc3c00051_si_002.zip › Images/MMTDeprotection.png]

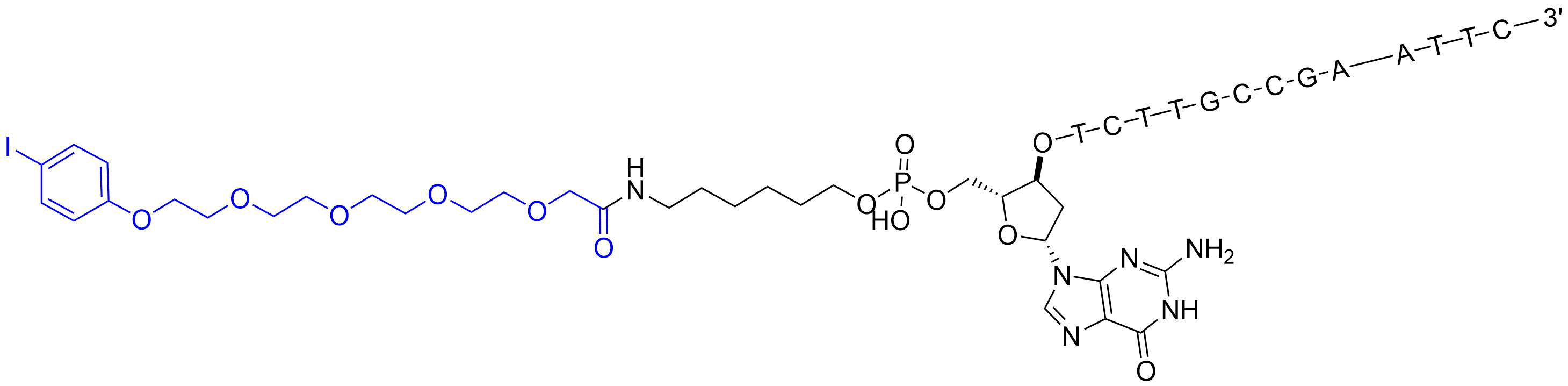

Supplement: Supplementary file 2 — bc3c00051_si_002.zip [file bc3c00051_si_002.zip › Images/IodoHP.png]

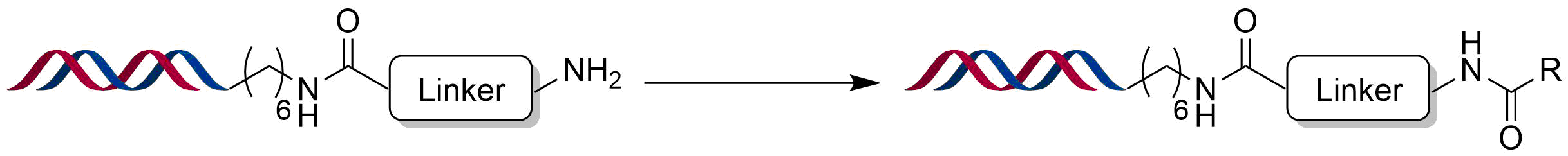

Supplement: Supplementary file 2 — bc3c00051_si_002.zip [file bc3c00051_si_002.zip › Images/Gen4wdAm.png]

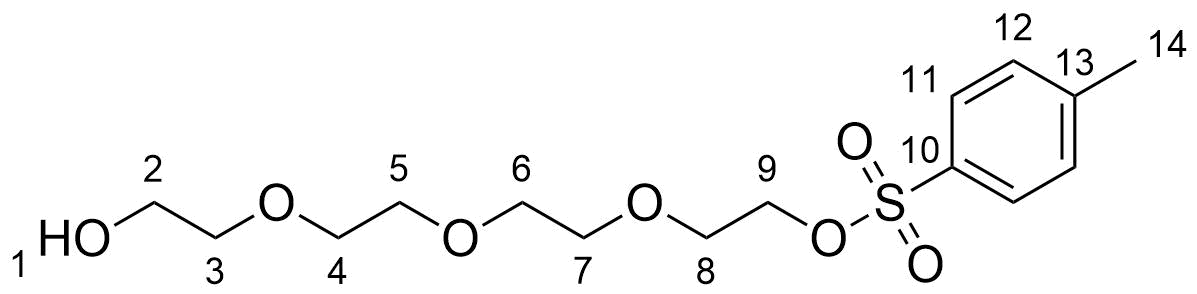

Supplement: Supplementary file 2 — bc3c00051_si_002.zip [file bc3c00051_si_002.zip › Images/Peg4Ts.png]

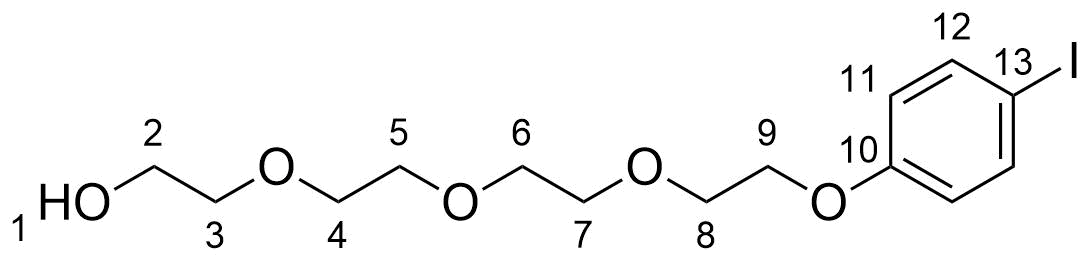

Supplement: Supplementary file 2 — bc3c00051_si_002.zip [file bc3c00051_si_002.zip › Images/Peg4PhI.png]

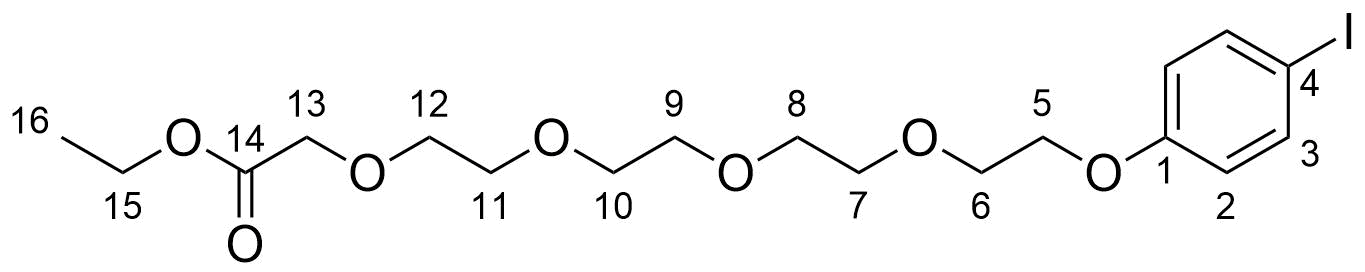

Supplement: Supplementary file 2 — bc3c00051_si_002.zip [file bc3c00051_si_002.zip › Images/esterlinker.png]

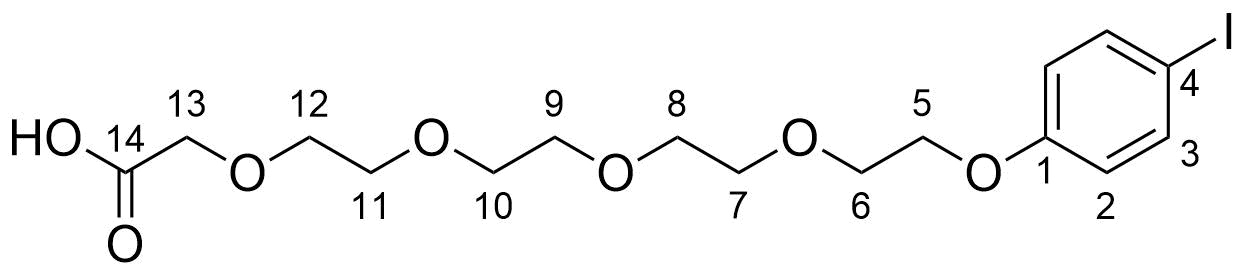

Supplement: Supplementary file 2 — bc3c00051_si_002.zip [file bc3c00051_si_002.zip › Images/acidlinker.png]

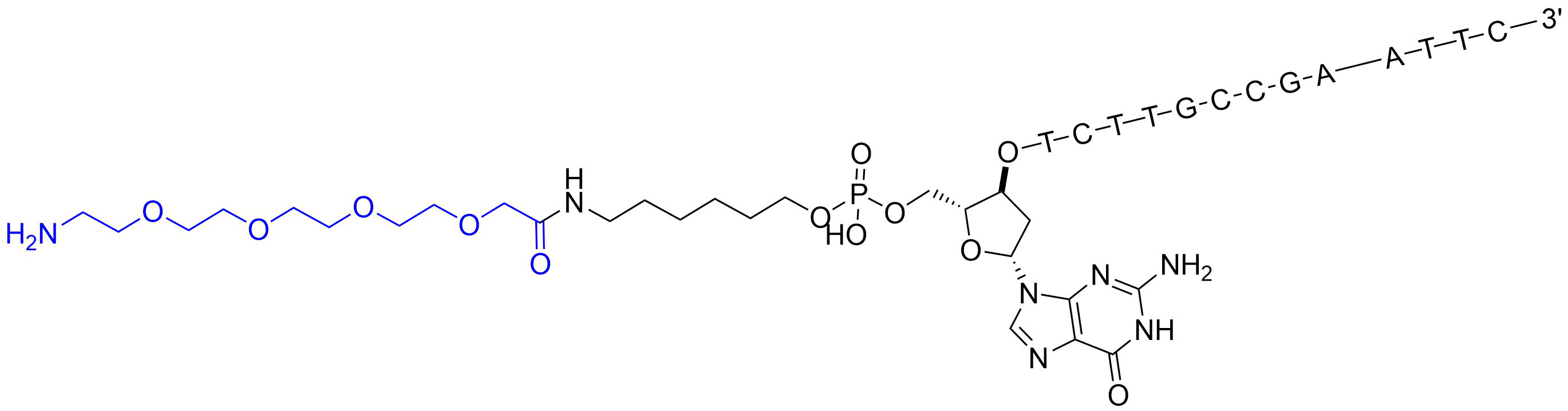

Supplement: Supplementary file 2 — bc3c00051_si_002.zip [file bc3c00051_si_002.zip › Images/AminoHP.png]

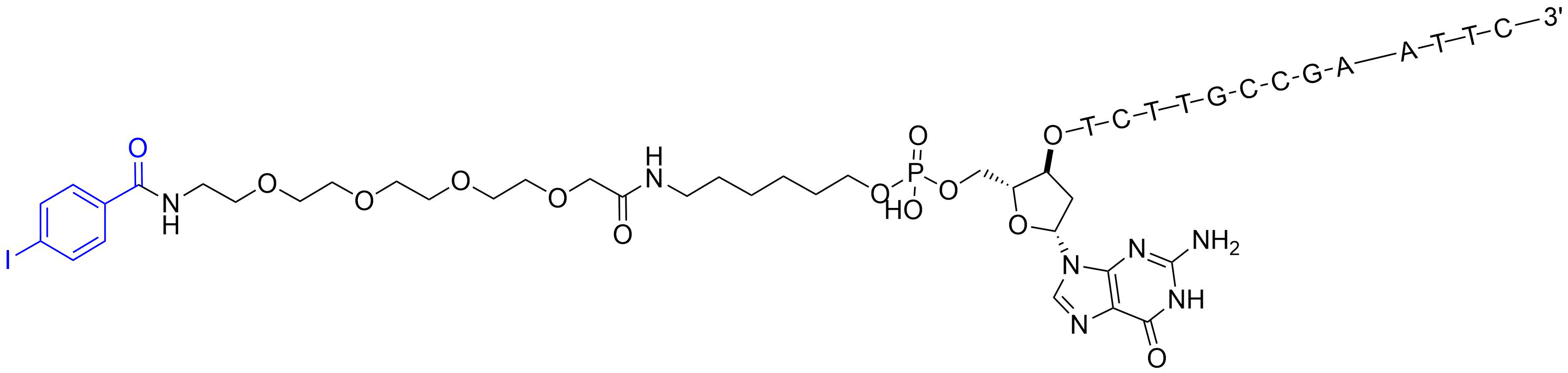

Supplement: Supplementary file 2 — bc3c00051_si_002.zip [file bc3c00051_si_002.zip › Images/DNA52.png]

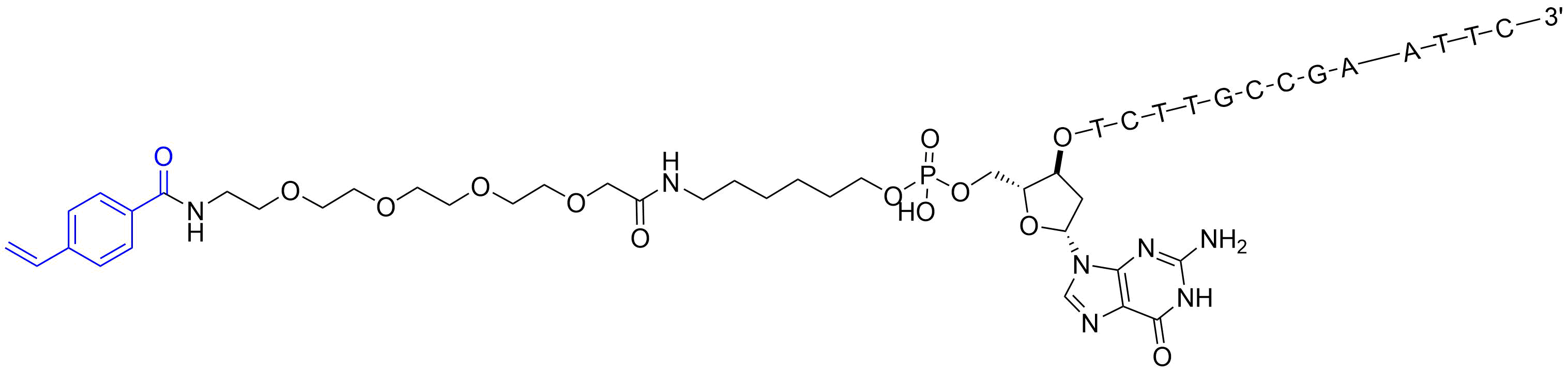

Supplement: Supplementary file 2 — bc3c00051_si_002.zip [file bc3c00051_si_002.zip › Images/DNA71.png]

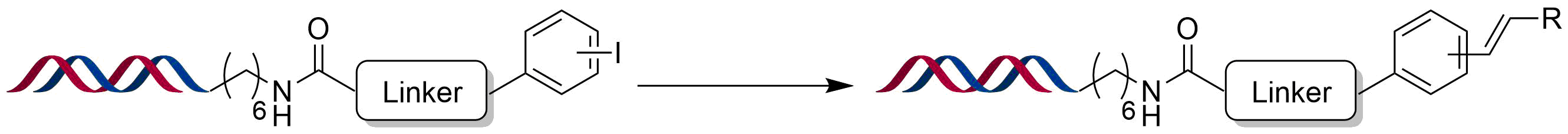

Supplement: Supplementary file 2 — bc3c00051_si_002.zip [file bc3c00051_si_002.zip › Images/GenHeck.png]

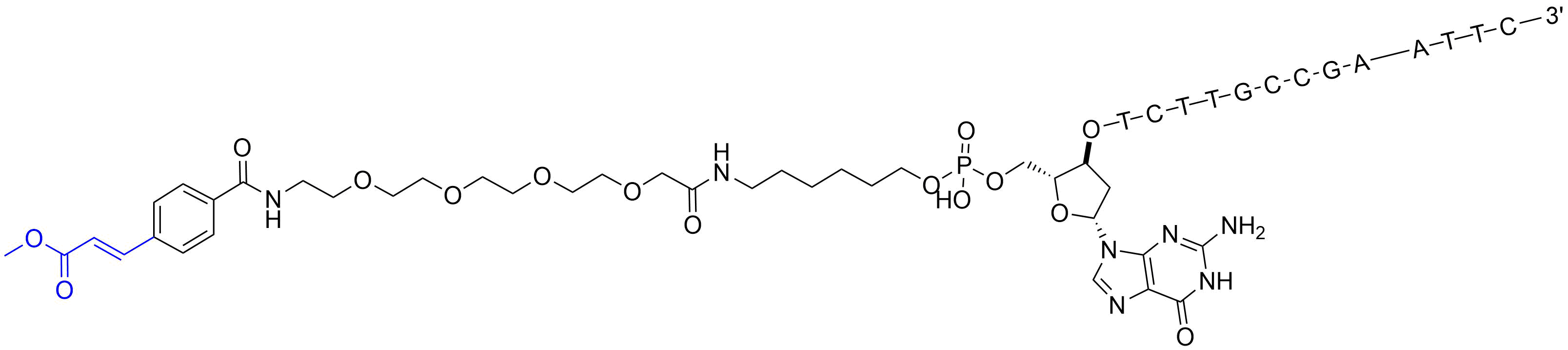

Supplement: Supplementary file 2 — bc3c00051_si_002.zip [file bc3c00051_si_002.zip › Images/DNA54.png]

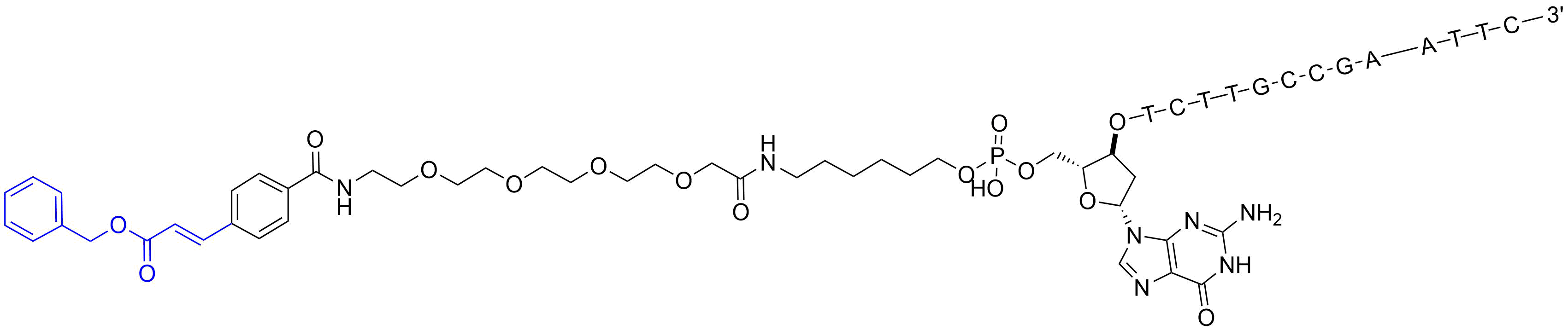

Supplement: Supplementary file 2 — bc3c00051_si_002.zip [file bc3c00051_si_002.zip › Images/DNA55.png]

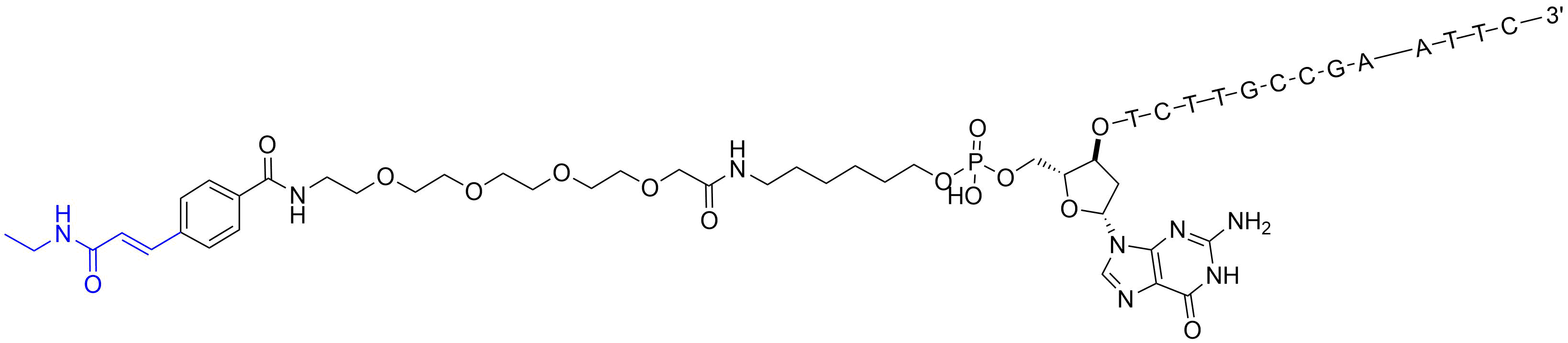

Supplement: Supplementary file 2 — bc3c00051_si_002.zip [file bc3c00051_si_002.zip › Images/DNA56.png]

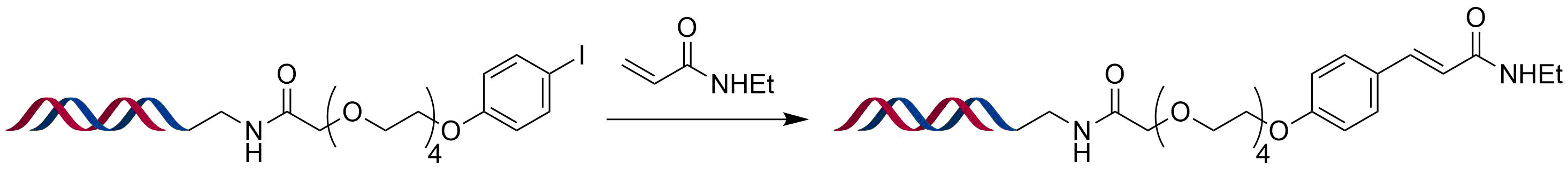

Supplement: Supplementary file 2 — bc3c00051_si_002.zip [file bc3c00051_si_002.zip › Images/SchemeS1.png]

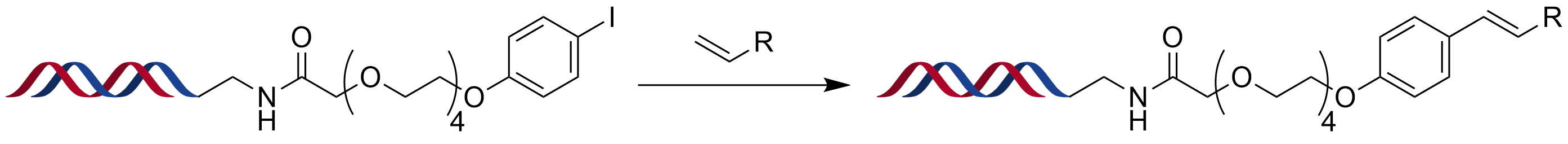

Supplement: Supplementary file 2 — bc3c00051_si_002.zip [file bc3c00051_si_002.zip › Images/SchemeS2.png]

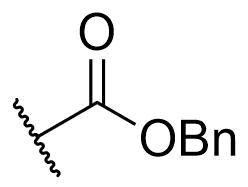

Supplement: Supplementary file 2 — bc3c00051_si_002.zip [file bc3c00051_si_002.zip › Images/Bn_acrylateR.png]

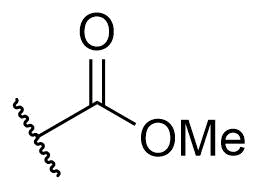

Supplement: Supplementary file 2 — bc3c00051_si_002.zip [file bc3c00051_si_002.zip › Images/Me_acrylateR.png]

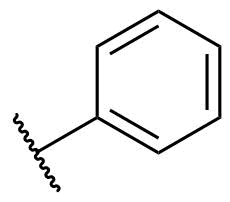

Supplement: Supplementary file 2 — bc3c00051_si_002.zip [file bc3c00051_si_002.zip › Images/StyreneR.png]

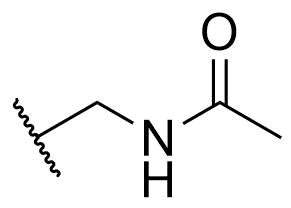

Supplement: Supplementary file 2 — bc3c00051_si_002.zip [file bc3c00051_si_002.zip › Images/N-allyl_acetR.png]

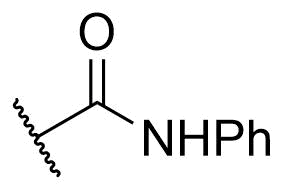

Supplement: Supplementary file 2 — bc3c00051_si_002.zip [file bc3c00051_si_002.zip › Images/N-Ph_acrylR.png]

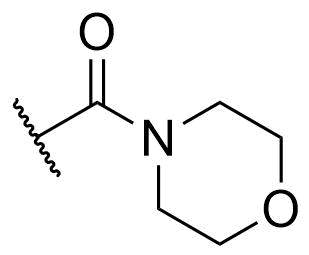

Supplement: Supplementary file 2 — bc3c00051_si_002.zip [file bc3c00051_si_002.zip › Images/N-acryloyl_morphR.png]

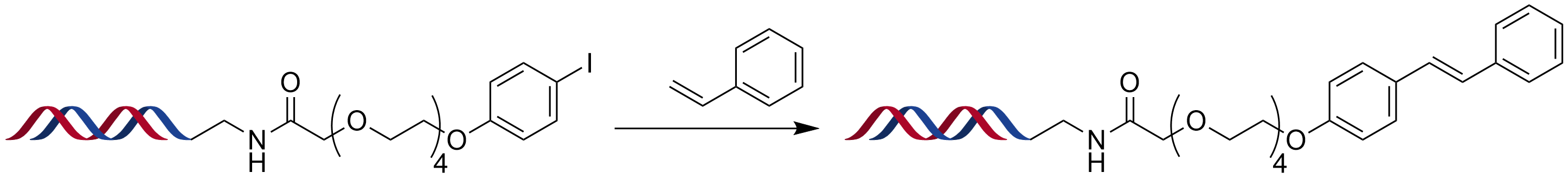

Supplement: Supplementary file 2 — bc3c00051_si_002.zip [file bc3c00051_si_002.zip › Images/SchemeS3.png]

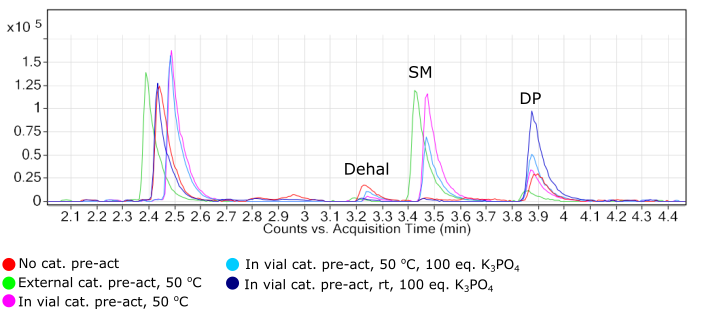

Supplement: Supplementary file 2 — bc3c00051_si_002.zip [file bc3c00051_si_002.zip › Images/OverlayCatPA.png]

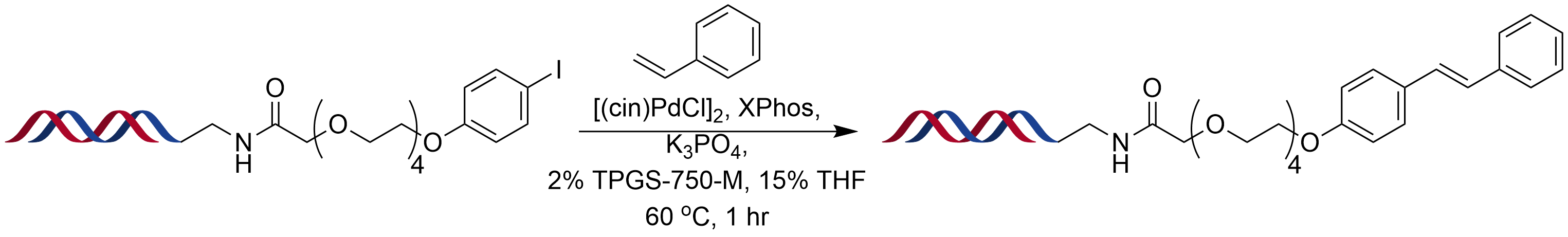

Supplement: Supplementary file 2 — bc3c00051_si_002.zip [file bc3c00051_si_002.zip › Images/SchemeS4.png]

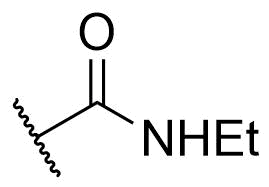

Supplement: Supplementary file 2 — bc3c00051_si_002.zip [file bc3c00051_si_002.zip › Images/N-Et_acrylR.png]

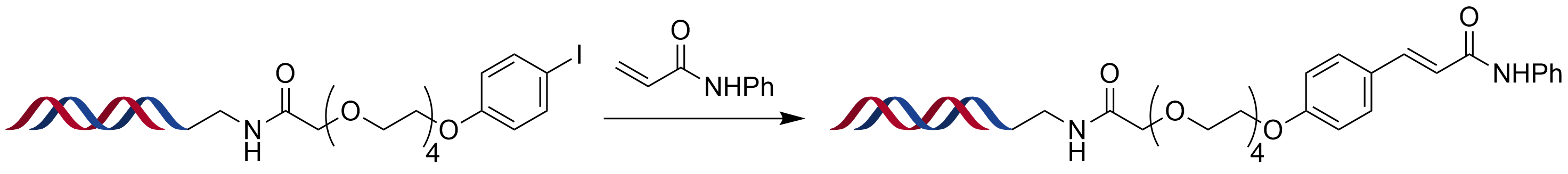

Supplement: Supplementary file 2 — bc3c00051_si_002.zip [file bc3c00051_si_002.zip › Images/SchemeS5.png]

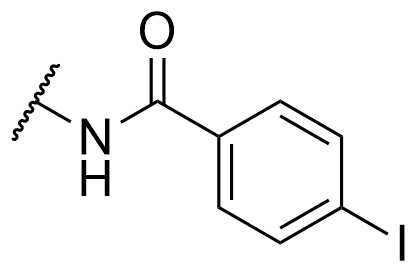

Supplement: Supplementary file 2 — bc3c00051_si_002.zip [file bc3c00051_si_002.zip › Images/IPhAmide.png]

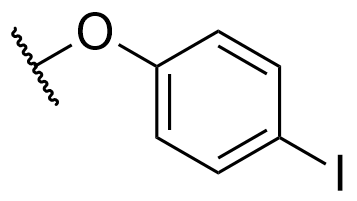

Supplement: Supplementary file 2 — bc3c00051_si_002.zip [file bc3c00051_si_002.zip › Images/IPhEther.png]

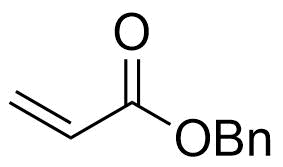

Supplement: Supplementary file 2 — bc3c00051_si_002.zip [file bc3c00051_si_002.zip › Images/Bn_acrylate.png]

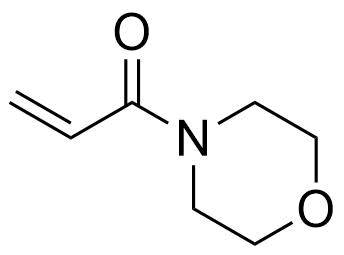

Supplement: Supplementary file 2 — bc3c00051_si_002.zip [file bc3c00051_si_002.zip › Images/N-acryloyl_morph.png]

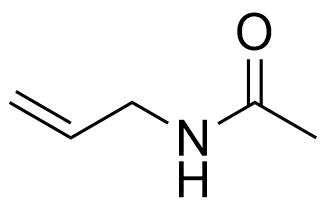

Supplement: Supplementary file 2 — bc3c00051_si_002.zip [file bc3c00051_si_002.zip › Images/N-allyl_acet.png]

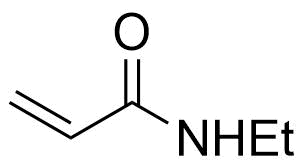

Supplement: Supplementary file 2 — bc3c00051_si_002.zip [file bc3c00051_si_002.zip › Images/N-Et_acryl.png]

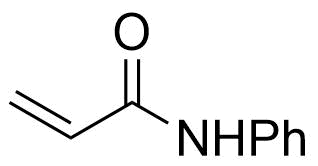

Supplement: Supplementary file 2 — bc3c00051_si_002.zip [file bc3c00051_si_002.zip › Images/N-Ph_acryl.png]

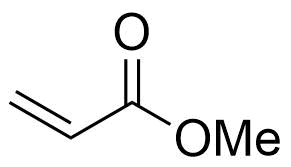

Supplement: Supplementary file 2 — bc3c00051_si_002.zip [file bc3c00051_si_002.zip › Images/Me_acrylate.png]

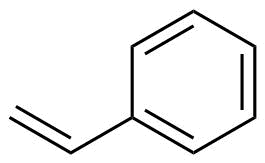

Supplement: Supplementary file 2 — bc3c00051_si_002.zip [file bc3c00051_si_002.zip › Images/Styrene.png]

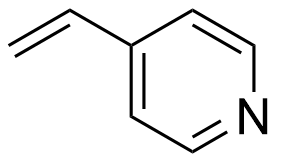

Supplement: Supplementary file 2 — bc3c00051_si_002.zip [file bc3c00051_si_002.zip › Images/4pyridyl.png]

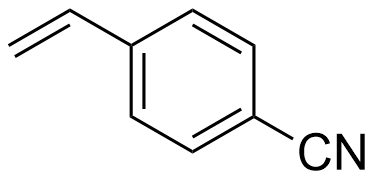

Supplement: Supplementary file 2 — bc3c00051_si_002.zip [file bc3c00051_si_002.zip › Images/nitrile.png]

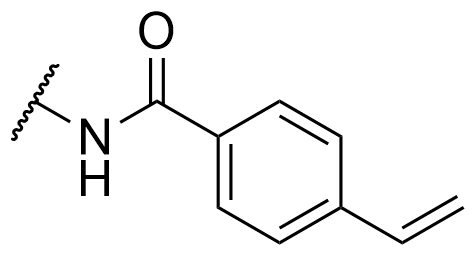

Supplement: Supplementary file 2 — bc3c00051_si_002.zip [file bc3c00051_si_002.zip › Images/StyrylHP.png]

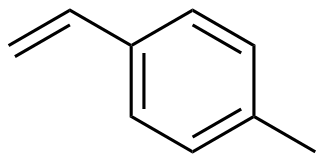

Supplement: Supplementary file 2 — bc3c00051_si_002.zip [file bc3c00051_si_002.zip › Images/Tolyl.png]

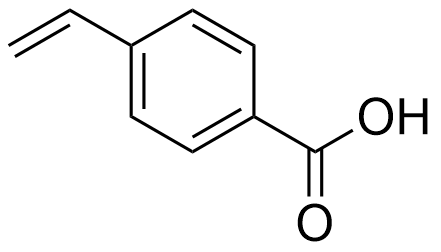

Supplement: Supplementary file 2 — bc3c00051_si_002.zip [file bc3c00051_si_002.zip › Images/vinylbenzoicacid.png]

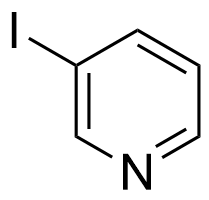

Supplement: Supplementary file 2 — bc3c00051_si_002.zip [file bc3c00051_si_002.zip › Images/3pyridyl.png]

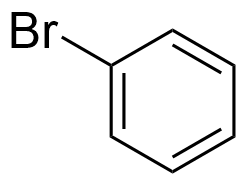

Supplement: Supplementary file 2 — bc3c00051_si_002.zip [file bc3c00051_si_002.zip › Images/Brbenzene.png]

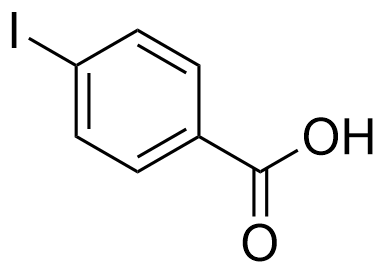

Supplement: Supplementary file 2 — bc3c00051_si_002.zip [file bc3c00051_si_002.zip › Images/iodoacid.png]

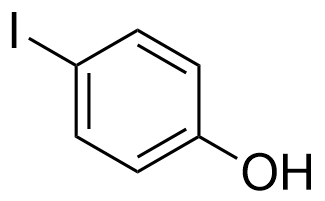

Supplement: Supplementary file 2 — bc3c00051_si_002.zip [file bc3c00051_si_002.zip › Images/iodophenol.png]

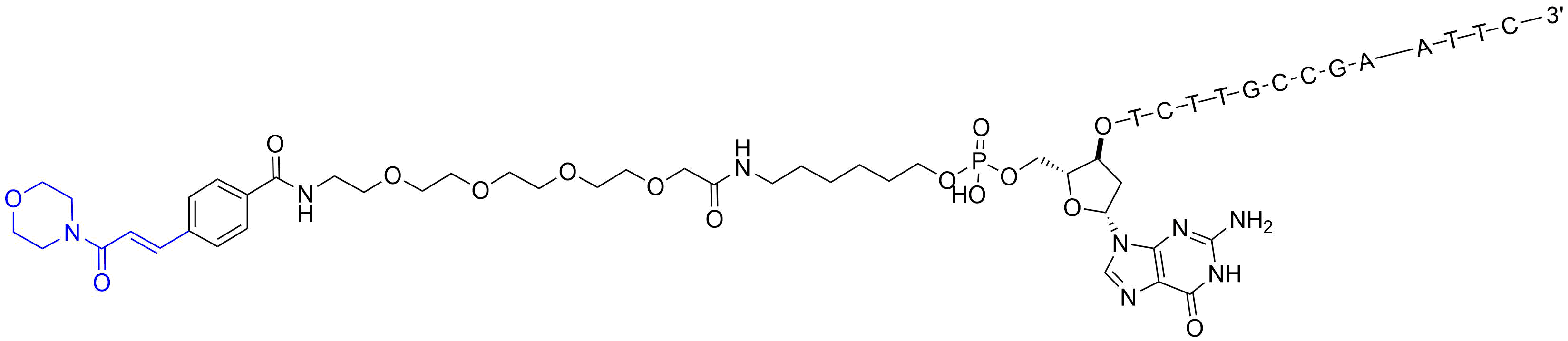

Supplement: Supplementary file 2 — bc3c00051_si_002.zip [file bc3c00051_si_002.zip › Images/DNA57.png]

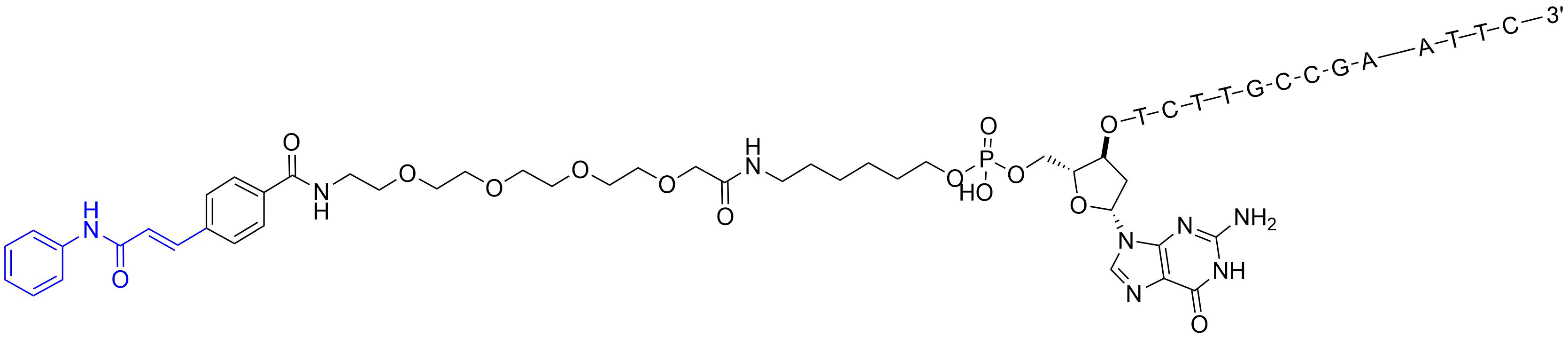

Supplement: Supplementary file 2 — bc3c00051_si_002.zip [file bc3c00051_si_002.zip › Images/DNA58.png]

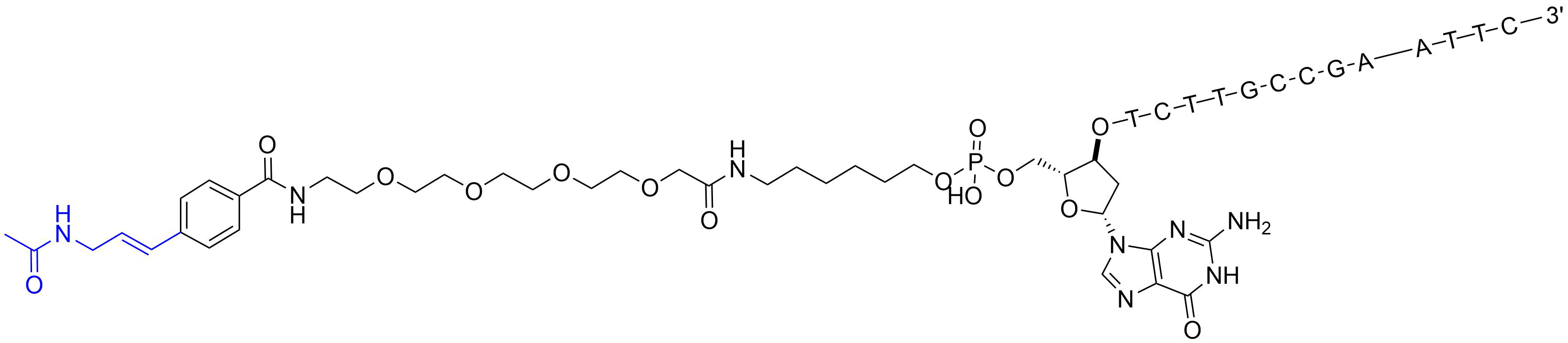

Supplement: Supplementary file 2 — bc3c00051_si_002.zip [file bc3c00051_si_002.zip › Images/DNA59.png]

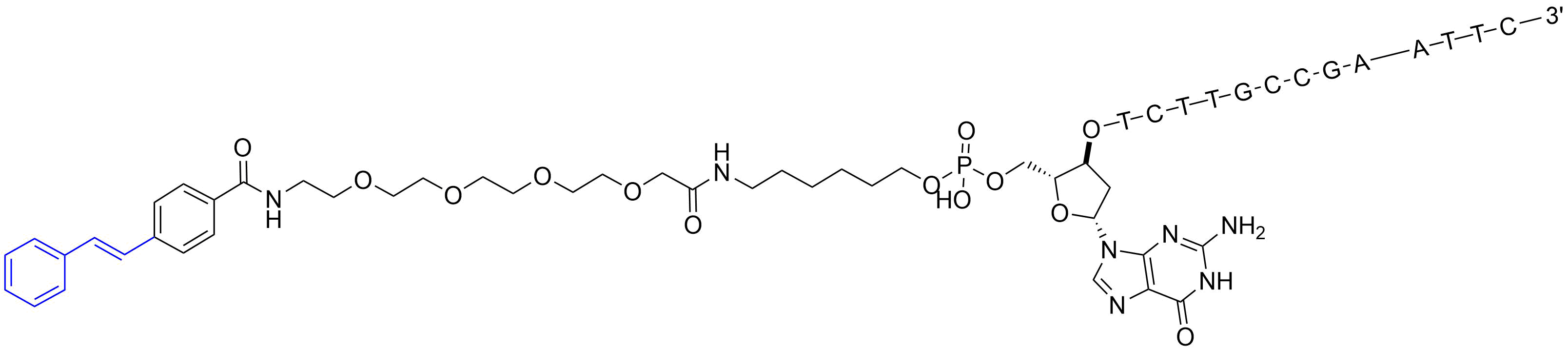

Supplement: Supplementary file 2 — bc3c00051_si_002.zip [file bc3c00051_si_002.zip › Images/DNA60.png]

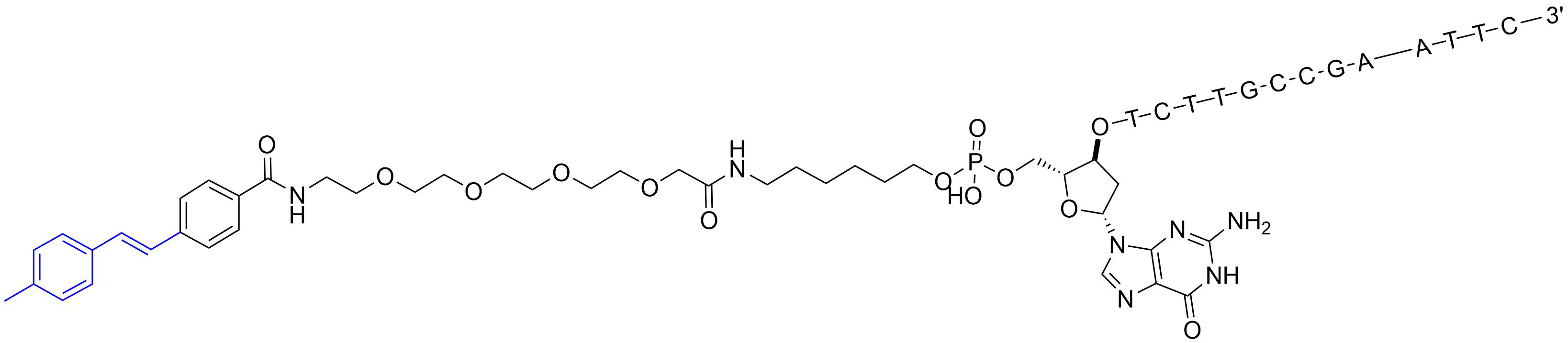

Supplement: Supplementary file 2 — bc3c00051_si_002.zip [file bc3c00051_si_002.zip › Images/DNA61.png]

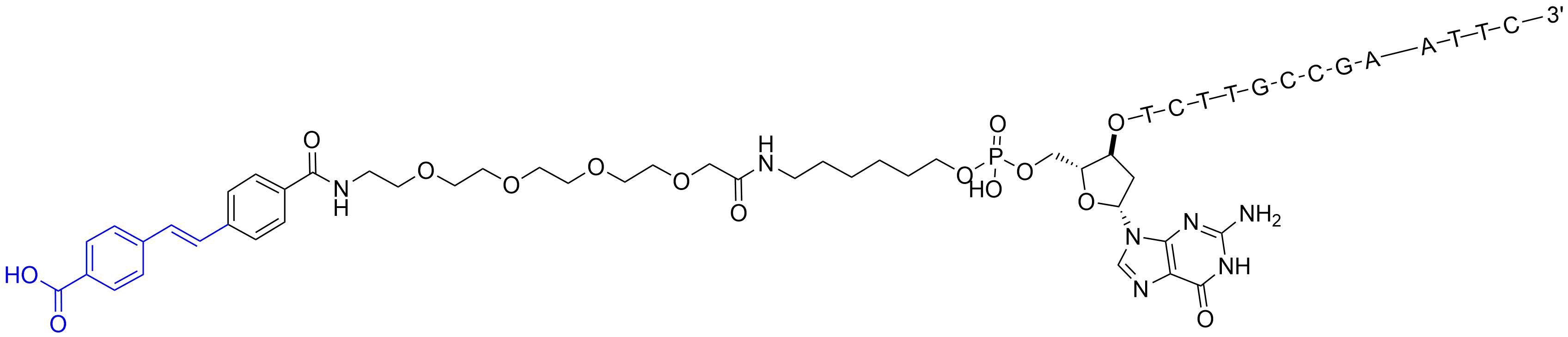

Supplement: Supplementary file 2 — bc3c00051_si_002.zip [file bc3c00051_si_002.zip › Images/DNA62.png]

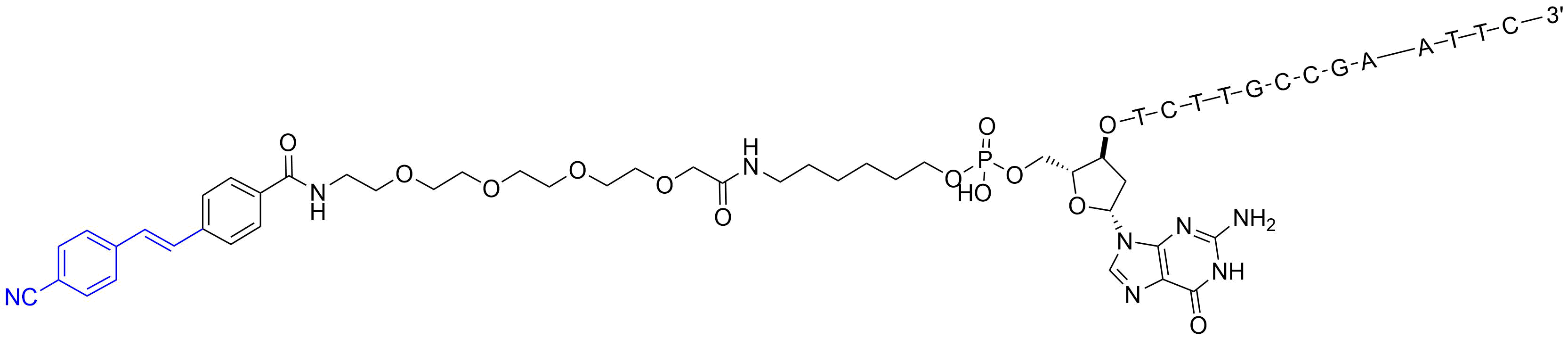

Supplement: Supplementary file 2 — bc3c00051_si_002.zip [file bc3c00051_si_002.zip › Images/DNA63.png]

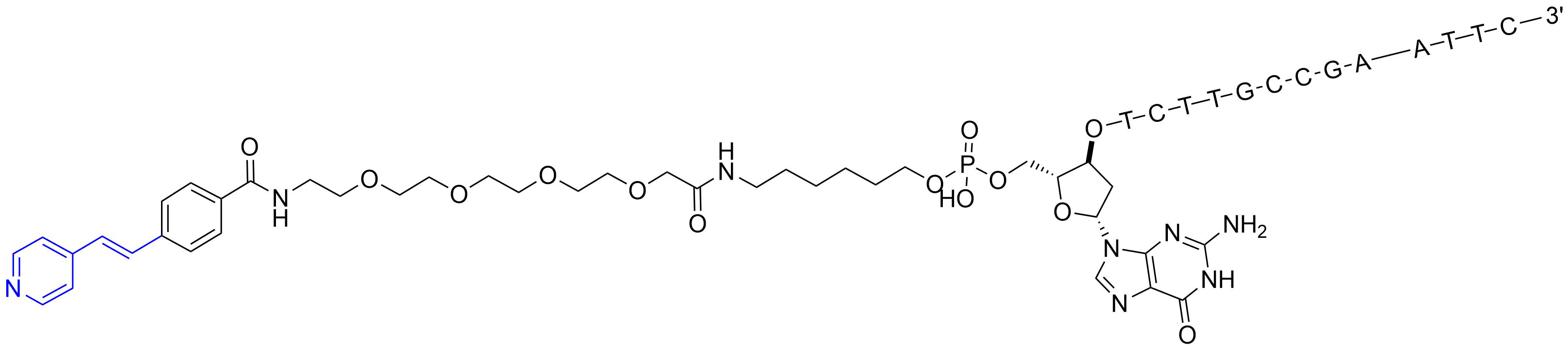

Supplement: Supplementary file 2 — bc3c00051_si_002.zip [file bc3c00051_si_002.zip › Images/DNA64.png]

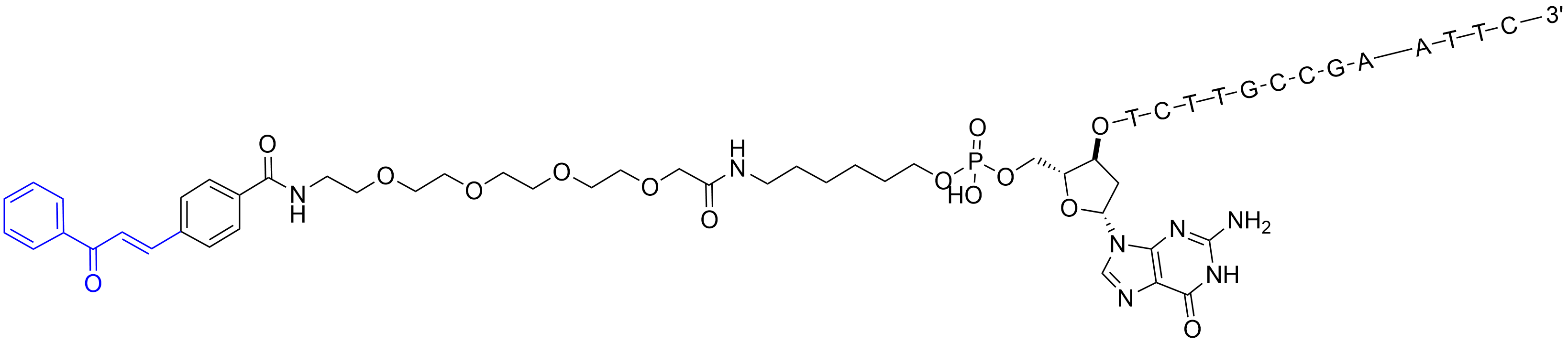

Supplement: Supplementary file 2 — bc3c00051_si_002.zip [file bc3c00051_si_002.zip › Images/DNAacrylophenone.png]

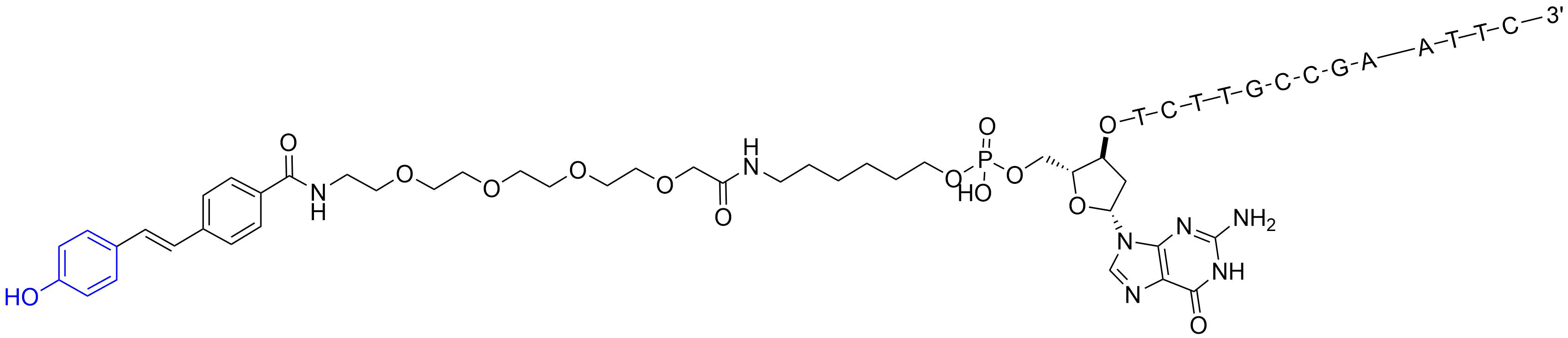

Supplement: Supplementary file 2 — bc3c00051_si_002.zip [file bc3c00051_si_002.zip › Images/DNA74.png]

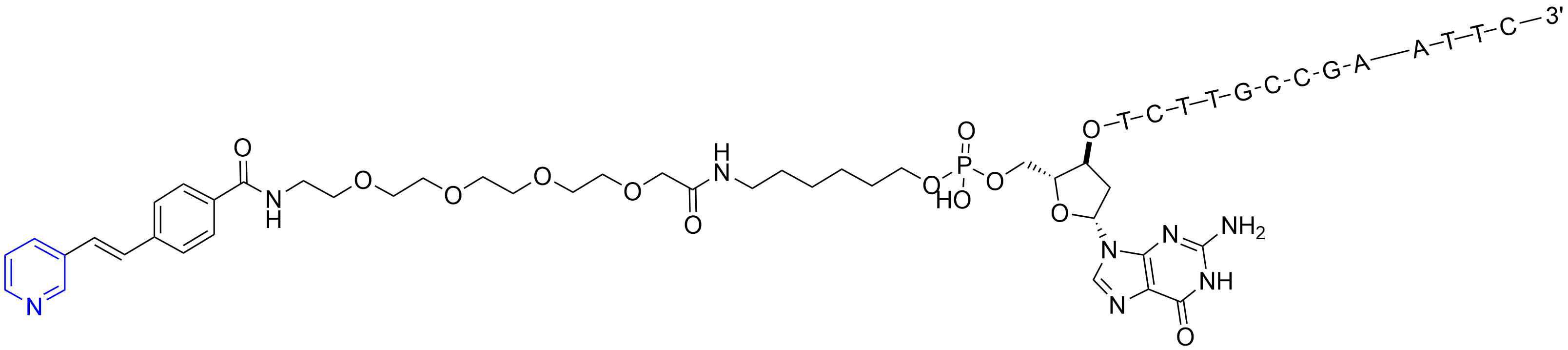

Supplement: Supplementary file 2 — bc3c00051_si_002.zip [file bc3c00051_si_002.zip › Images/DNA75.png]

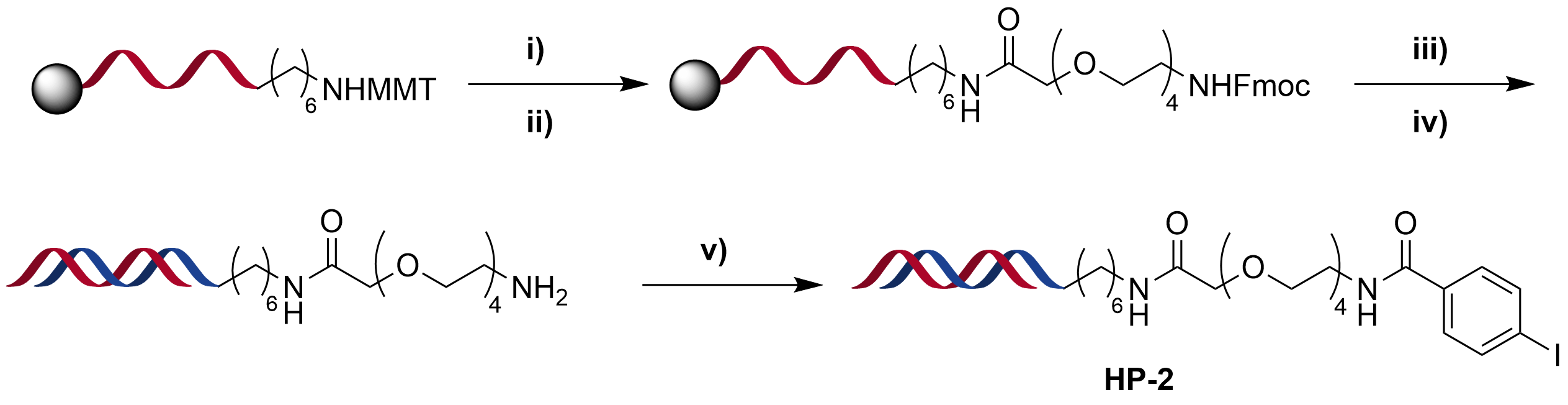

Supplement: Supplementary file 2 — bc3c00051_si_002.zip [file bc3c00051_si_002.zip › Images/HP2_Synthesis.png]

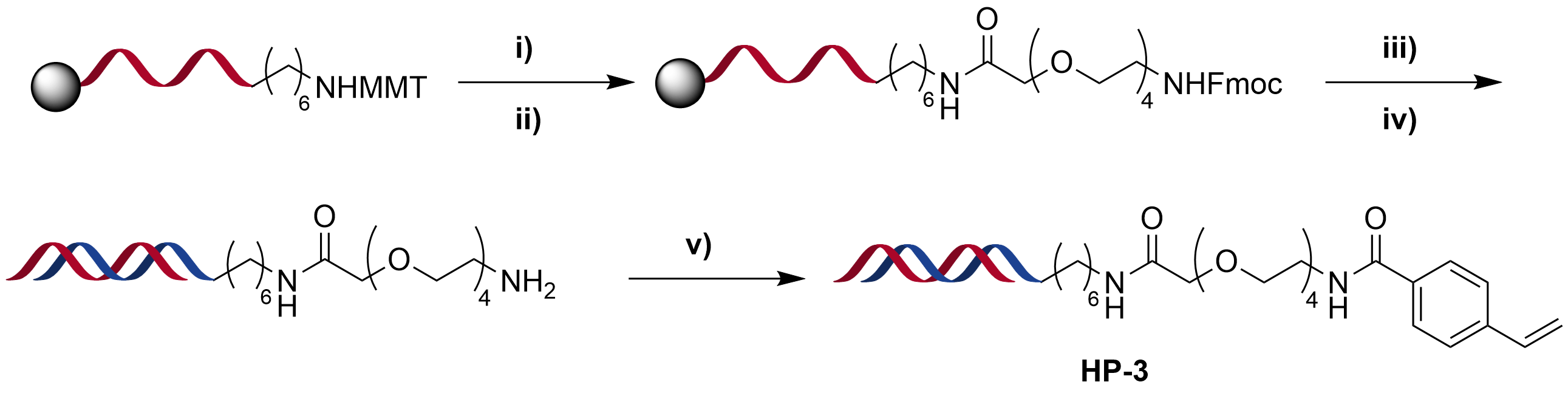

Supplement: Supplementary file 2 — bc3c00051_si_002.zip [file bc3c00051_si_002.zip › Images/HP3_Synthesis.png]

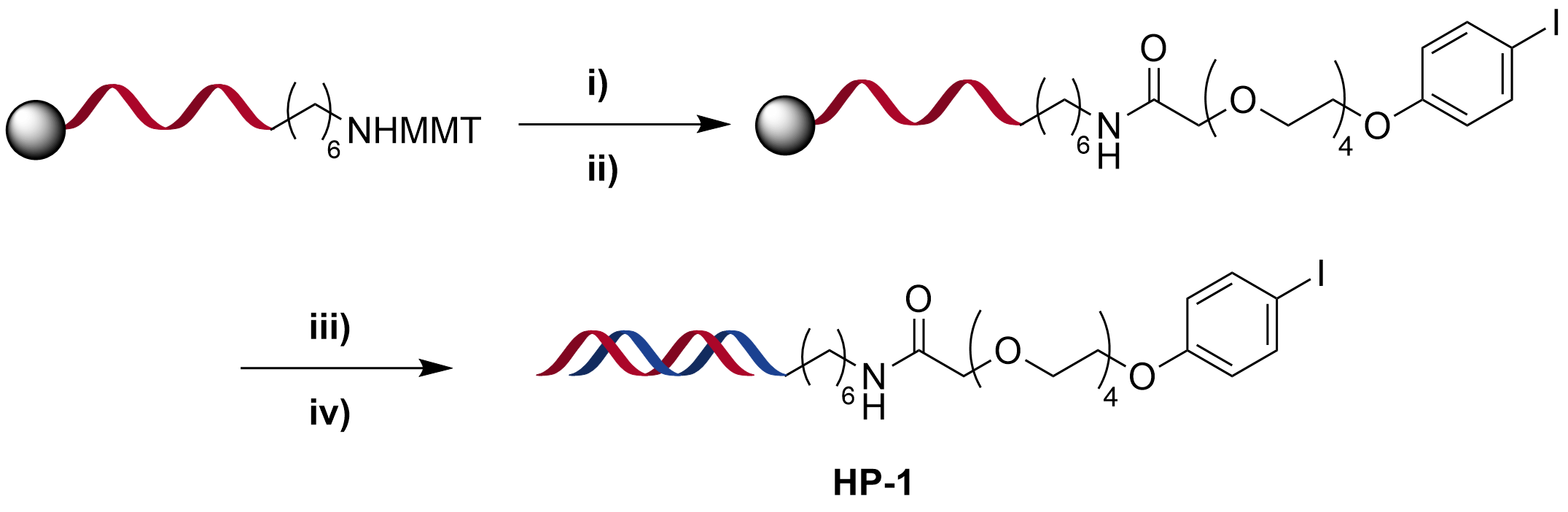

Supplement: Supplementary file 2 — bc3c00051_si_002.zip [file bc3c00051_si_002.zip › Images/HP1_Synthesis.png]

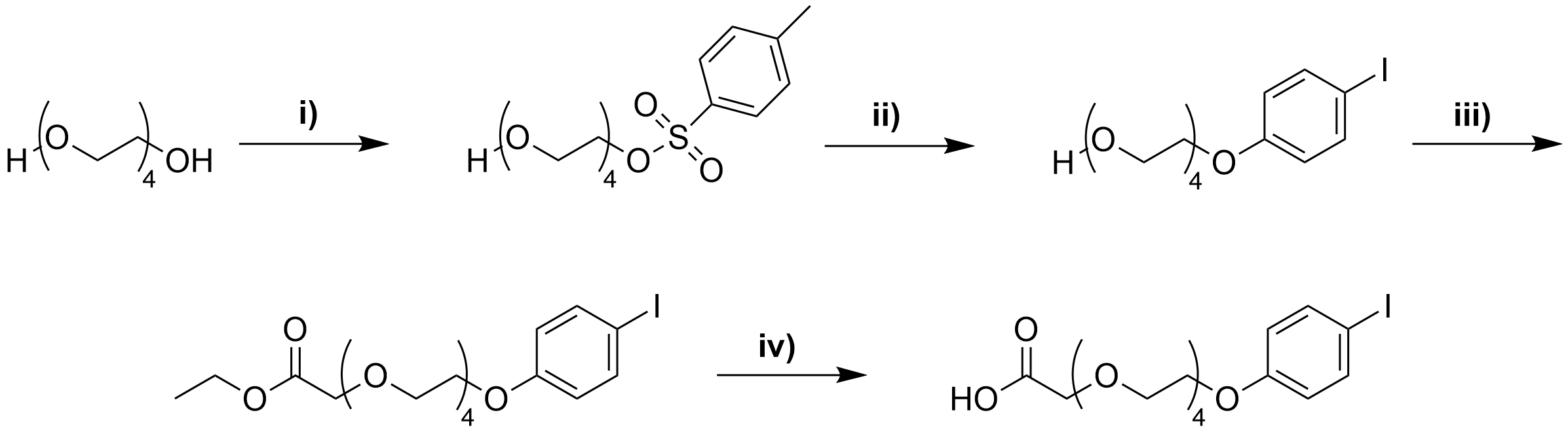

Supplement: Supplementary file 2 — bc3c00051_si_002.zip [file bc3c00051_si_002.zip › Images/Linker_Synthesis.png]

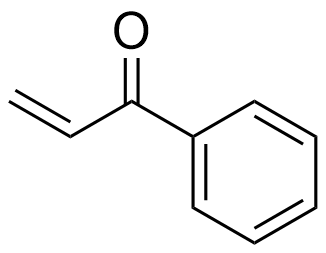

Supplement: Supplementary file 2 — bc3c00051_si_002.zip [file bc3c00051_si_002.zip › Images/acrylophenone.png]

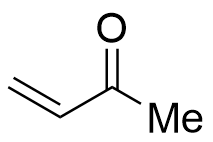

Supplement: Supplementary file 2 — bc3c00051_si_002.zip [file bc3c00051_si_002.zip › Images/MVK.png]

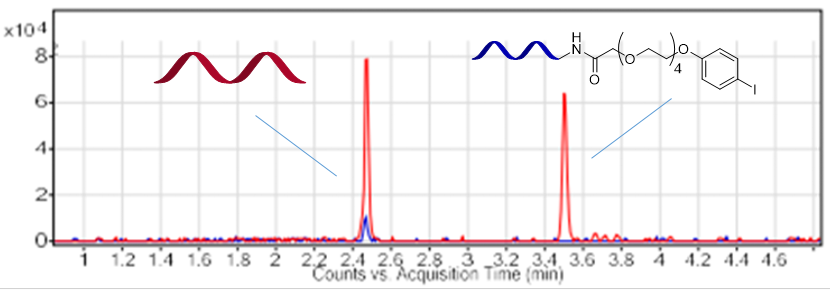

Supplement: Supplementary file 2 — bc3c00051_si_002.zip [file bc3c00051_si_002.zip › Images/Pd0PdII.png]

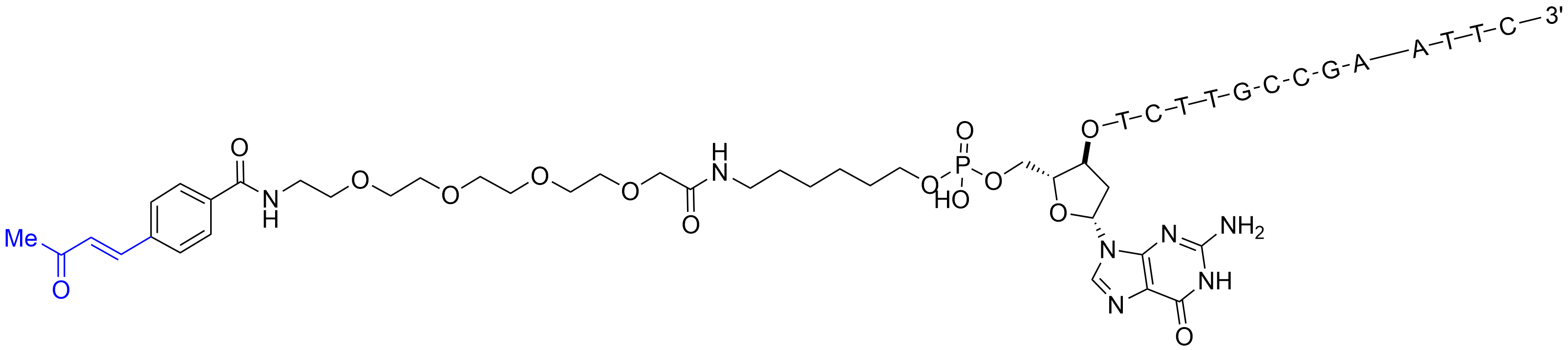

Supplement: Supplementary file 2 — bc3c00051_si_002.zip [file bc3c00051_si_002.zip › Images/JakeMVKoligo.png]

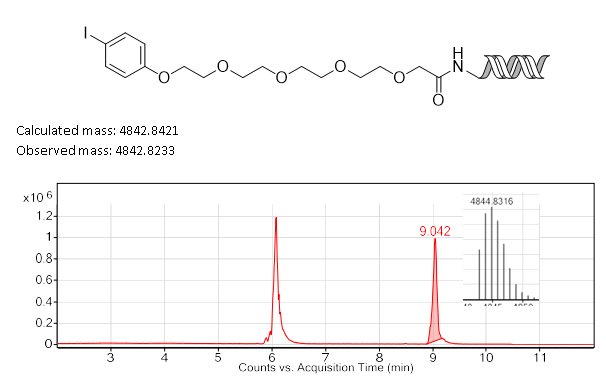

Supplement: Supplementary file 2 — bc3c00051_si_002.zip [file bc3c00051_si_002.zip › Chrom_Spectra/HP39.PNG]

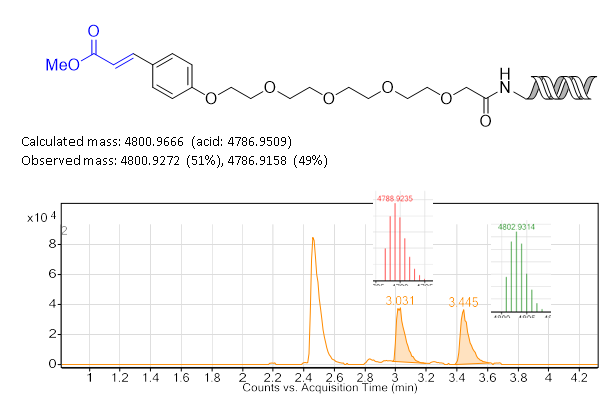

Supplement: Supplementary file 2 — bc3c00051_si_002.zip [file bc3c00051_si_002.zip › Chrom_Spectra/A40.PNG]

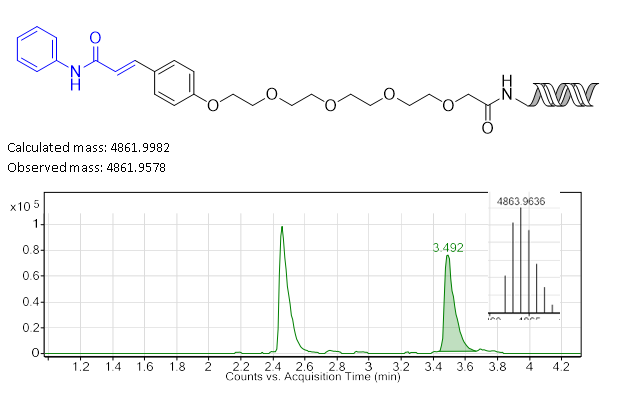

Supplement: Supplementary file 2 — bc3c00051_si_002.zip [file bc3c00051_si_002.zip › Chrom_Spectra/A45.PNG]

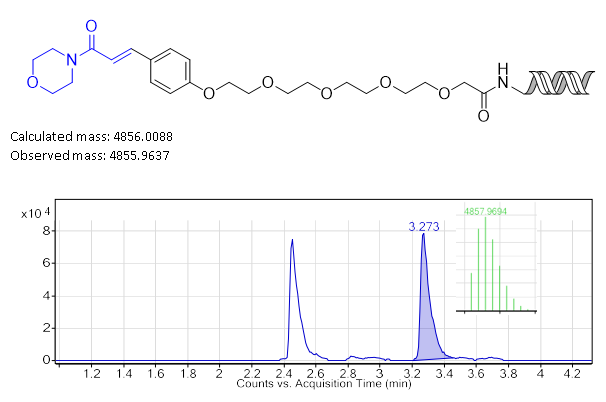

Supplement: Supplementary file 2 — bc3c00051_si_002.zip [file bc3c00051_si_002.zip › Chrom_Spectra/A46.PNG]

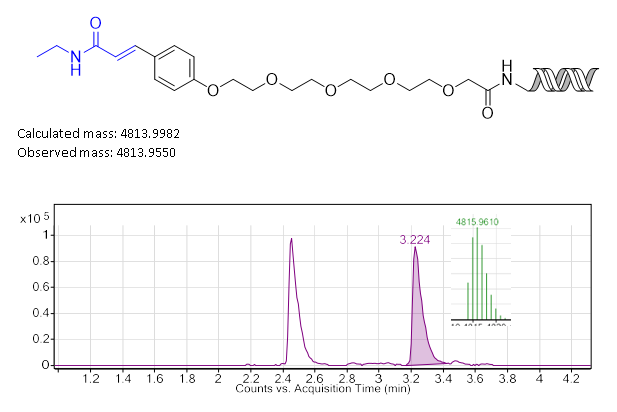

Supplement: Supplementary file 2 — bc3c00051_si_002.zip [file bc3c00051_si_002.zip › Chrom_Spectra/A47.PNG]

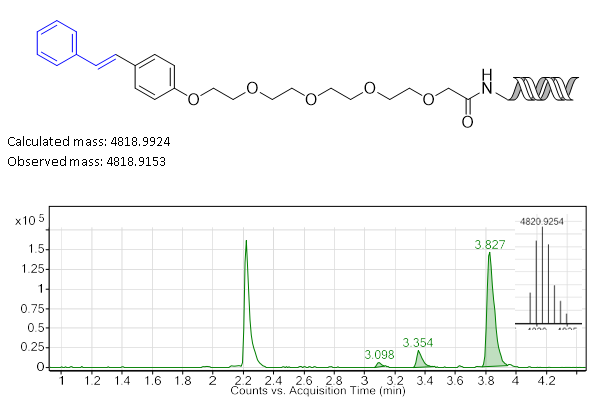

Supplement: Supplementary file 2 — bc3c00051_si_002.zip [file bc3c00051_si_002.zip › Chrom_Spectra/A48.PNG]

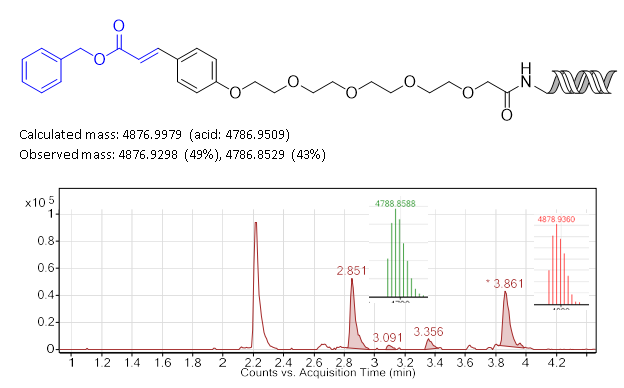

Supplement: Supplementary file 2 — bc3c00051_si_002.zip [file bc3c00051_si_002.zip › Chrom_Spectra/A49.PNG]

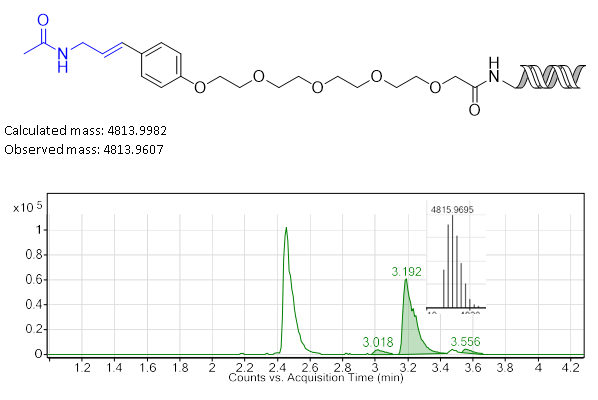

Supplement: Supplementary file 2 — bc3c00051_si_002.zip [file bc3c00051_si_002.zip › Chrom_Spectra/A50.PNG]

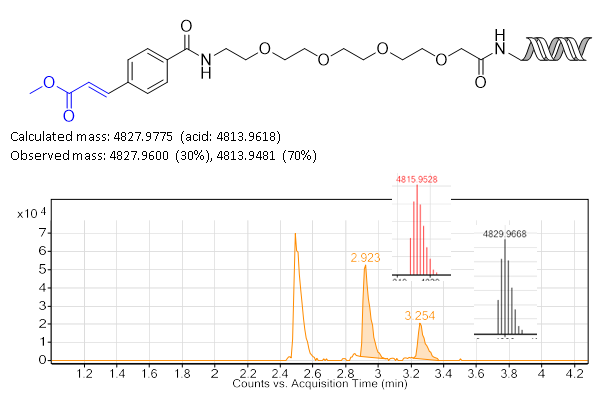

Supplement: Supplementary file 2 — bc3c00051_si_002.zip [file bc3c00051_si_002.zip › Chrom_Spectra/A54.PNG]

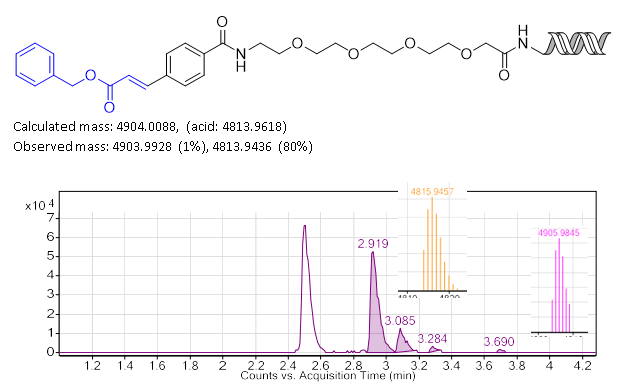

Supplement: Supplementary file 2 — bc3c00051_si_002.zip [file bc3c00051_si_002.zip › Chrom_Spectra/A55.PNG]

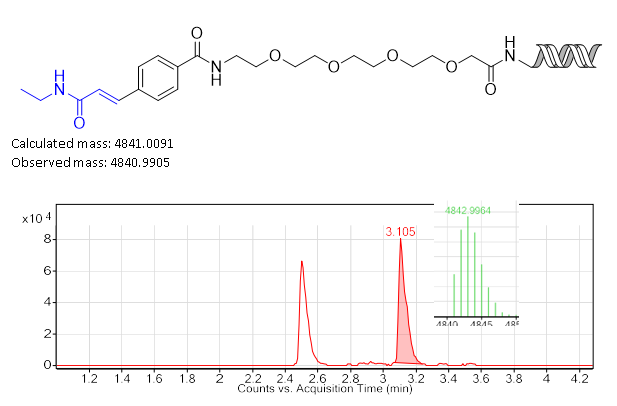

Supplement: Supplementary file 2 — bc3c00051_si_002.zip [file bc3c00051_si_002.zip › Chrom_Spectra/A56.PNG]

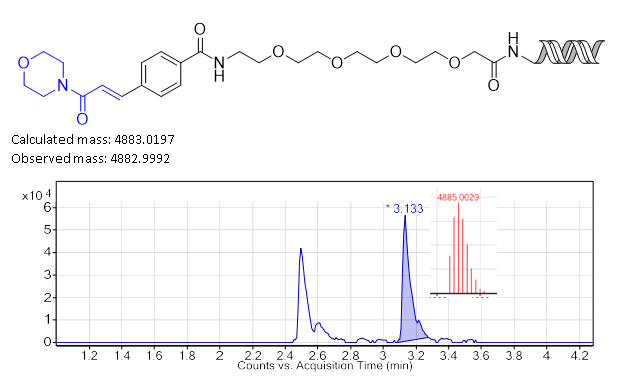

Supplement: Supplementary file 2 — bc3c00051_si_002.zip [file bc3c00051_si_002.zip › Chrom_Spectra/A57.PNG]

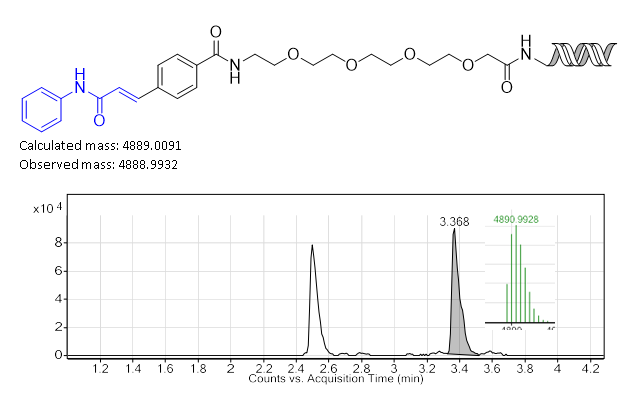

Supplement: Supplementary file 2 — bc3c00051_si_002.zip [file bc3c00051_si_002.zip › Chrom_Spectra/A58.PNG]

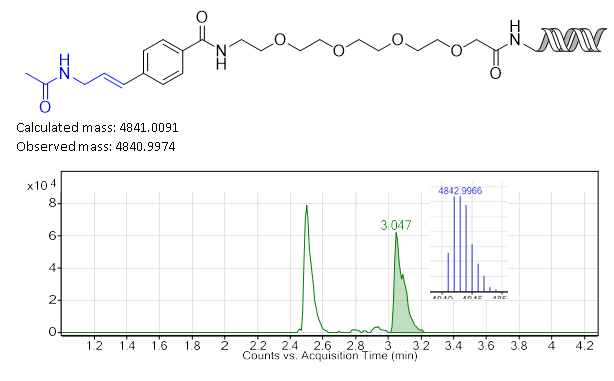

Supplement: Supplementary file 2 — bc3c00051_si_002.zip [file bc3c00051_si_002.zip › Chrom_Spectra/A59.PNG]

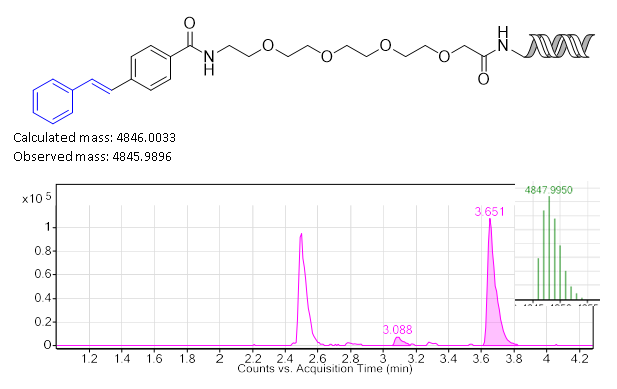

Supplement: Supplementary file 2 — bc3c00051_si_002.zip [file bc3c00051_si_002.zip › Chrom_Spectra/A60.PNG]

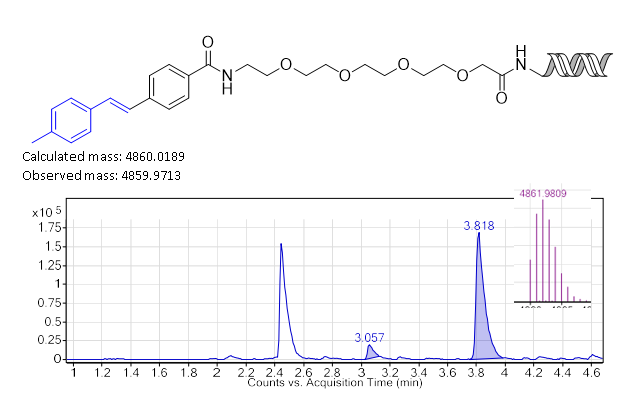

Supplement: Supplementary file 2 — bc3c00051_si_002.zip [file bc3c00051_si_002.zip › Chrom_Spectra/A61.PNG]

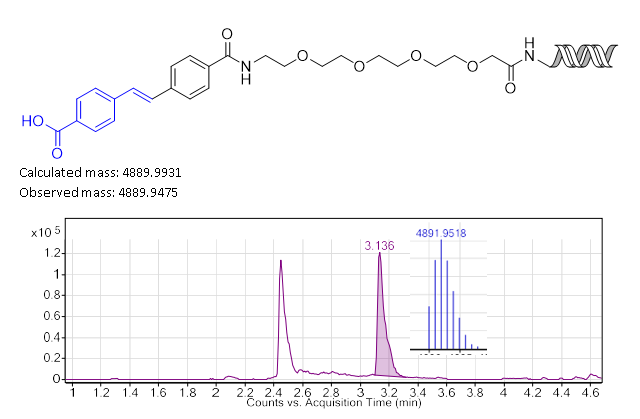

Supplement: Supplementary file 2 — bc3c00051_si_002.zip [file bc3c00051_si_002.zip › Chrom_Spectra/A62.PNG]

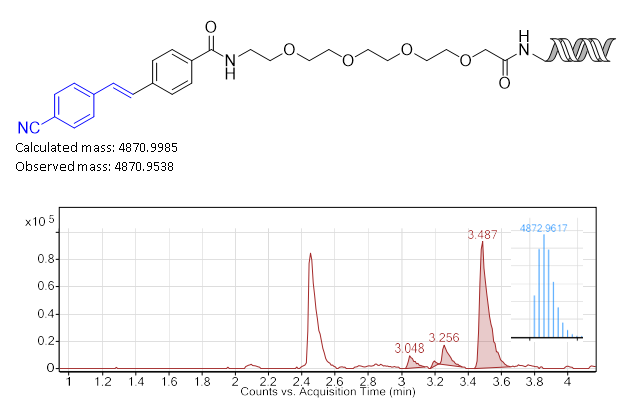

Supplement: Supplementary file 2 — bc3c00051_si_002.zip [file bc3c00051_si_002.zip › Chrom_Spectra/A63.PNG]

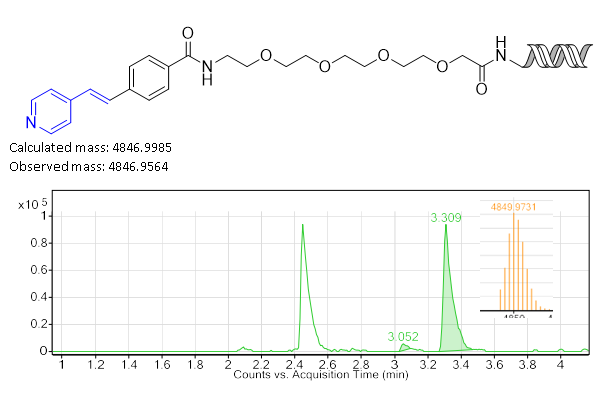

Supplement: Supplementary file 2 — bc3c00051_si_002.zip [file bc3c00051_si_002.zip › Chrom_Spectra/A64.PNG]

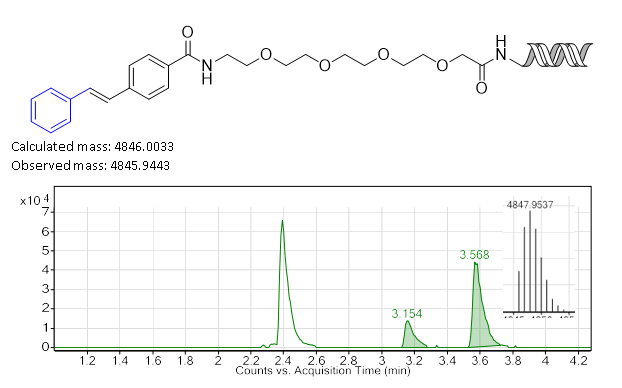

Supplement: Supplementary file 2 — bc3c00051_si_002.zip [file bc3c00051_si_002.zip › Chrom_Spectra/A72.PNG]

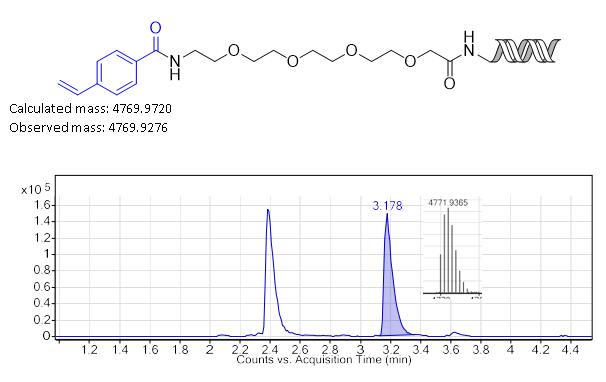

Supplement: Supplementary file 2 — bc3c00051_si_002.zip [file bc3c00051_si_002.zip › Chrom_Spectra/A71.PNG]

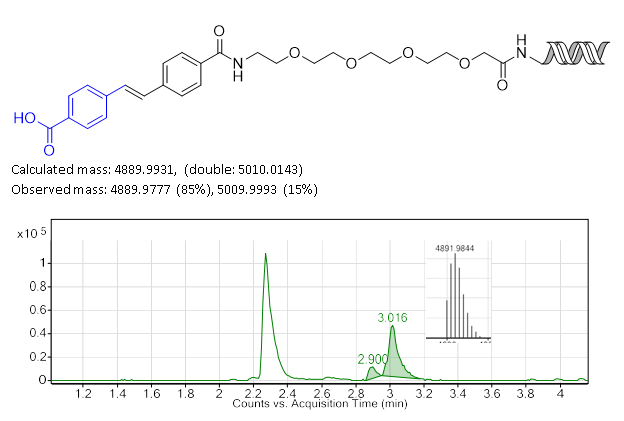

Supplement: Supplementary file 2 — bc3c00051_si_002.zip [file bc3c00051_si_002.zip › Chrom_Spectra/A73.PNG]

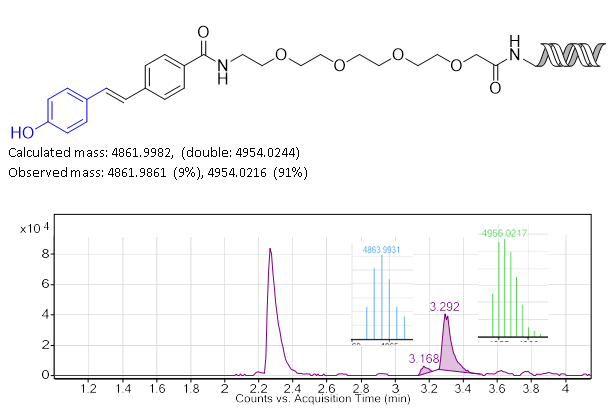

Supplement: Supplementary file 2 — bc3c00051_si_002.zip [file bc3c00051_si_002.zip › Chrom_Spectra/A74.PNG]

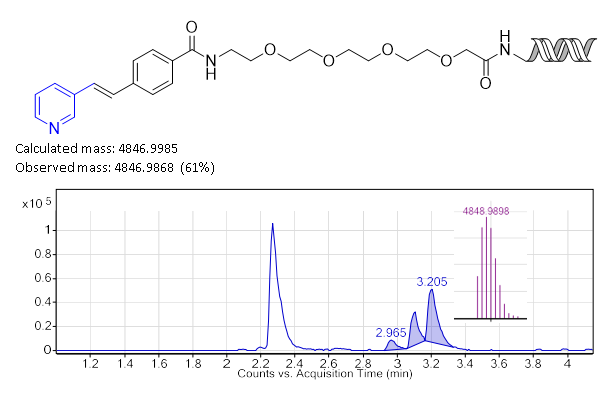

Supplement: Supplementary file 2 — bc3c00051_si_002.zip [file bc3c00051_si_002.zip › Chrom_Spectra/A75.PNG]

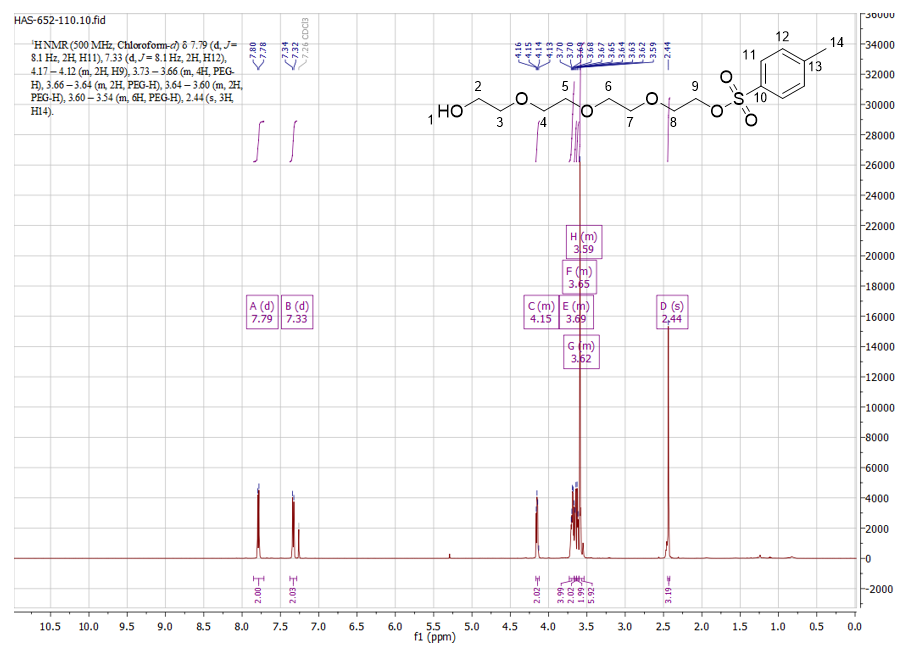

Supplement: Supplementary file 2 — bc3c00051_si_002.zip [file bc3c00051_si_002.zip › Chrom_Spectra/PEG4-Ts_1Hf.PNG]

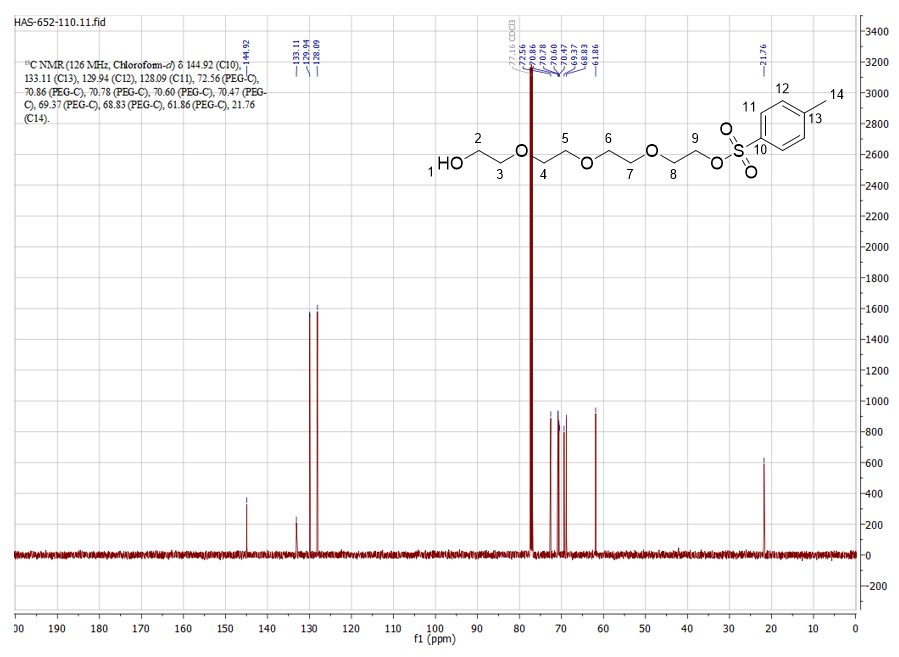

Supplement: Supplementary file 2 — bc3c00051_si_002.zip [file bc3c00051_si_002.zip › Chrom_Spectra/PEG4-Ts_13Cf.PNG]

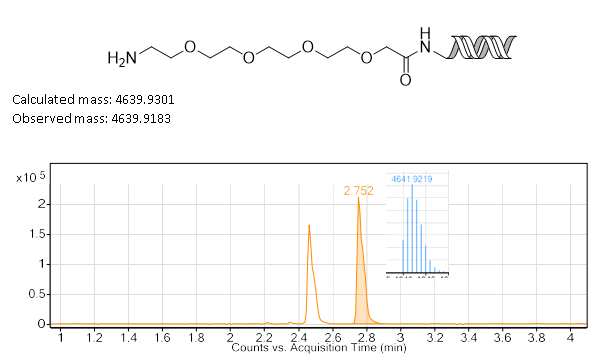

Supplement: Supplementary file 2 — bc3c00051_si_002.zip [file bc3c00051_si_002.zip › Chrom_Spectra/HP57.PNG]

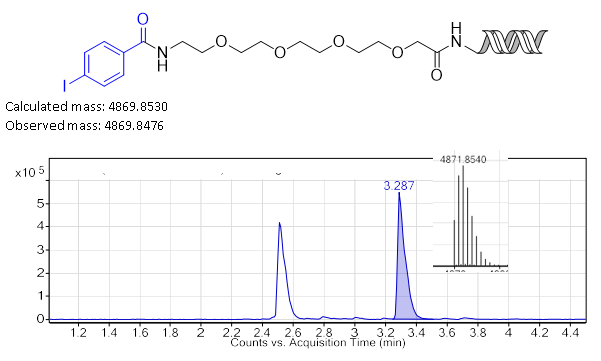

Supplement: Supplementary file 2 — bc3c00051_si_002.zip [file bc3c00051_si_002.zip › Chrom_Spectra/HP58.PNG]
